# Phenol-Driven Modulation Enables the Crystallization of Titanium Metal–Organic Frameworks

**DOI:** 10.1021/jacs.6c16169

**Published:** 2026-10-29

**Authors:** Pablo Ayala, Yan Zheng, Yu Xia, Cornelia von Baeckmann, Zheao Huang, Magdalena Ladisich, Sarah Hermetinger, Dogukan H. Apaydin, Alexey Cherevan, Guillermo Gómez-Tenés, Max Schmallegger, Georg Gescheidt, Freddy Kleitz, Maria Asghar, Florian Libisch, Natalia M. Padial, A. Ken Inge, Carlos Martí-Gastaldo, Tom Willhammar, Dominik Eder

**Affiliations:** † Institute of Materials Chemistry, 27259 Technische Universität Wien, Getreidemarkt 9, Vienna 1060, Austria; ‡ Department of Chemistry, 7675 Stockholm University, Frescativägen 8, Stockholm 106 91, Sweden; § Wallenberg Initiative Materials Science for Sustainability, Stockholm University, Stockholm 106 91, Sweden; ∥ Instituto de Ciencia Molecular (ICMol), 16781 Universitat de València, Catedrático José Beltrán 2, Paterna 46980, Spain; ⊥ Institute of Physical and Theoretical Chemistry, 27253 Graz University of Technology, Stremayrgasse 9/I, Graz 8010, Austria; # Department of Functional Materials and Catalysis, 27258 Faculty of Chemistry University of Vienna, Währinger Straße 42, Vienna 1090, Austria; ∇ Institute of Theoretical Physics, 541066 Technische Universität Wien, Wiedner Hauptstraße 8-10/136, Vienna 1040, Austria

## Abstract

Titanium-based metal–organic frameworks (MOFs) remain among the most challenging frameworks to synthesize due to the high reactivity of Ti­(IV) precursors, particularly when in combination with strongly coordinating phenolate ligands. Phenol is an untapped modulator for MOF synthesis that enabled the formation of a new crystalline Ti-MOF phase, otherwise unattainable through established modulators. We show how transient Ti-phenolate species formed in solution regulate titanium coordination equilibria and demonstrate the broader applicability of phenol modulation for several reported Ti-MOF systems. As a result of phenol modulation, we isolate a previously elusive crystalline titanium ellagate sustainable MOF, TUW-11, composed of monometallic Ti­(IV) centers and plant-derived ellagic acid linkers. Three-dimensional electron diffraction, synchrotron X-ray techniques, and scanning transmission electron microscopy reveal a layered microporous framework with 1.1 nm channels. TUW-11 exhibits a narrow band gap, p-type semiconducting behavior, and ligand-centered visible-light photoresponse. These findings demonstrate phenol modulation as a powerful strategy for controlling titanium-phenolate framework formation and expand the synthetic toolbox available for Ti-MOF discovery.

## Graphical abstract

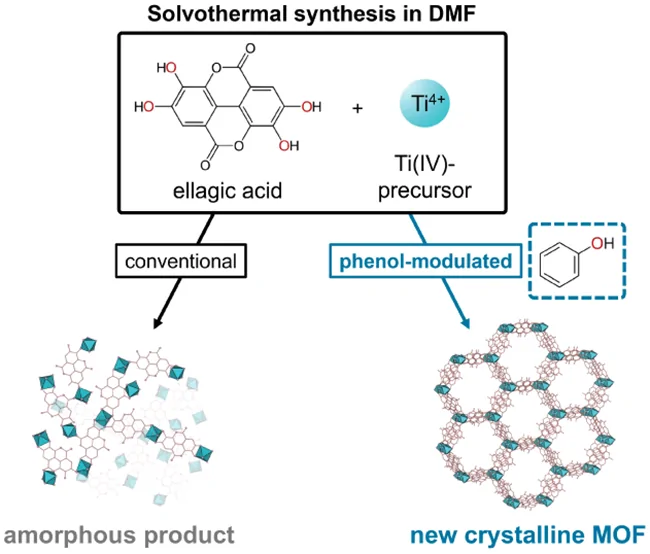

## Introduction

Titanium-based metal–organic frameworks (Ti-MOFs) have attracted considerable attention owing to their strong Ti–O bonds that provide hydrolytic stability and their photoactive properties, making them useful for environmental and energy applications.^1–5^ Despite these attractive characteristics, the number of reported Ti-MOF structures remains comparatively small. The high reactivity of Ti­(IV) precursors often leads to rapid hydrolysis and uncontrolled condensation during synthesis, making the isolation of crystalline framework materials particularly challenging.

These difficulties become even more pronounced when strongly coordinating phenolate or catecholate ligands are employed. The strong affinity of phenolate ligands toward Ti­(IV) often results in rapid coordination and the formation of poorly ordered solids.^6–8^ Ellagic acid (EA), a naturally occurring plant-derived polyphenol, represents a particularly demanding example because of its strongly chelating catechol groups.^9,10^ However, its natural origin and biocompatibility also make EA an attractive linker for the development of more sustainable MOF materials.^11,12^ While EA-based MOFs have recently been reported with several metal ions,^13–16^ the synthesis of a crystalline titanium ellagate framework has remained elusive.

To address the inherent reactivity of titanium precursors, several synthetic strategies have been developed, including the use of less reactive Ti sources or preformed titanium-oxo clusters (TOCs),^17–27^ *in situ* generation of reactive Ti^3+^ species,^28^ or coordination modulation. Among these, modulation with monocarboxylic acids has proven instrumental for controlling crystallization through competitive binding across many framework types.^29–31^ In Ti-MOF chemistry, both monocarboxylic acids and strongly chelating modulators have been employed successfully.^32–36^ However, strongly chelating modulators may not provide the weak, reversible coordination dynamics required for the controlled assembly of some demanding Ti-phenolate systems.

An ideal modulator for Ti-phenolate MOFs should therefore bind strongly enough to suppress uncontrolled precipitation, while remaining sufficiently labile to allow framework self-assembly. Although phenolic compounds have been established in Ti coordination and sol–gel chemistry,^37–40^ phenol itself has not been explored—to the best of our knowledge—as a modulator for MOF synthesis. We hypothesized that the weaker, reversible Ti-phenolate interactions could regulate titanium coordination equilibria in solution, facilitating controlled framework crystallization of otherwise inaccessible Ti-phenolate MOFs.

Here, we demonstrate the use of phenol as a modulator for Ti-MOF synthesis and elucidate its role in controlling Ti-MOF crystallization through the formation of transient Ti-phenolate species that reversibly coordinate Ti­(IV) centers in solution, avoiding rapid formation of amorphous products. This approach enables access to a new crystalline and microporous titanium ellagate framework, TUW-11 (TUW = Technische Universität Wien), and improves the crystallinity of several reported Ti-MOF systems with related coordination environments. These findings establish phenol modulation as a versatile strategy for controlling the crystallization of titanium phenolate frameworks, expanding the synthetic toolbox available for Ti-MOF discovery.

## Results and Discussion

### Phenol Modulation Enables the Crystallization of Ti-MOFs

Combining Ti­(IV) precursors with strongly chelating linkers such as EA often results in rapid and uncontrolled precipitation of red amorphous products, hindering the formation of crystalline framework materials during solvothermal reaction. To overcome this challenge, synthetic conditions, including precursor ratios and concentrations, modulator identity and amount, solvent, temperature, and reaction time, were screened systematically and optimized with particular emphasis on modulation strategies (Supporting Information, Section 3), ultimately leading to the identification of phenol as an effective modulator for the synthesis of a new crystalline titanium-ellagate MOF phase, TUW-11. Modulators described previously in Ti-MOF chemistry yielded materials possessing low crystallinity (when using acetic acid), secondary phases (when using catechol) or no solid product at all (when using salicylic acid), as shown in Figure S3. The implementation of phenol as a reversibly coordinating modulator, in contrast, resulted in the highly crystalline, well-defined hexagonal particles of TUW-11 (Figure 1 and Figure S8). These were obtained through solvothermal reaction (at 120 °C for 48 h) of EA with titanium­(IV) isopropoxide (TTIP) in absolute *N*,*N*-dimethylformamide (DMF). Using phenol modulation, TUW-11 could also be obtained using other Ti­(IV) precursors, such as titanocene dichloride (Cp_2_TiCl_2_) or titanium acetylacetonate (Ti­(acac)_2_) as shown in Figure S15.

**Figure 1:**
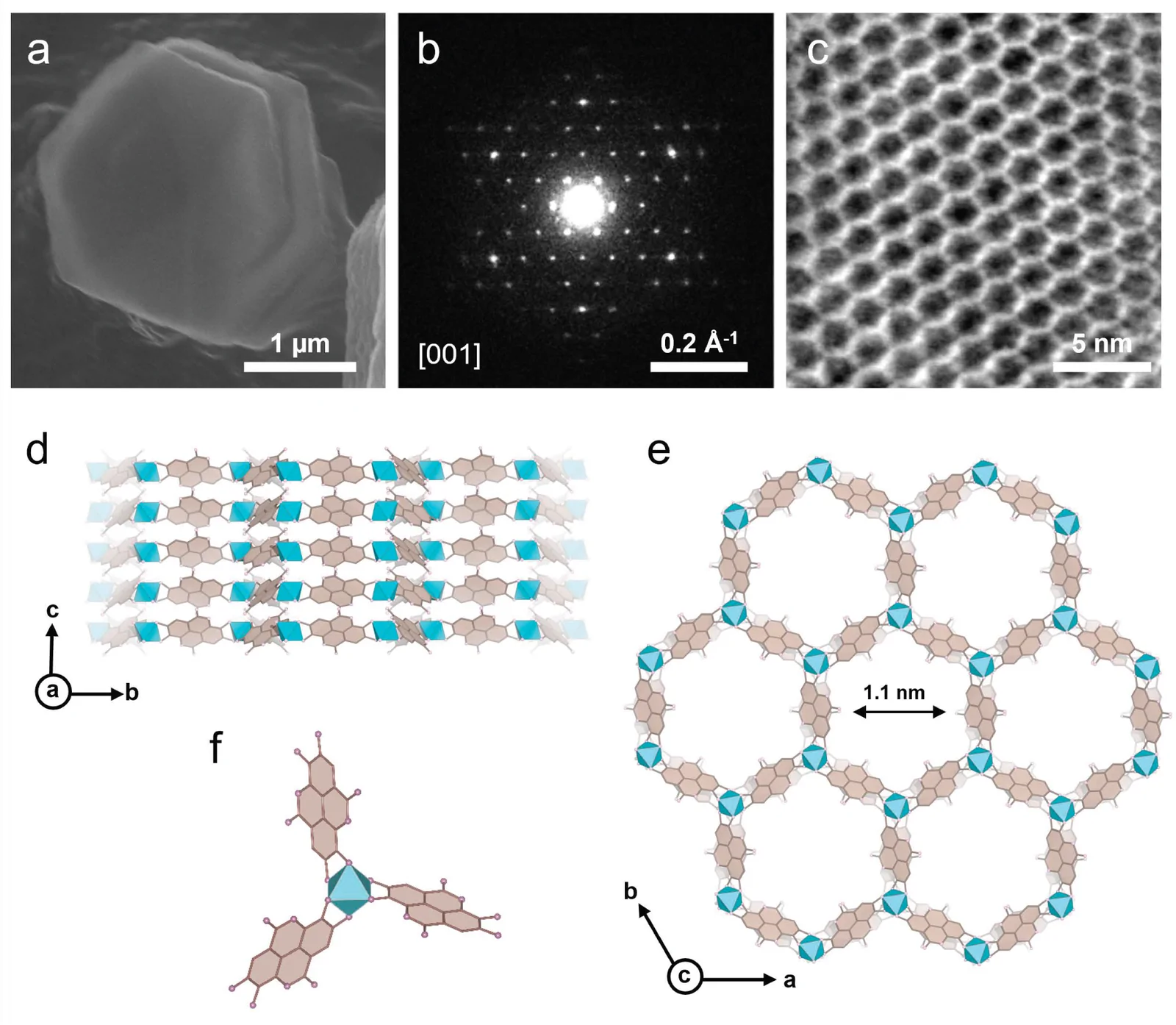
(a) SEM image of as-synthesized TUW-11. (b) Electron diffraction pattern of TUW-11 along the [001] zone axis. (c) iDPC-STEM image of TUW-11 viewed along [001], revealing the pore structure directly. (d) Structure model of TUW-11 along the *a*-axis projection showing the layered structure assembling through π–π interactions. (e) Projection along the *c*-axis displaying the microporous 1.1 nm 1D channels of TUW-11. (f) Coordination environment of the Ti^4+^ center.

We propose that the role of phenol in the synthesis of TUW-11 involves a competitive, yet reversible coordination to titanium cations in solution. To examine this hypothesis, we evaluated commonly used modulators in titanium (carboxy)-phenolate MOF chemistry, including acetic acid, methanol, catechol, oxalic acid and salicylic acid (for details, see Table S1). Mixing acetic acid or methanol into the MOF precursor mixture (TTIP and EA in DMF) led to immediate precipitation (Figure S18), while phenol retained Ti species in the solution (Figure S19), similar to the effect of strongly chelating modulators (oxalic acid, catechol) observed in Figure S20 and previously reported for Ti systems.^32,36^

Increasing the phenol content led to a progressive intensification of the supernatant’s color (Figure S21), suggesting Ti-phenolate interactions. To probe the nature of this coordination and its reversibility, *in situ* titration experiments were performed in DMF-*d*_7_ using NMR spectroscopy at room temperature.

Upon mixing phenol with TTIP, the ^1^H NMR spectra (Figure 2a, **pink**) show the disappearance of the phenolic OH signal at 9.7 ppm (H_a_), indicating phenolate coordination to the Ti center. This is accompanied by the emergence of a signal at 4.5 ppm (Figure S23) corresponding to the OH of isopropanol, indicating alkoxide exchange as illustrated in the reaction scheme (Figure 2), like previously reported.^37^ Concurrently, the aromatic resonances of phenol broaden and the H_c_ and H_d_ positions shift by ∼0.1 ppm upon coordination with Ti (Figure 2a, **pink**), while the H_b_ position stays unchanged. The position-dependent shifts are attributed to the interplay of inductive and resonance effects associated with the formation of Ti-phenolate species resulting in a perturbation of the aromatic electronic environment.^41,42^ Stepwise addition of EA (Figure 2a, **yellow to dark blue**) restores the characteristic OH and aromatic resonances of free phenol, consistent with displacement of coordinated phenol by EA. Signals corresponding to coordinated isopropoxide (1.4 ppm for H_e*_) stemming from the TTIP decrease stepwise with each addition of EA, while the free isopropanol (signal of H_f_ at 1.3 ppm) increases proportionally.

**Figure 2:**
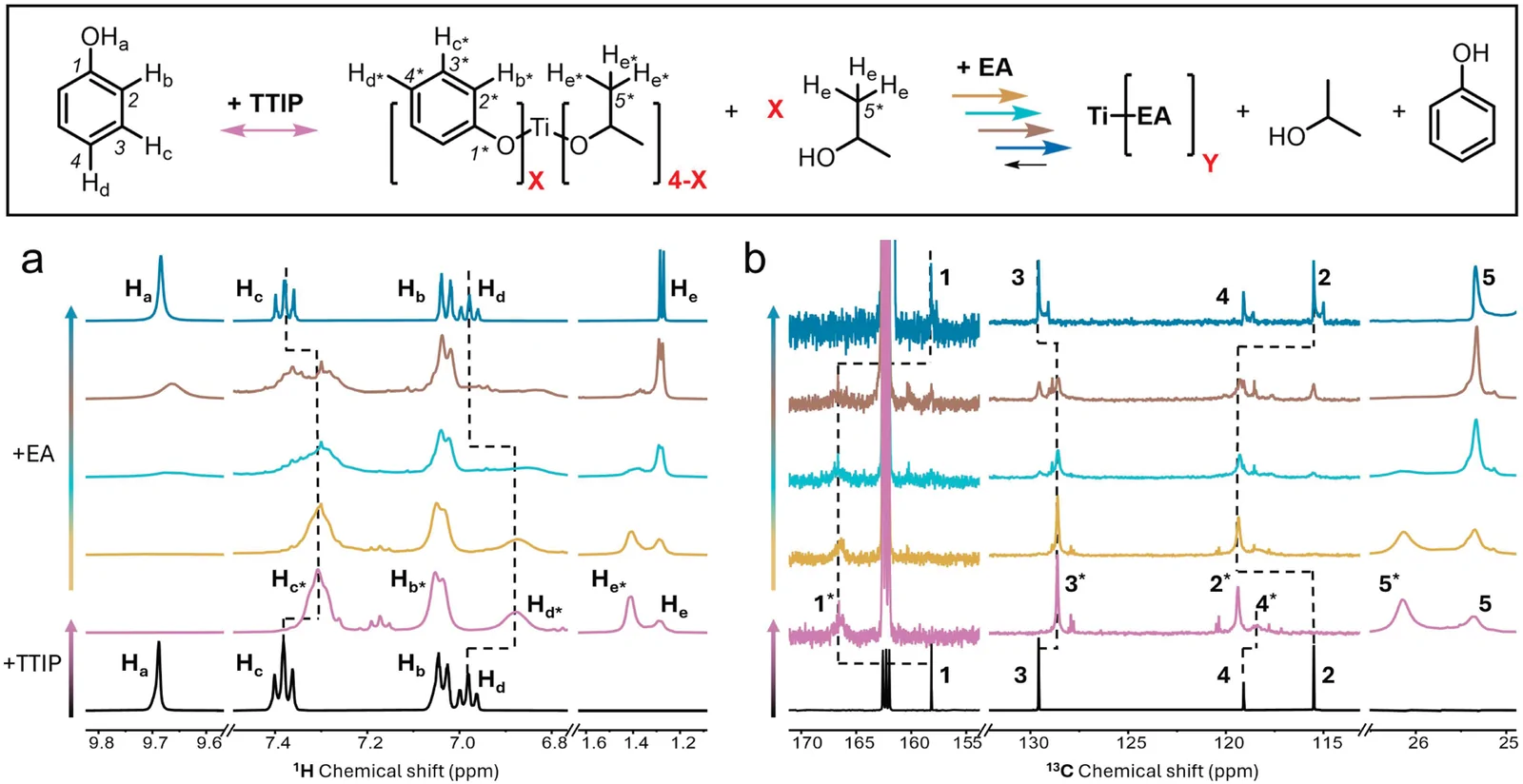
*In situ* NMR analysis of the reaction by stepwise addition of the reactants and the proposed reaction scheme. (a) ^1^H NMR spectra normalized to the DMF signal showing the formation of the Ti-phenolate complex (pink) due to signal broadening and a shift of the corresponding proton signals and (b) the corresponding ^13^C NMR spectra. **Black**: pure phenol; **pink**: +TTIP; **yellow**: +1 mg EA; **light blue**: +2 mg EA; **brown**: +3 mg EA; **dark blue**: +4 mg EA. Full spectra can be found in the Supporting Information, Figures S22–S27.

The ^13^C NMR spectra (Figure 2b) support this interpretation. Upon coordination (Figure 2b, **pink**), the C_1_ and C_2_ signals shift downfield by 8 and 4 ppm, respectively, whereas C_3_ and C_4_ shift slightly upfield (∼1 ppm). Similar opposing chemical shifts have also been reported for metal-coordinated phenoxide ligands, further supporting Ti-phenolate coordination.^43^ Addition of EA (Figure 2b, **yellow to dark blue**) shifts the coordination equilibrium and finally restores the original ^13^C signature of free phenol (Figure 2b, **dark blue**). Similar to the ^1^H NMR spectra, the decrease of the C_5*_ signal (isopropoxide) is accompanied by an increase of the distinct C_6_ signal (free isopropanol as a leaving group).

These results demonstrate a competitive coordination equilibrium between phenolate and ellagate ligands at Ti centers, thereby suggesting the influence of phenol in regulating ligand exchange equilibria in solution at room temperature. Importantly, our optimization experiments (Supporting Information, Section 3) show that elevated temperatures are essential for the crystallization of the TUW-11 phase, which was otherwise impossible with other modulators (methanol, catechol, acetic, oxalic, squaric, and salicylic acid) investigated in this study. While elevated temperatures likely influence Ti-phenolate equilibria and exchange kinetics, the precise speciation and framework formation behavior under solvothermal conditions remains to be investigated.

The general applicability of this approach was examined for several reported Ti-MOFs with similar binding motifs (Figure 3). For Ti-CAT-5, a catecholate-based MOF derived from hexahydroxytriphenylene (HHTP),^34^ the phenol-assisted synthesis yielded sharper and more intense diffraction peaks. The full-width at half-maximum (FWHM) values roughly halve (Table S3) and particle sizes increase, from submicrometer to ∼5 μm (Figure S28). Similar trends were observed for IEF-11, a Ti-MOF based on squaric acid,^44^ where improved crystallinity was accompanied by the formation of larger particles. Both systems are known to be challenging to crystallize as large single crystals and have previously been structurally resolved only by powder X-Ray diffraction (PXRD) or three-dimensional electron diffraction (3D ED) of nanoscopic particles aided by computational modeling.

**Figure 3:**
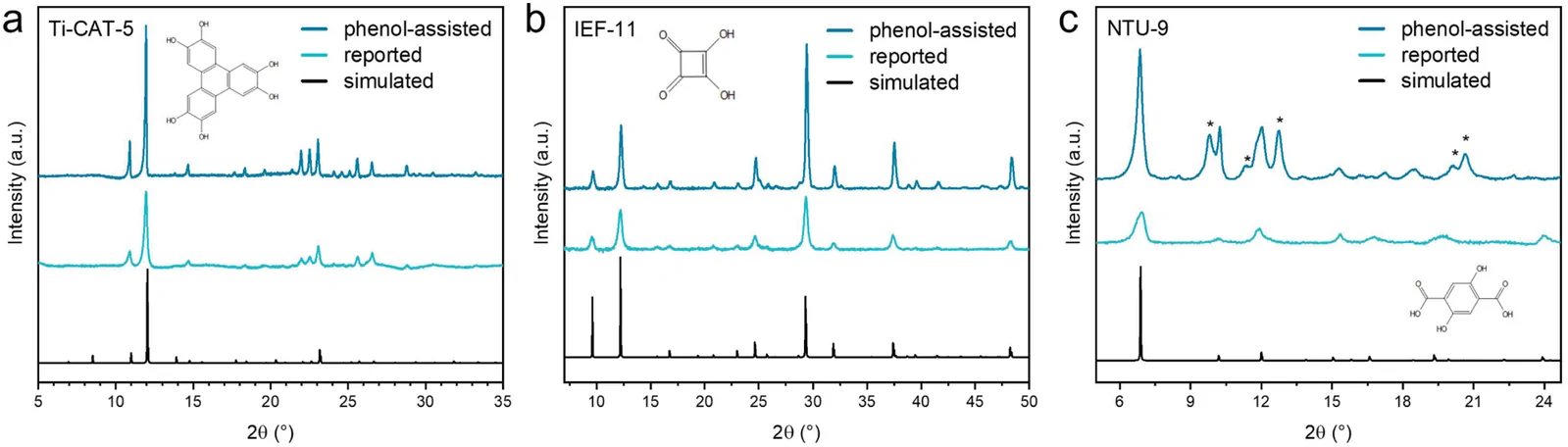
PXRD patterns of the obtained powders using the reported vs. the phenol-assisted syntheses for Ti-CAT-5 (a), IEF-11 (b) and NTU-9 (c). The stars in (c) show the coexistence of another phase, which has been identified as FIR-119.

Further tests on Ti-MOFs based on carboxy-phenolate (NTU-9)^33^ chelating coordination showed more limited effects. For NTU-9, improved crystallinity of the target phase was obtained, albeit alongside the formation of an additional phase (FIR-119), consistent with the known polymorphism of the Ti-DOBDC (2,5-dihydroxyterephthalic acid) system, in which up to 8 phases have been reported utilizing different synthetic methods to date.^32,33,36,45^ Notably, FIR-119 has also been obtained using chemically distinct coordinating modulators, suggesting that changes in coordination of titanium intermediates can alter the balance between competing Ti-DOBDC assembly pathways.

To further explore the scope of phenol modulation, we extended the strategy to Ti-MOFs without phenolate coordination, but with distinct carboxylate or hydroxamate coordination. The desired COK-47­(bdc)^18^ and MUV-11 ^35^ phases could also be obtained through phenol-assisted synthesis (Figure S29), although no clear overall improvement in crystallinity was observed compared with the reported procedures (Table S3). Overall, the effectiveness of phenol modulation varies across frameworks with distinct binding modes, with its most pronounced effects observed for catecholate-based Ti-MOFs. This highlights the importance of matching modulator and linker coordination chemistry to achieve effective competitive and reversible coordination.

### Structural Characterization of TUW-11

The bright red TUW-11 crystallites (Figure S30) synthesized with phenol as the modulator show sharp and well-defined peaks in its PXRD pattern, confirming the successful synthesis of a crystalline product (Figure 4a). Scanning electron microscopy (SEM) images of TUW-11 reveal a hexagonal morphology with particles of 2–3 μm in diameter and 200 nm in thickness (Figure 1a). Due to their small size, a crystal structure model was established using electron crystallographic methods, including 3D ED,^46,47^ more specifically continuous rotation electron diffraction (cRED), and scanning transmission electron microscopy (STEM) imaging.^14,48,49^ As shown in Figure 1b, a representative electron diffraction pattern along the [001] zone axis exhibits hexagonal symmetry, suggesting that the material belongs to the hexagonal crystal family, including both the hexagonal and trigonal crystal systems. From the three-dimensional reconstruction of one cRED data set (Figure S31), the unit cell parameters (*a* = *b* = 19.12 Å, *c* = 9.53 Å, α = β = 90°, γ = 120°) were determined. This unit cell was further verified and refined by a Pawley fit against synchrotron PXRD data (*a* = *b* = 18.97 Å, *c* = 9.19 Å, α = β = 90°, γ = 120°), as shown in Figure 4a with fitting details provided in Table S4. However, diffuse streaks along the c*-axis, indicating the presence of interlayer disorder, together with limited resolution, prevented *ab initio* structure solution from the cRED data. Hence, STEM imaging was utilized to better understand the structure. Both annular dark-field (ADF) and integrated differential phase contrast (iDPC) STEM images under low dose conditions reveal the local porous structure of the TUW-11 crystals. The ADF-STEM image along the [001] direction (Figure S32), highlights that the Ti^4+^ cations are located at the vertices of the hexagonal pores. The iDPC-STEM images provide better contrast to lighter elements, which further reveals the organic ligands bridging the metal centers (Figure 1c). In projection, this implies that each metal node is coordinated by three ligands, forming a hexagonal porous structure. In most regions of the crystals, these layers are aligned along the *c*-axis to generate 1D channels. However, some of the hexagonal pores appear to be distorted and locally compressed, deviating from the ideal hexagonal geometry (Figure S33).

**Figure 4:**
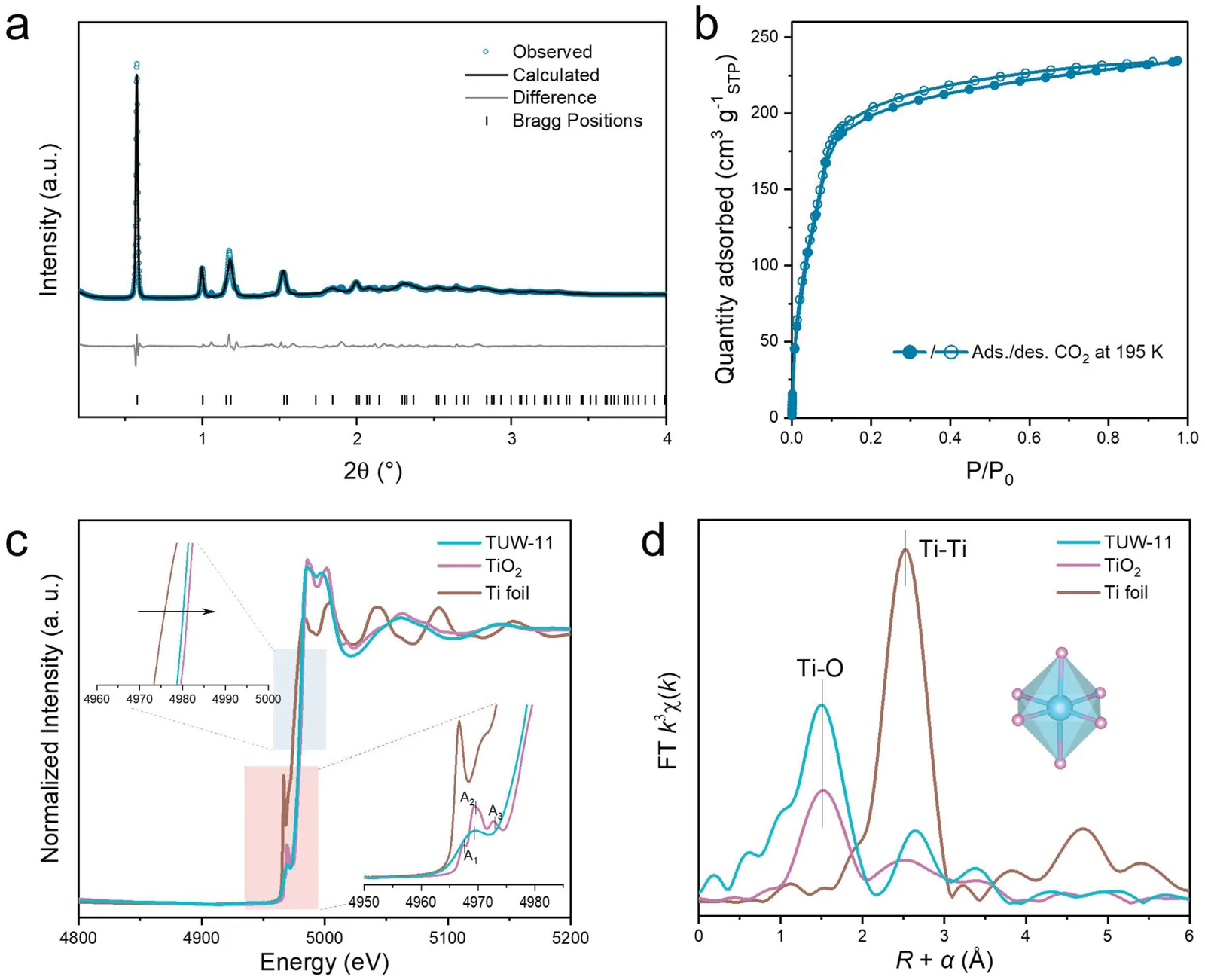
(a) The Pawley fitting plot of synchrotron high-resolution PXRD data. (b) CO_2_ physisorption isotherm at 195 K with adsorption and desorption branches. (c) Ti K-edge XANES of TUW-11 and reference standards Ti foil and TiO_2_ (anatase). (d) Fourier-transformed (FT)-EXAFS k^3^-weighted χ­(*k*) function spectra of TUW-11 and reference standards Ti foil and TiO_2_ (anatase). The **Inset** is the Ti coordination model in TUW-11, where blue represents Ti and pink represents O.

Based on these results, an idealized structure model in space group *P*3̅1*c* was constructed (Figure 1d-f). The model comprises 2D honeycomb (hcb) layers of mononuclear Ti centers coordinated by three ellagate ligands and extending in the *ab*-plane. These layers assemble through π–π interactions, forming an eclipsed stacking that results in 1D channels along the *c*-axis with a 1.1 nm pore diameter. The simulated diffraction pattern derived from this model shows good agreement with the PXRD data (Figure S34), albeit with some minor differences that can be attributed to inter- and intralayer disorder, such as variations in the packing of adjacent layers as well as local disorder within layers, locally resulting in domains of distorted pores, as observed in the STEM images.

X-ray absorption near-edge structure (XANES, Figure 4c) spectra further verify the proposed coordination environment of the Ti center. The Ti K-edge of TUW-11 closely coincides with that of TiO_2_, indicating an octahedral coordination geometry with a Ti^4+^ oxidation state, further corroborated with X-ray photoelectron spectroscopy (XPS) in Figure S35. Notably, the pre-edge feature of TUW-11 (Figure 4c, **Inset**) at 4968 eV closely matches the A_2_ feature observed for TiO_2_. In general, pre-edge components (A_1_–A_3_) in TiO_2_ originate from transitions of Ti 1s orbitals to Ti 3d-4p hybridized states and the relative intensity of A_2_ increases when the TiO_6_ octahedron deviates from perfect centrosymmetry.^50,51^ This suggests a local ligand field around Ti, which induces sufficient 3d–4p orbital mixing in TUW-11, well in line with the deviation from perfect centrosymmetry of the Ti center in the structure model. Extended X-ray absorption fine structure (EXAFS) shown in Figure 4d and Figure S36 and the resulting fittings (Table S5) further support this conclusion, showing two distinct first-shell Ti–O scattering paths at 1.98 Å (coordination number ≈ 2.9) and 2.01 Å (coordination number ≈ 3.0). Notably, DFT relaxation (see Computational methods) of the proposed structure model yields Ti–O distances ranging from 1.89 to 2.09 Å, consistent with a distorted TiO_6_ octahedron, and an average distance of 1.98 Å, in good agreement with the average experimental distance of 2.00 Å obtained from EXAFS (see Table S9). Moreover, unlike typical Ti-MOFs containing polymetallic oxoclusters (e.g., Ti_8_O_8_(OH)_4_ in MIL-125 ^52^) that exhibit a prominent second-shell Ti–Ti scattering peak, TUW-11 lacks such oxidic Ti–Ti signals in the higher R region. The minor features observed are assigned to long-range Ti–C scattering from the organic linkers. Combined with the absence of any metallic Ti–Ti signal (i.e., Ti foil), this suggests that the Ti sites in the MOF framework are isolated without any metallic or oxidic aggregation. Together, these results support the plausibility of the proposed structure model of TUW-11, consisting of isolated, six-coordinated, slightly distorted TiO_6_ octahedra as the inorganic nodes.

To gain further macroscopic insights into TUW-11 and potential guest molecules within its pores, we performed NMR spectroscopy, thermogravimetric analysis (TGA), and elemental analysis. The acid digestion ^1^H NMR spectrum of the as-prepared TUW-11 shows a singlet at 7.57 ppm arising from the only aromatic proton at the linker (Figures S40 and S41). Residual phenol from the synthesis, either in the structure or in the pores, can be excluded due to the lack of multiplets in the region of 6.5–7.5 ppm. However, regardless of the washing procedure, a peak at 2.47 ppm was consistently found and is related to dimethylammonium (DMA) cations (Figure S41). The relative ratios indicate two DMA molecules per formula unit. These are likely to act here as charge balancing cations and align well with the Ti^4+^ oxidation state of the central atom discussed above (Figure 4c and Figure S35), surrounded by three EA molecules (net charge −6). Besides the DMA cations, the results show residual DMF molecules from the synthesis in the pores, even after vacuum activation. We found acetone to be an ideal solvent for removing residual DMF (Figure S41) in the pores while also preserving the framework unaltered (Figure S42). The experimental mass loss of activated TUW-11 recorded through TGA (Figure S43) shows an initial step (1) ascribed to adsorbed species and a second main step (2) equal to 86 wt % relative to the first plateau, which is consistent with the expected organic mass loss of the proposed formula (C_2_H_8_N)_2_[Ti­(C_14_H_2_O_8_)_1.5_] (theoretical = 86.4 wt %). Elemental analysis further supports this finding, with quantified mass percentages of C = 50.8, H = 3.74, N = 4.51, and O = 29.5 wt % (theoretical C = 51.1, H = 3.24, N = 4.77, and O = 32.7 wt %). Importantly, these DMA cations could not be found through diffraction techniques, implying disorder in their distribution within the pores. Cation exchange experiments (details in Figure S44) with K^+^ further corroborated a 1:2 ratio of Ti to DMA by quantifying the Ti to K ratio through TXRF after exchange and thorough washing. The material remains stable in aqueous media between pH 3 and 11, as indicated by PXRD and leaching experiments (Supporting Information, Section 5.12).

Common vacuum activation protocols for removing residual solvent molecules in the pores led to structure collapse due to capillary stress effects. Hence, we opted for a supercritical CO_2_ activation protocol prior to adsorption measurements. In short, the acetone-exchanged samples were subjected to repeated liquid CO_2_ solvent exchange, brief supercritical treatment and slow venting (details in Supporting Information, Section 1.2). The sorption studies with N_2_ (at 77 K) show a moderate BET surface area and strong hysteresis, likely associated with interparticle voids (supplementary isotherms in Figures S47 and S48) and underestimate the surface area and pore volume expected from the idealized structure model with 1.1 nm micropores. This is likely due to the known diffusion restrictions of N_2_ as a probe molecule for materials with small pores.^53,54^ In contrast, the adsorption–desorption isotherm at 195 K using CO_2_ (a molecule with smaller kinetic diameter and significantly higher quadrupole moment) exhibits a Type I profile with negligible hysteresis (Figure 4b), characteristic of a predominantly microporous material. The resulting BET surface area of 622 m^2^ g^–1^ and pore volume of 0.36 cm^3^ g^–1^ are well in line with the expected values (see Supporting Information, Section 5.13) and suggest a stronger affinity for CO_2_, likely due to the unique pore chemistry of ellagate frameworks.

### Optoelectronic Properties of TUW-11

TUW-11’s deep red color prompted us to investigate its optoelectronic properties. Diffuse reflectance spectroscopy (DRS) revealed an extended absorption range in the visible spectrum (Figure 5a), compared to the bare linker or the mononuclear Zr-EA MOF, SU-102.^14^ Further comparisons to other visible-light-absorbing Ti-phenolate-based MOFs can be found in Figure S49 and Table S7. The Tauc plot yielded an optical band gap (*E*_g, opt._) of 1.68 ± 0.05 eV (Figure 5a, **Inset**)—the narrowest among other EA-MOFs reported to date, which are all in the range of 2.40–2.70 eV—opening the possibility of using low energy photons for exciton generation. Cyclic voltammetry (Figure S53) of TUW-11-coated electrodes (preparation in Supporting Information, Section 1.2. and characterization in Figures S50–S52) revealed onset potentials of 1.08 V for oxidation and −0.45 V for reduction, yielding a HOCO/LUCO electronic band gap (*E*_g, el._) of 1.54 eV (calculation in Supporting Information, Section 1.2), which is in good agreement with the *E*_g, opt._ estimated through DRS.

**Figure 5:**
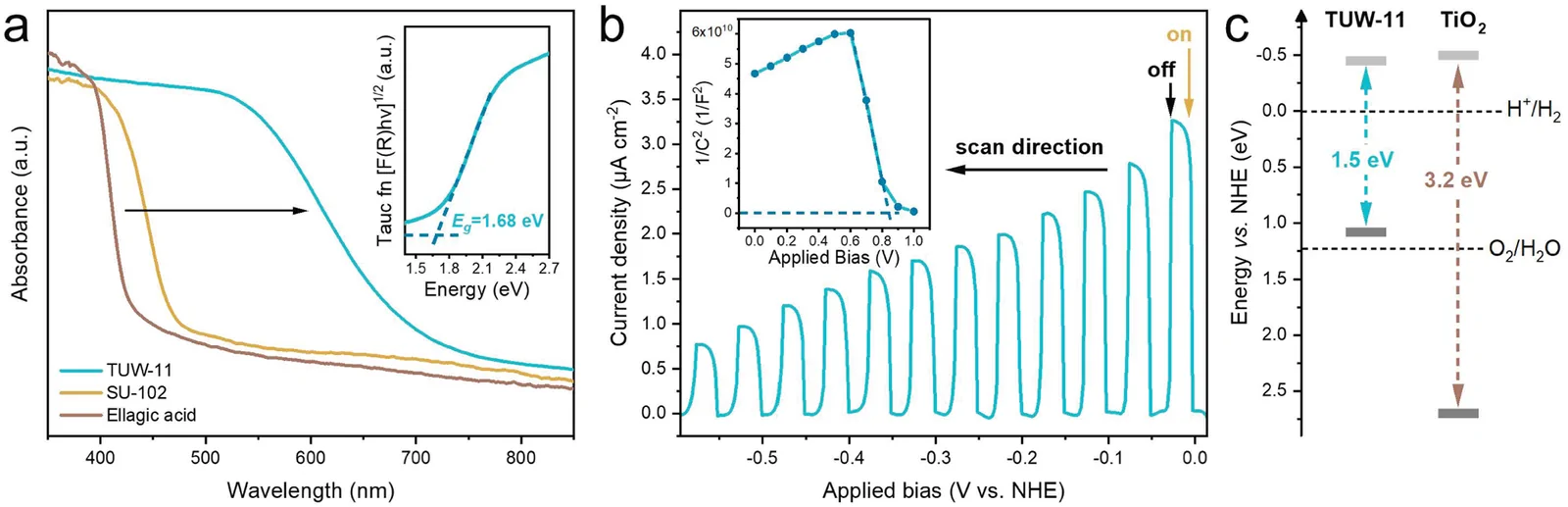
(a) DRS spectra of ellagic acid, SU-102, and TUW-11. **Inset**: Kubelka–Munk theory calculated Tauc function for *E*_g, opt._ determination. (b) Chopped light linear sweep voltammetry measurements of TUW-11-coated FTOs showing TUW-11’s photoresponse under negative bias. **Inset**: Mott–Schottky plot at 1000 Hz with a negative slope (p-type) and a flat band potential of 0.84 V. (c) Cyclic-voltammetry-derived energy diagram showing the HOCO/LUCO alignment of TUW-11 with TiO_2_ (value from Sakai et al.^55^) and water-splitting redox couples as references.

TUW-11 demonstrates a remarkable photoresponsive behavior during chopped light (λ > 420 nm, 0.5 W) linear sweep voltammetry measurements compared to other reported phenolate-coordinated Ti-MOFs (see Figure S54 and Table S8). Figure 5b shows photocurrents reaching ≈3 μA cm^–2^ at zero-bias and ≈1 μA cm^–2^ under negative applied bias, expected for a p-type semiconducting material. The negative slope of the multifrequency Mott–Schottky plot (Figure 5b, **Inset** and Figure S55) further corroborates this p-type behavior, indicating the holes as the majority charge carriers with a flat band potential of 0.86 V. Interestingly, aligning the HOCO/LUCO levels vs. NHE (Figure 5c), it becomes evident that photocatalytic overall water splitting cannot be achieved due to the less positive HOCO level of TUW-11, compared to TiO_2_. The hydrogen evolution half reaction, on the other hand, should be thermodynamically feasible. However, we observed negligible amounts of H_2_ produced by TUW-11 under illumination, probably due to the small energetic driving force for proton reduction and, more importantly, the absence of photogenerated Ti^3+^ species. Upon irradiation, electron paramagnetic resonance (EPR) spectroscopy measurements (Figure S56a) indicate a substantial increase of an unresolved EPR signal at *g* = 2.02, attributable to oxygen-trapped holes at the linker.^56–58^ Markedly, no signal pointing to paramagnetic Ti^3+^ (Figure S56b) is detected after illumination, compatible with previous reports on monometallic, chelate-coordinated metal center MOFs.^35^ The observed photoresponse is therefore dominated by the photophysics of the ellagic acid linker, rather than by ligand to metal charge transfer (LMCT). Unlike carboxylate-based Ti-MOFs (e.g., MIL-125), TUW-11 displays negligible radiative emission after irradiation, as photoluminescence spectra (Figure S57) show. Furthermore, the time-resolved emission spectroscopy (TRES) measurements reveal a short average excited-state lifetime of 0.3 ns (Figure S58). The calculated electronic structure (details in the Supporting Information) further supports the optoelectronic properties of TUW-11. The calculated density of states (DOS, Figure S59a) yields an electronic band gap of approximately 1.7 eV, in good agreement with the experimentally determined optical band gap. Importantly, the Kohn–Sham states corresponding to the experimental HOCO and LUCO (see isosurface plot, Figure S59b) are predominantly localized on the ellagate linker, suggesting that the lowest-energy electronic transition is largely ligand-centered. Taken together, these findings consistently point to a ligand-centered excited state in TUW-11 that does not undergo efficient LMCT, rationalizing both the absence of Ti^3+^ formation and the short excited-state lifetime.

### Selective Adsorption and Linker-Centered Photoconversion

The short-lived nature of the photoexcited charge carriers in TUW-11 implies that efficient substrate conversion can only occur when charge transfer outcompetes exciton recombination. We therefore hypothesized that selective adsorption and intimate adsorbate-linker contact would be critical prerequisites for photoinduced reactivity.

To evaluate this concept, we first investigated the adsorption properties of TUW-11. Owing to its pore structure and negatively charged framework (ζ = −28.3 mV at neutral pH, Figure S61), TUW-11 exhibits the strongest affinity toward the cationic dye methylene blue (MB) among the investigated Ti-MOFs, reaching adsorption capacities of up to 616 mg g^–1^ (Figure 6a). In contrast, the anionic dye methyl orange (MO) showed a significantly lower uptake of 140 mg g^–1^ (Figure 6a, **Inset**), highlighting the importance of electrostatic interactions (chemical structures in Figure S62).

**Figure 6:**
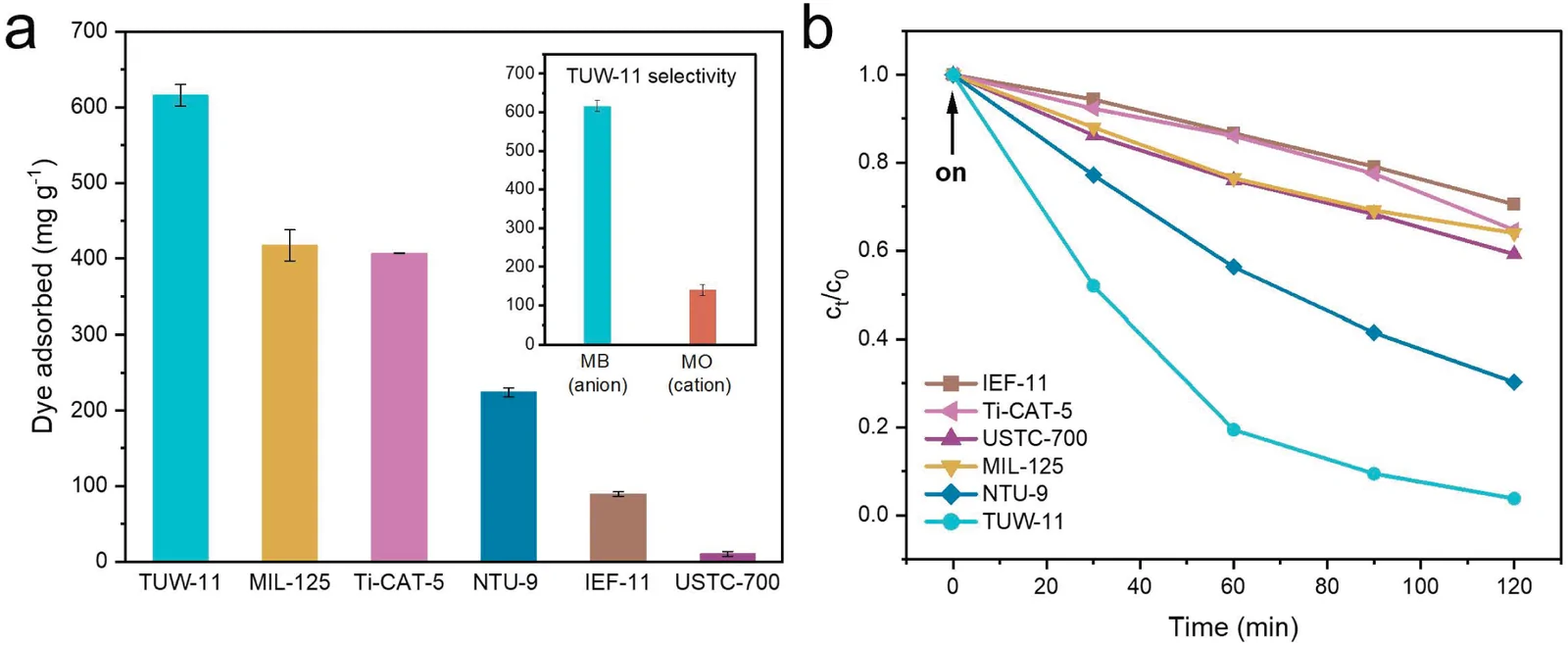
(a) MB adsorption using different Ti-based MOFs and their average adsorption capacity (*n* = 3, standard deviation plotted as error bars). **Inset:** Selective adsorption of TUW-11 toward negatively charged organic dye molecules. (b) Light-assisted dye removal using different Ti-based MOFs under 445 nm light illumination.

In subsequent dye removal experiments under visible-light irradiation (445 nm) after equilibration, TUW-11 displayed the highest degree of light-assisted MB removal (96% in 2 h) within the investigated MOF series (Figure 6b). Control experiments performed under otherwise identical dark conditions confirmed that MB removal is predominantly adsorption-driven, while consistently revealing an additional light-induced contribution (Figure S67 and Table S10). Importantly, the reported reaction rates were calculated exclusively from this light-induced contribution, excluding MB removed through adsorption (Figure S69 and Table S11). Conversely, no measurable conversion of MO was observed (Figure S63), despite identical irradiation conditions. The pronounced difference between both dyes suggests that close adsorbate-linker contact is critical for the observed photoconversion, rather than the generation of freely diffusing reactive oxygen species. Spin-trapping (ST) EPR spectroscopy measurements with 5,5-dimethyl-1-pyrroline *N*-oxide (DMPO, Figures S64 and S65) further support this interpretation, as no reactive oxygen species could be detected under irradiation, in contrast to analogous experiments with MIL-125, in which radical species could indeed be detected. This observation is consistent with the high-lying HOCO level of TUW-11 (1.08 V vs. NHE, Figure 5c), which is insufficient to drive hydroxyl-radical formation^59,60^ under the investigated conditions. However, control photoirradiation experiments (Figure S68) show a decrease in dye removal when less oxygen is available, as previously described.^61^ Together with the absence of photogenerated Ti^3+^ species and the short charge-carrier lifetimes observed by photo-EPR and TRES, these findings support a linker-centered photoconversion process, consistent with interfacial charge transfer from the photoexcited ellagate linker to strongly adsorbed substrate molecules.^62^

Recyclability experiments revealed a pronounced decrease in dye removal efficiency after the first cycle (Figure S70), accompanied by partial loss of framework crystallinity and persistent MB-specific FTIR signals in the recovered material (Figures S71–S72). This suggests that both structural degradation and irreversible binding of MB or its conversion products limit recyclability, although only negligible Ti leaching was detected in the liquid (Table S12). These limitations could potentially be mitigated by longer illumination times to promote further conversion of strongly adsorbed substrate molecules and more effective washing or reconditioning protocols.

While MB removal was employed here as a model reaction, the results illustrate how the combination of selective adsorption and linker-centered photoactivity can be leveraged to promote the light-assisted removal of organic pollutants. Unlike many Ti-MOF photocatalysts, where the metal centers act as the primary photoactive sites, the reactivity of TUW-11 appears to originate from the organic linker itself.

## Conclusions

In this work, we demonstrate phenol as an effective and previously unexplored modulator for Ti-MOF synthesis. *In situ* NMR investigations show that phenol forms transient Ti-phenolate species in solution, regulating the high reactivity of Ti­(IV) precursors by reversible coordination of Ti centers, enabling controlled framework crystallization.

Extending this approach to different Ti-MOF systems reveals that its effectiveness is not universal but strongly depends on the underlying coordination chemistry, with the most pronounced improvements observed for catecholate-based frameworks. These results highlight the importance of matching modulator functionality with the coordination chemistry of the targeted framework to establish competitive and reversible coordination for strongly coordinating linker systems as a broader design principle for the crystallization and discovery of new MOFs based on strongly coordinating linkers.

This strategy enabled access to TUW-11, a novel and sustainable titanium ellagate MOF composed of isolated Ti­(IV) centers interconnected by ellagate ligands, forming a layered microporous framework. Although TUW-11 exhibits extended visible-light absorption and a notable photoresponse, its linker-centered photochemistry limits exciton separation and results in short-lived excited states, contrasting with many carboxylate-based Ti-MOFs, in which photoexcitation involves charge transfer to the metal centers. Nevertheless, by selecting substrate molecules that strongly adsorb to the framework, this linker-centered photoresponse can be leveraged for efficient light-assisted removal of organic molecules. The same strong adsorption, however, limits recyclability, highlighting a current challenge for photocatalytic applications of TUW-11. Overall, this work expands the synthetic toolbox for challenging Ti-MOFs while providing new insights into how strong metal-linker coordination can be controlled, and how this same strong coordination, while desirable for framework formation and stability, can also limit photophysical performance.

## Supplementary Material



## Notes

Synthetic and experimental details; materials and methods specifics; computational details; additional physical characterization; and additional supporting tables and figures (PDF)

## $

Departamento de Química Inorgánica, Universidad de Granada, Avenida Fuentenueva S/N, 18071 Granada, Spain
