# Supplementary material for: Phenol-Driven Modulation Enables the Crystallization of Titanium Metal–Organic Frameworks
Source: J Am Chem Soc. 2026 Oct 29;148(44):48257–67. doi: 10.1021/jacs.6c16169 (PMC13761203; doi:10.1021/jacs.6c16169)
Supplement: Supplementary file 1 [file ja6c16169_si_001.pdf]

Phenol-Driven Modulation Enables the Crystallization of Titanium Metal-Organic Frameworks

Pablo Ayala[a], Yan Zheng[b], Yu Xia[b], Cornelia von Baeckmann[a], Zheao Huang[a], Magdalena Ladisich[a], Sarah Hermetinger[a], Dogukan Apaydin[a], Alexey Cherevan[a], Guillermo Gómez-Tenés[d], Max Schmallegger[e], Georg Gescheidt[e], Freddy Kleitz[f], Maria Asghar[g], Florian Libisch[g], Natalia M. Padial[d], A. Ken Inge[b,c], Carlos Martí-Gastaldo[d], Tom Willhammar[b,c] and Dominik Eder[*a]

a Institute of Materials Chemistry
Technische Universität Wien
Getreidemarkt 9, 1060 Vienna, Austria
E-mail: dominik.eder@tuwien.ac.at

b Department of Chemistry
Stockholm University
Frescativägen 8, 106 91 Stockholm, Sweden

c Wallenberg Initiative Materials Science for Sustainability
Bredgatan 33, 602 21 Norrköping, Sweden

d Instituto de Ciencia Molecular (ICMol),
Universitat de València
Catedrático José Beltrán 2, Paterna 46980, Spain

e Institute of Physical and Theoretical Chemistry
Graz University of Technology
Stremayrgasse 9/I, 8010 Graz, Austria

f Department of Functional Materials and Catalysis, Faculty of Chemistry
University of Vienna
Währinger Straße 42, 1090 Vienna, Austria

g Institute of Theoretical Physics
Technische Universität Wien
Wiedner Hauptstraße 8-10/136, 1040 Vienna, Austria

Table of Contents

S1. General considerations 4
1.1. Materials 4
1.2. Methods 4
1.3. Routine Characterization 7
S2. Synthesis and upscaling of TUW-11 9
2.1. Synthesis of reference Ti-MOF materials 9
S3. TUW-11 synthesis optimization 10
3.1. Metal to linker ratio 10
3.2. Modulator type 11
3.3. Resulting particles, scanning electron microscopy (SEM) images 13
3.4. Modulator amount 16
3.5. Solvent 16
3.6. Temperature 17
3.7. Reaction time 17
3.8. Titanium precursor 18
3.9. Concentration 19
3.10. Heating rate 19
S4. Modulation 20
4.1. Retention of Ti in liquid phase 21
4.2. Competitive coordination titration followed by NMR 22
4.3. Phenol-assisted synthesis of other Ti-MOF materials 27
S5. Further characterization 31
5.1. Optical microscopy 31
5.2. Continuous rotation electron diffraction (cRED) 31
5.3. Scanning transmission electron microscopy (STEM) 32
5.4. Synchrotron high resolution powder X-ray diffraction (SPXRD) 33
5.5. X-Ray photoelectron Spectroscopy (XPS) 34
5.6. Extended X-ray absorption fine structure (EXAFS) 34
5.7. Fourier-transform infrared and Raman spectroscopy (FTIR and Raman) 37
5.8. ^{1}H-NMR of digested TUW-11 38
5.9. Solvent exchange 39
5.10. Thermogravimetric analysis (TGA) 39
5.11. Cation exchange 40
5.12. pH stability 41

5.13. Physisorption 43
5.14. UV-vis absorption of reference materials 44
5.15. Electrochemistry 46
5.16. Electron paramagnetic resonance (EPR) 49
5.17. Photoluminescence (PL) and time-resolved emission spectroscopy (TRES) 50
5.18. Density of states (DOS) 51
5.18. Point of zero charge (PZC) and zeta potential 52
S6. Selective adsorption and linker-centered photoconversion 53
6.1. Cycling and post-conversion characterization of TUW-11 57
S7. References 59

S1. General considerations

1.1. Materials

Titanium (IV) isopropoxide (97 %), acetic acid (99.7 %), methanol (99.9 %), ethanol (99 %), isopropanol (99.8 %), dimethylsulfoxide (p.a.), tetrahydrofuran (99%), phenol (p.a.), oxalic acid (95 %), methylene blue (p.a.) and tetrabutylammonium hexafluorophosphate (98 %) were purchased from Sigma-Aldrich. Ellagic acid (97 %), N,N-dimethylformamide (extra dry, 99.8 %), N,N-Diethylformamid (99 %) and catechol (99 %) were purchased from Thermo Scientific. Lactic acid chelated titanate, ammonium salt (Tyzor, 50 % in water), titanium (IV) n-butoxide (97 %), potassium chloride (99 %) were purchased from ABCR. Acetone (99.5 %) and acetonitrile (99.9 %) were purchased from VWR. Methyl orange (p.a.) was purchased from Fluorochem. All chemicals were used as purchased without any purification.

1.2. Methods

X-ray characterization techniques

Powder X-ray diffraction (PXRD) for optimization screening was recorded using a PANalytical XPert Pro diffractometer. The samples were loaded onto Si sample holders (1 x 1 cm). Diffractograms were acquired in Bragg-Brentano geometry (over a 4-50° 2θ range) with a Cu anode irradiation source with an energy of 8.04 keV (λ = 1.5406 Å). Measured intensities were collected using a semiconductor X'Celerator detector. Data below 5° 2θ are omitted due to an instrumental artifact occurring at low angles (2θ = 4-5°).

Capillary PXRD was recorded using a PANalytical Empyrean 2 diffractometer. The powder samples were filled into a 1.0 mm capillary (either with or without the solvent of choice), the capillary was sealed with wax, immobilized on a capillary spinner and aligned with a goniometer head. Diffractograms were acquired in transmission geometry (over a 2-50° 2θ range) with a Cu anode irradiation source with an energy of 8.04 keV (λ = 1.5406 Å). Measured intensities were collected using a GaliPIX3D detector.

Synchrotron high-resolution powder X-ray diffraction (HR-PXRD) was performed at beamline ID31 at the European Synchrotron Radiation Facility (ESRF). The sample powder was loaded into a cylindrical slot (approx. 1 mm thickness) and held between Kapton windows in a high-throughput sample holder. Diffractograms were acquired in transmission geometry with an incident X-ray energy of 75.051 keV (λ = 0.16520 Å). Measured intensities were collected using a Pilatus CdTe 2M detector (1679 x 1475 pixels, 172 x 172 μm^2 each) positioned with the incident beam in the corner of the detector (sample-to-detector distance ca. 1.5 m). Background measurements for the empty windows were measured and subtracted.

X-ray fluorescence spectroscopy (XRF) was performed with an Atomika 8030C X-ray fluorescence analyzer in total reflection geometry (T-XRF) for quantitative elemental analysis of metals. The X-ray source was the molybdenum monochromatized Kα-line with an energy of 17.5 keV. The excitation conditions were 50 kV and 47 mA for 100 s irradiation time and an energy-dispersive Si(Li)-detector was employed for intensity collection. Sample preparation solids: 1 mg of the MOF powder was loaded onto a clean quartz reflector, sealed with 5 µL of a 1 % PVA solution and dried for 5 min on a hot plate. Relative ratios of Ti to K were quantified. Sample preparation liquids: after the modulation experiments, the phases were separated through centrifugation and 990 µL of the liquid phase were mixed with 10 µL of a 1000 ppm Co internal standard solution, resulting in a total volume of 1 mL and c(Co) = 10 ppm. 5 µL of this solution were drop casted onto clean quartz reflectors. Ti was then quantified with respect to the internal standard.

XANES/EXAFS: Ti K-edge X-ray absorption fine spectroscopy (XAFS) measurements were performed at the BL17B1 beamline of the Shanghai Synchrotron Radiation Facility (SSRF). The beamline operates with a storage ring energy of 3.5 GeV and a maximum beam current of 250 mA. BL17B1 utilizes a bending-magnet source that provides a tunable photon energy range from 5 to 20 keV, suitable for probing the Ti K-edge region with high signal stability and energy resolution. The X-ray absorption near-edge structure (XANES) and extended X-ray absorption fine structure (EXAFS) spectra were collected in transmission mode at room temperature. Energy calibration was performed using a Ti metal foil as the reference. The acquired EXAFS data were processed using standard procedures, including pre-edge background subtraction, normalization to the edge jump, and extraction of $\chi(k)$ functions. Fourier transformation of the k^3-weighted $\chi(k)$ data yielded the radial structure functions (FT-EXAFS), which provide information on the local coordination environment around Ti atoms.

The X-ray photoelectron spectroscopy (XPS) was acquired using a custom-built SPECS XPS-spectrometer equipped with a monochromatised Al-Kα X-ray source (µFocus 350) and a hemispherical WAL-150 analyser. The powder was mounted onto the sample holder using double-sided Cu/carbon tape. The conditions were: excitation energy, 1486.6 eV; beam energy and spot size, 70 W onto 400 µm; angle, 51° to sample surface normal; base pressure, $5x10^{-10}$ mbar; pressure during measurements, 2×10^{-9} mbar.

<u>3D electron diffraction and imaging</u>

Three-dimensional electron diffraction data were obtained using the continuous rotation electron diffraction (cRED) technique, and data were collected on a JEOL JEM-2100 transmission electron microscope operated at 200 kV and equipped with a Timepix (ASI) detector. The data acquisition was carried out using the Instamatic software,[1] and the 3D reciprocal lattice was reconstructed with the REDp software.[2]

Scanning electron microscopy (SEM) imaging was done on a FEI QUANTA 250 FEG with an acceleration voltage of 2-5 kV typically, at a working distance of 10 mm. Samples were prepared by drop-casting onto a Si wafer (4 mm^2) and drying under ambient atmosphere.

Scanning transmission electron microscopy (STEM) images were obtained using a double aberration-corrected Thermo Fisher Scientific Themis Z operated at an acceleration voltage of 300 kV. The probe was converged with a semi-angle of 15 mrad, and the beam current was set at 1.8 pA. Images were obtained with the pixel size of 0.0372 nm or 0.0525 nm at a dwell time of 5 µs. iDPC STEM images were acquired using a 4-quadrant DF4 detector equipped using a collection angle of was 6 - 24 mrad at a camera length of 228 mm. iDPC images were reconstructed using custom-made Python code. For enhancing structural details, a high-pass filter was utilized to reduce the low-frequency components in the reconstruction of iDPC images.

<u>Gas physisorption</u>

The activation of the samples was performed as follows: acetone-exchanged material (3 x 4 mL) was immersed in a Tousimis Autosamdri-931 supercritical CO_2 activator chamber filled with acetone. The samples were exchanged several times with liquid CO_2 before heating and reaching the supercritical phase of CO_2. The supercritical point was maintained for 2 minutes. Finally, a slow venting of the chamber (90 min) was performed. The dry samples were transferred to the adsorption tubes, which were degassed at room temperature under 10^{-6} Torr for 30 minutes before measuring the isotherms.

Gas adsorption-desorption measurements: CO_2 sorption isotherms at 195 K were recorded using a Micromeritics 3Flex apparatus. N_2 sorption isotherms at 77 K, H_2O sorption isotherms at 298 K and

CO_2 isotherms at 298 K were collected on a Belsorp Max II. Prior to analysis, samples were desolvated using a Tousimis Autosamdri-931 supercritical CO_2 activator as described above. Surface area and pore volume values were determined from CO_2 adsorption-desorption isotherms at 195 K. The specific surface area (SA) was calculated using the multipoint Brunauer-Emmett-Teller (BET) method over the relative pressure range P/P_0 = 0.00-0.26 and pore volume was calculated using the single point adsorption method at P/P_0 = 0.96.

Electrode preparation

The working electrodes were prepared by drop casting 30 µL of a 5 mg mL^{-1} suspension of TUW-11 in DMSO onto fluorine-doped tin oxide (FTO) glass slides that were previously thoroughly cleaned. The covered surface was limited to 1.5 cm^2 aided by Kapton tape. The solvent was then evaporated on a hot plate at 60 °C.

Electrochemical Measurements

(Photo)electrochemical measurements were performed aided by potentiostats (Autolab PGSTAT 302N and PalmSens4, EmStat4) in a three-electrode configuration.

The TUW-11 coated FTO working electrodes were employed in cyclic voltammetry (CV) measurements for determining the HOCO and LUCO energy levels as well as the band gap. For this purpose, a three-electrode system was employed in which TUW-11 coated FTO electrodes served as the working electrode while an Ag/AgCl wire was used as the quasi-reference electrode. Finally, a Pt mesh was used as the counter electrode. 0.1 M tetrabutylammonium hexafluorophosphate solution in acetonitrile was used as the supporting electrolyte. Ferrocene/ferrocenium redox couple was used as the internal standard. All reported potentials are versus normal hydrogen electrode (NHE).

From the CV curves shown below we have calculated the highest occupied crystal orbital (HOCO) and lowest unoccupied crystal orbital (LUCO) according to the formulae below:

HOCO: $-(4.75 + E^{ox}_{\text{onset vs NHE}})$ eV LUCO: $-(4.75 + E^{red}_{\text{onset vs NHE}})$ eV

The onset potentials were determined using the linear part of the CV curves and are 1.08 V for oxidation and -0.45 V for the reduction which leads to a HOCO of -5.83 eV and LUCO of -4.30 eV. This delivers an electrochemical band gap of 1.54 eV.

Electron paramagnetic resonance (EPR) spectroscopy

Spectra were recorded on a Magnettech Minscope MS300 X-band EPR spectrometer. Irradiation of samples was performed using a Hamamatsu LC8 (Hg/Xe, λ_{max} = 365 nm) UV lamp outside the EPR spectrometer. EPR spectra indicating change of the oxidation state of Ti (Ti^{3+}/Ti^{4+}) upon irradiation were recorded at 77 K. Measurements were performed before and after 1 or 10 minutes of sample irradiation. ST-EPR sample solutions consisted of the MOF (2 mg mL^{-1} in H_2O) and the spin-trapping agent 5,5-dimethyl-1-pyrroline *N*-oxide (DMPO, 50 mM).

Dye adsorption and degradation experiments

Adsorption**:** 3 mg of as-synthesized MOF were dispersed in 15 mL of a 130 mg L^{-1} aqueous solution of methylene blue (MB) or methyl orange (MO) by sonicating for 1 min in 20 mL borosilicate glass vials (external diameter = 27 mm). The mixture was then stirred at 300 rpm for 4 hours (equilibration time). During this time, aliquots of the suspension were taken at different times to estimate the adsorption capacity.

Light-assisted removal: a high-throughput photoreactor (Photospinner, patent PCT/ES2024/070188, ES2986236) was used to ensure uniform irradiation and reproducibility[3]. After the equilibration time, the visible light LED lamps (4 cm away from the vials) with λ = 445 nm and I_{avr} = 0.435 W cm^{-2} were turned on (t = 0) and 1.5 mL aliquots of the suspension were taken at t = 0, 30, 60, 90 and 120 min. The catalyst was separated from the solution through syringe filtration (nylon filter, 0.45 µm). 1 mL of the filtrate was then analyzed with UV-vis spectroscopy to estimate the concentration of the respective dye in the solution, aided by calibration curves using known concentrations. The signals used for MB and MO were their characteristic peaks at 664 nm and 464 nm, respectively.

Computational methods

All electronic structure calculations presented in the manuscript were carried out using the all-electron FHI-aims package with the light species-default settings.[4] Geometry optimization was started from the idealized structure model of TUW-11, which was pre-optimized with a GGA functional (Perdew-Burke-Ernzerhof, PBE) and then with a hybrid functional to add a fraction of exact exchange (PBE0).[5] Given the large unit cell (148 atoms, 988 electrons), a Γ-point-only calculation proved sufficient. Atomic coordinates were relaxed until achieving a maximum force below 5 meV $Å^{-1}$. The lattice vectors were kept fixed during the relaxation. After structural relaxation, we sampled the density of states (DOS) and partial density of states (PDOS) with a 4x4x1K grid at the same level of theory. The band structure of TUW-11 was evaluated along the k path of Γ–M–K–Γ.

1.3. Routine Characterization

^{1}H and ^{13}C NMR spectra were recorded using a 400 MHz Avance NEO, Bruker Ascend spectrometer at 25 °C. Chemical shifts (δ) are reported in ppm. The resonance of residual DMSO for DMSO-d_6 (2.5 ppm) and of residual DMF for DMF-d_7 (8.2 ppm) is used as internal reference. For digestions, 5 mg of sample were digested in DMSO-d_6 (500 µL) and concentrated DCl (20 µL) and the resulting solutions were measured on a Bruker Avance spectrometer (250 MHz).

Attenuated-Total Reflection Fourier-Transformed Infrared Spectroscopy (ATR-FTIR) was recorded on a Bruker Tensor 27 spectrometer with a diamond crystal accessory in the solid state.

Raman spectra were recorded using a HORIBA LabRAM HR Evolution Raman microscope equipped with a 532 nm excitation laser, a high-sensitivity charge-coupled device (CCD) detector, and a long-working-distance objective lens. The spectrometer provides high spectral resolution and stable signal acquisition, enabling accurate characterization of vibrational features.

Thermogravimetric Analysis (TGA) was carried out with a Mettler Toledo TGA/SDTA 851 apparatus between 25 and 700 °C under ambient conditions (10 °C min^{-1} scan rate and an air flow of 30 mL min^{-1}).

Elemental Analysis (EA) of carbon, nitrogen, oxygen and hydrogen contents were determined by microanalytical procedures using a LECO CHNS.

Diffuse reflectance spectroscopy (DRS), i.e. UV-Vis spectroscopy in solid-state, was obtained with a Jasco 670 spectrometer. The powders were loaded onto a 2 mm sample holder and the reflectance profile was recorded in the range between 200 and 900 nm relative to a $MgSO_4$ reference. Absorbance (A) has been calculated from reflectance (R) values with: A = 1 $[\log (R/100)]^{-1}$. Using the Kubelka-Munk Theory, the Tauc functions are obtained, which allow to determine the optical band gap ($E_{g, opt.}$) of the material in question by extrapolating the linear regime of the plots to zero.[6–8]

Zeta-potential measurements were performed using a Malvern Zetasizer Nano ZS. Stable aqueous suspensions of the material in water (pH = 6.5; 1-5 mg mL^{-1}) were obtained by ultrasonic bath treatment. Prior to zeta-potential measurements, a standard solution with a zeta-potential of -42 ± 6 mV was measured to ensure correct calibration. Zeta-Potentials are reported as an average of 18 measurements, with each measurement consisting of 20-200 runs.

S2. Synthesis and upscaling of TUW-11

All operations for a common synthesis of TUW-11 were performed under argon atmosphere. 43.5 mg (0.14 mmol) EA and 1.12 mL of anhydrous DMF were charged into an 8 mL screw cap glass vial. 2.88 mL of a previously prepared 6M phenol solution (240 equivalents) in anhydrous DMF was added to the mixture, which was stirred and subsequently 21.2 µL (0.07 mmol) of TTIP were dropped onto the slurry. The vial was closed, stirred for further 5 min, and then heated in an oven for 48 h at 120 °C (heating and cooling rates were 1.67 and 0.28 °C min^{-1}, respectively). After cooling down to room temperature, the red microcrystalline product was centrifuged and washed with DMF (3x4 mL) and acetone (3 x 4 mL). The product was then dried at room temperature, typically resulting in a 73 % yield.

The synthesis can be scaled up 2.5- and 31-fold by multiplying the amounts of reagents by these factors and using a 20 mL screw cap glass vial or a 250 mL screw cap glass bottle, respectively. The solid is obtained in similar yields as above and shows the same XRD pattern, albeit a few differences in relative intensities can be observed for the 31-fold experiments, as shown in **Figure S1**. Synthesis optimization can be found in **Section 3.**

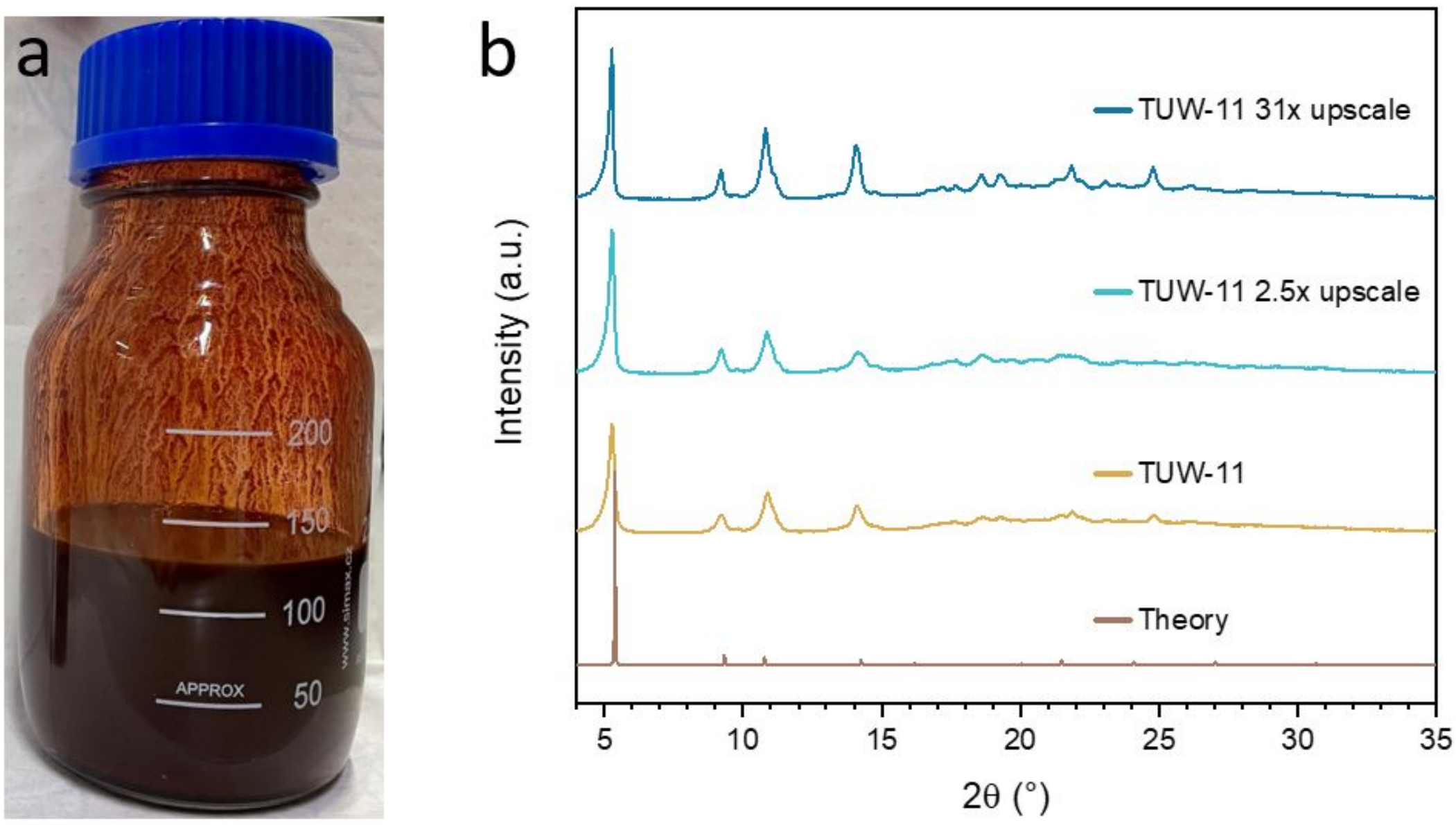

Figure S1. a) Digital image of the 31x upscaling experiment using a 250 mL screw cap bottle. **b)** Capillary PXRD patterns of the as-synthesized TUW-11 powders implementing different synthetic scales compared to the simulated pattern of TUW-11.

2.1. Synthesis of reference Ti-MOF materials

The reference Ti-MOFs were synthesized according to the methods described before by Nguyen et al.[9] for Ti-CAT-5; Salcedo et al.[10] for IEF-11; Yu et al.[11] for NTU-9; Padial et al.[12] for MUV-11; Yan et al.[13] for USTC-700; and Naghdi et al.[14] for MIL-125.

The adapted synthetic methods for showing the transferability of phenol-modulation can be found in **Section 4**.

S3. TUW-11 synthesis optimization

Optimization of TUW-11 was achieved by screening various reaction parameters, including metal to linker ratio, modulator type and amount, solvent, temperature, reaction time, and precursor type.

3.1. Metal to linker ratio

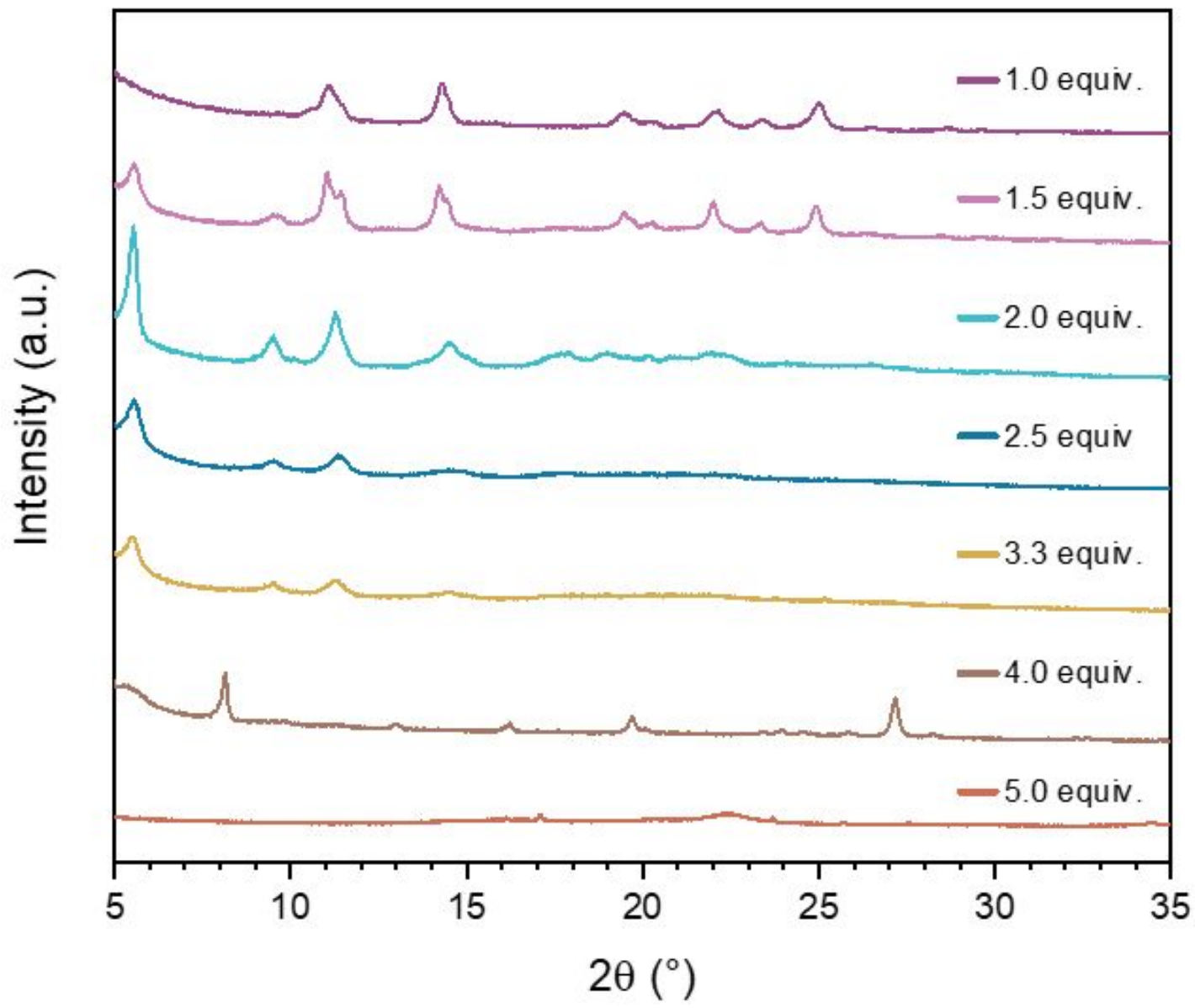

Figure S2. PXRD diffraction patterns (Bragg-Brentano) of the obtained powders resulting from varying metal to linker ratios (keeping metal = 1 equivalent). The metal precursor used was TTIP as the Ti(IV) source. Reaction conditions: DMF, 120 equiv. PhOH, 20 mM, 120 °C, 48 h. TUW-11 can be obtained in a range of 2-3 linker equivalents.

3.2. Modulator type

Modulator identity and equivalent were systematically varied during synthesis optimization, including lower equivalents for strongly coordinating chelating modulators. The coordination characteristics of the investigated modulators and representative synthetic outcomes are summarized in the table below.

Table S1. Chemical and coordination characteristics of the modulators investigated during TUW-11 synthesis optimization and their representative synthetic outcomes.

Modulator	Expected coordination	Coordinating group	*pKa_1	*pKa_2	Outcome
Methanol	Monodentate	$R\text{-}O^-$	15.5	-	Different phase
**Phenol**	**Monodentate**	**$R\text{-}O^-$**	**10.0**	**-**	**Highly crystalline TUW-11**
1-Naphthol	Monodentate	$R\text{-}O^-$	9.34	-	TUW-11
2-Naphthol	Monodentate	$R\text{-}O^-$	9.51	-	TUW-11
Acetic acid	Mono/bidentate	$R\text{-}COO^-$	4.76	-	Low crystallinity TUW-11
Catechol	Bidentate	2 x $R\text{-}O^-$	9.45	12.8	Different phase
Salicylic acid	Bidentate	$R\text{-}COO^-$ + $R\text{-}O^-$	2.97	13.6	No solid
Oxalic acid	Bidentate	2 x $R\text{-}COO^-$	1.25	3.75	Low crystallinity TUW-11
Squaric acid	Bidentate	2 x $R\text{-}O^-$	~0.6	3.48	Low crystallinity TUW-11

* pKa values refer to aqueous solution and are included as qualitative descriptors only, albeit modulator strength in DMF will be different. Values were taken from the IUPAC compilation of Serjeant and Demspey. [15,16]

Table S2. Full width at half maximum (FWHM) for the first three peaks of TUW-11 synthesized under optimized conditions with phenol, 1-naphthol and 2-naphthol (PXRD patterns in **Figure S4**).

Peak position 2θ (°)	Phenol FWHM (°)	1-Naphthol FWHM (°)	2-Naphthol FWHM (°)
5.2	0.22	0.33	0.25
9.1	0.23	0.37	0.46
10.7	0.33	0.50	0.75

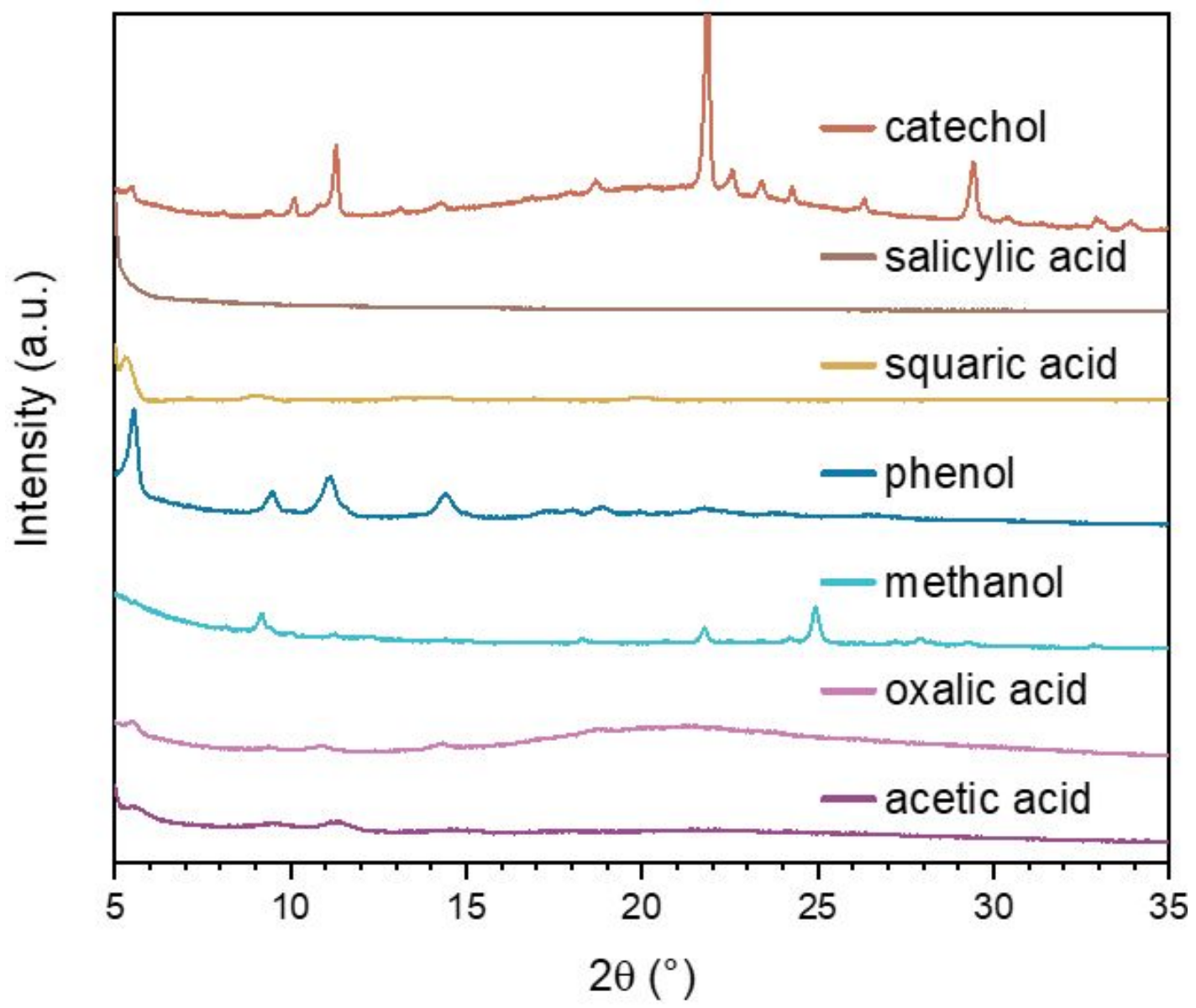

Figure S3. PXRD diffraction patterns (Bragg-Brentano) of the obtained powders resulting from varying the type of modulator (keeping modulator equivalent = 120). Reaction conditions: M:L ratio = 1:2, DMF, 20 mM, 120 °C, 48 h.

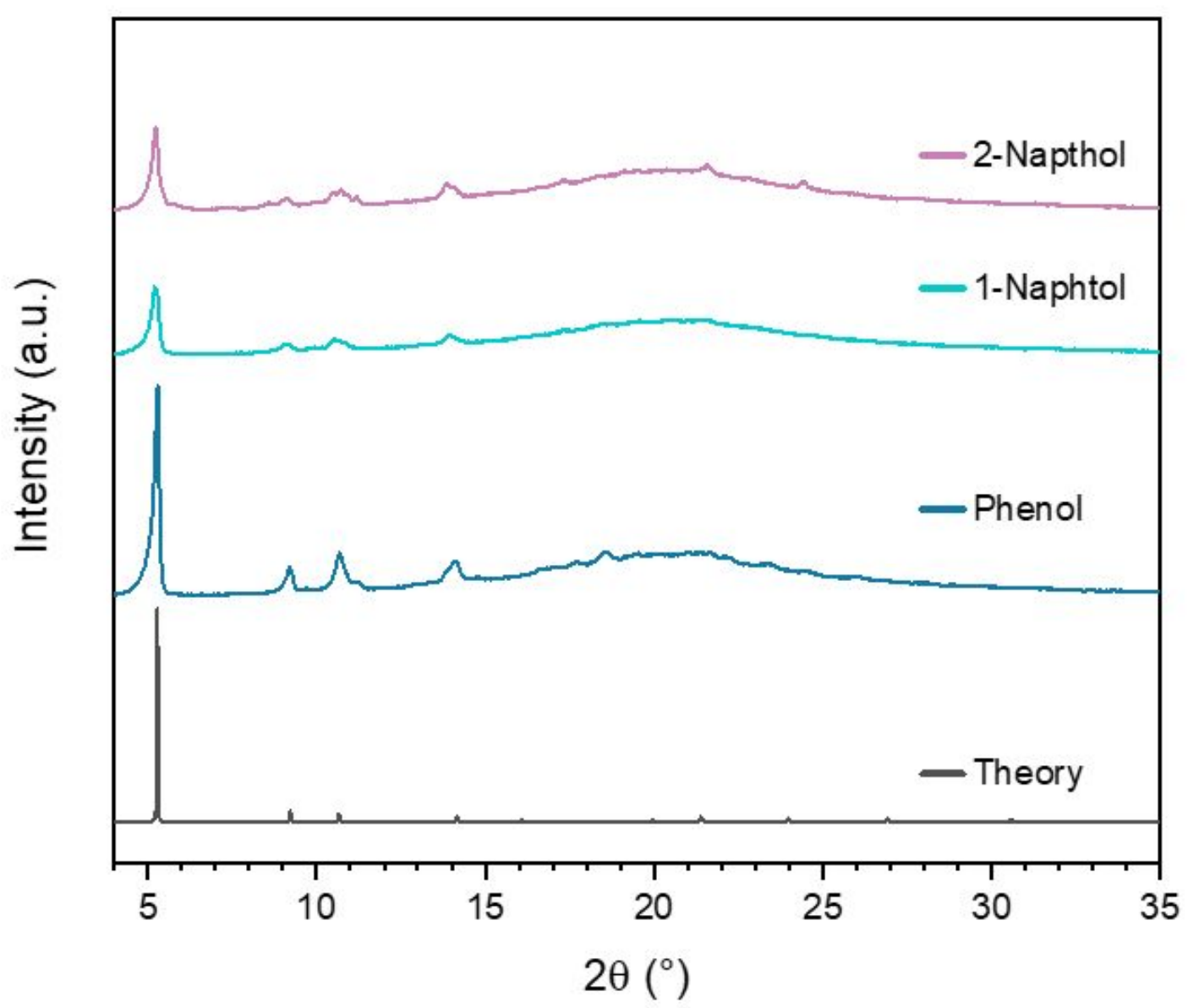

Figure S4. Capillary PXRD patterns of TUW-11 obtained using 1-naphthol and 2-naphthol as modulators under the optimized conditions (48 h, 120 °C, 120 equivalents). The phenol-modulated TUW-11 under the same conditions is shown for comparison.

3.3. Resulting particles, scanning electron microscopy (SEM) images

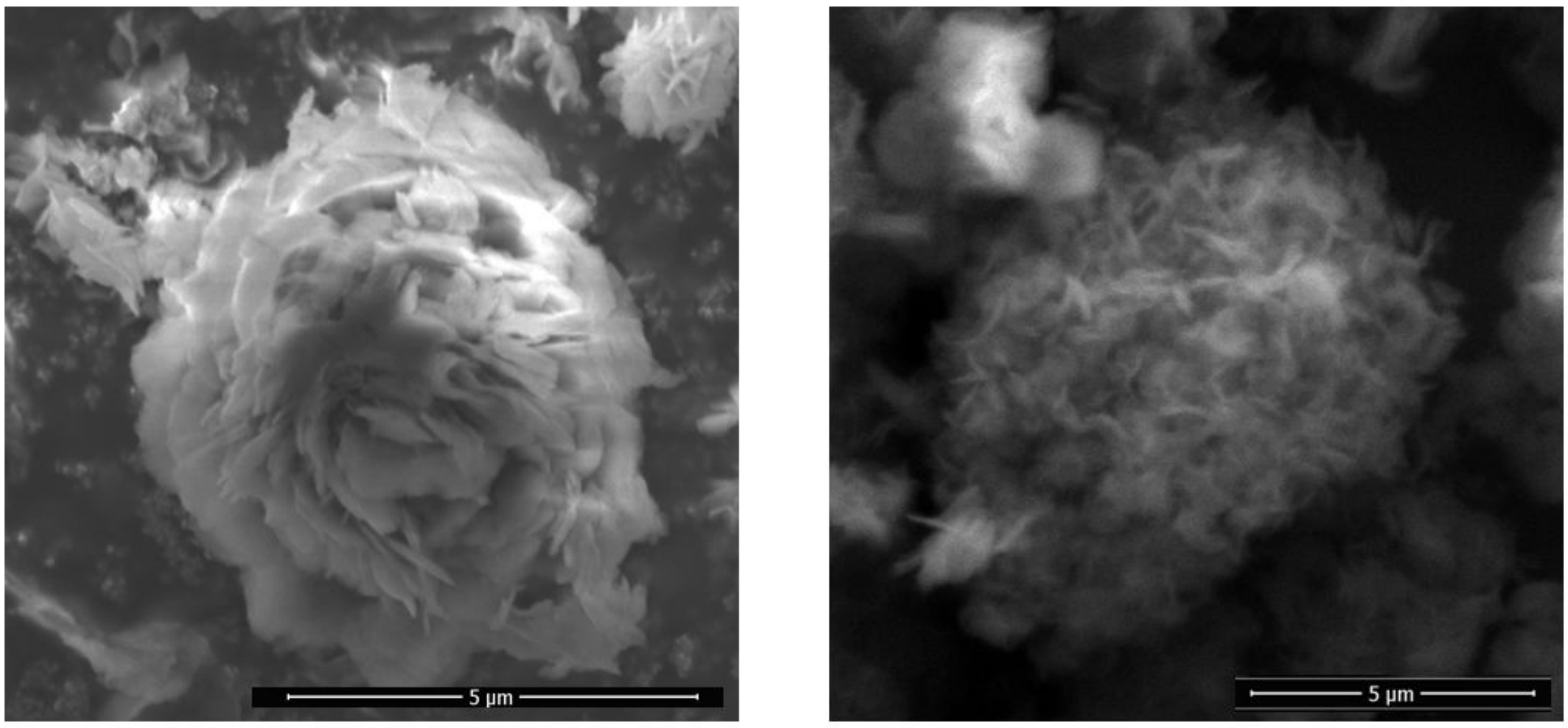

Figure S5. SEM of the modulated synthesis using 120 equiv. AA. Reaction conditions: M:L ratio = 1:2, DMF, 20 mM, 120 °C, 48 h. AA modulation results in small nanosheets of low crystallinity.

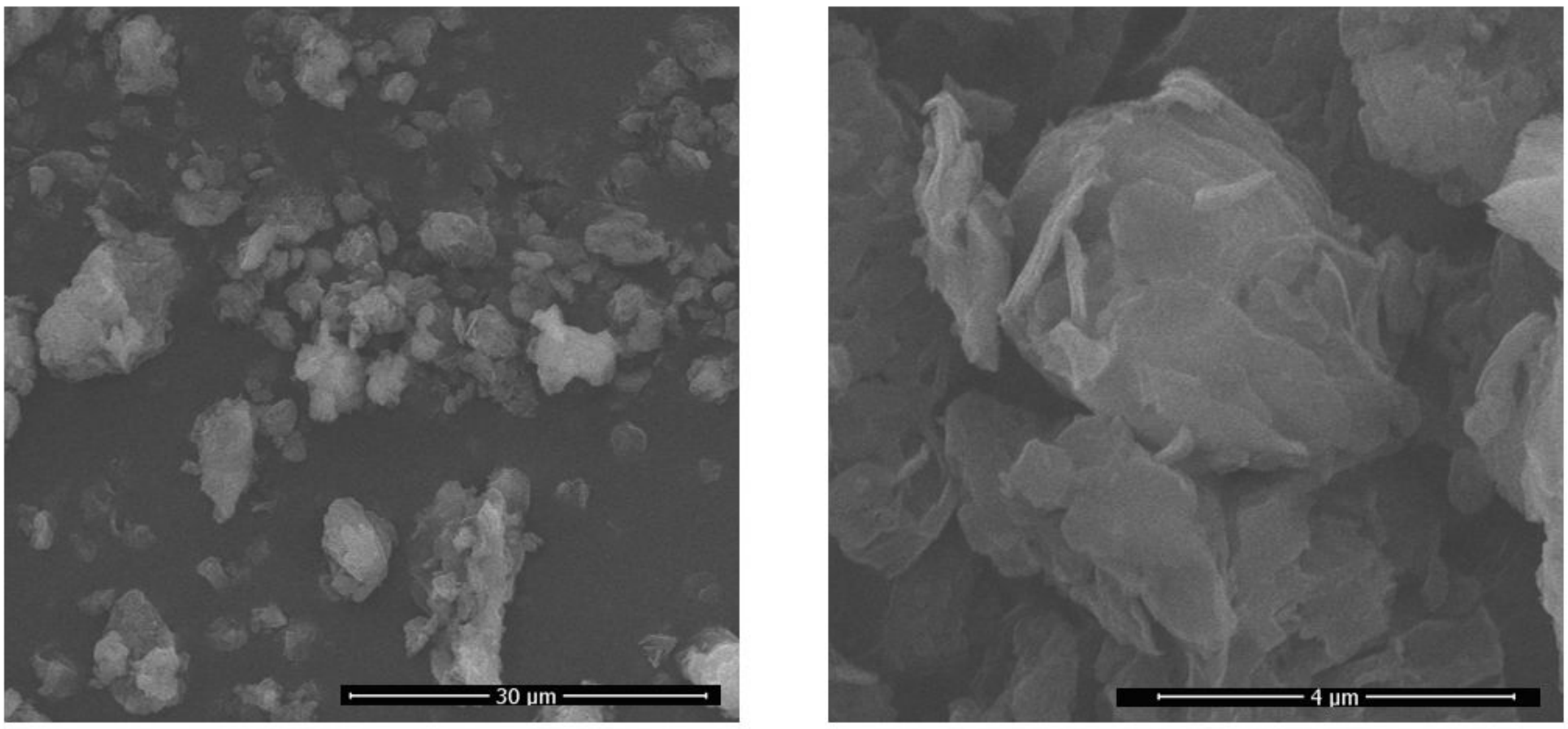

Figure S6. SEM of the modulated synthesis using 120 equiv. oxalic acid. Reaction conditions: M:L ratio = 1:2, DMF, 20 mM, 120 °C, 48 h.

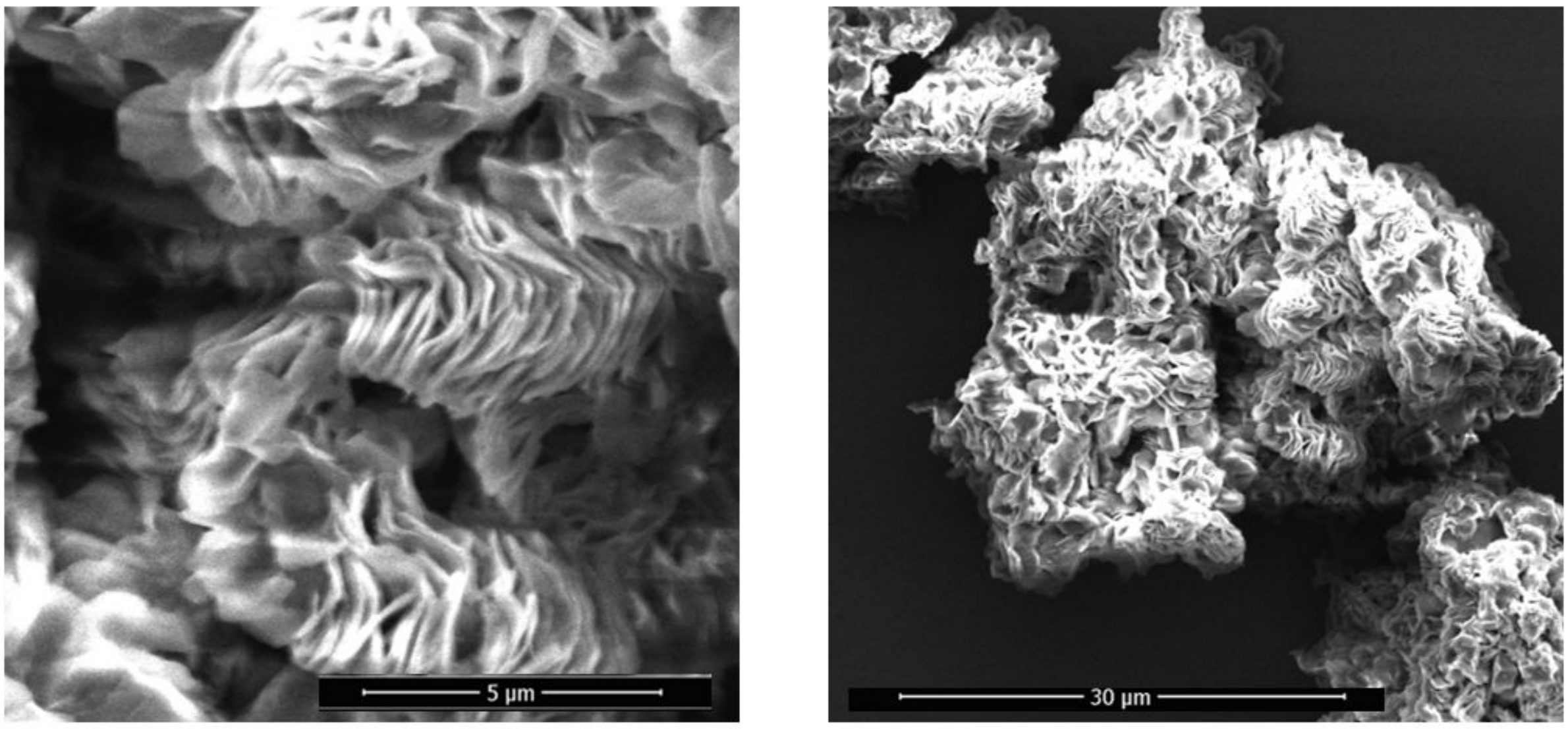

Figure S7. SEM of the modulated synthesis using 120 equiv. MeOH. Reaction conditions: M:L ratio = 1:2, DMF, 20 mM, 120 °C, 48 h.

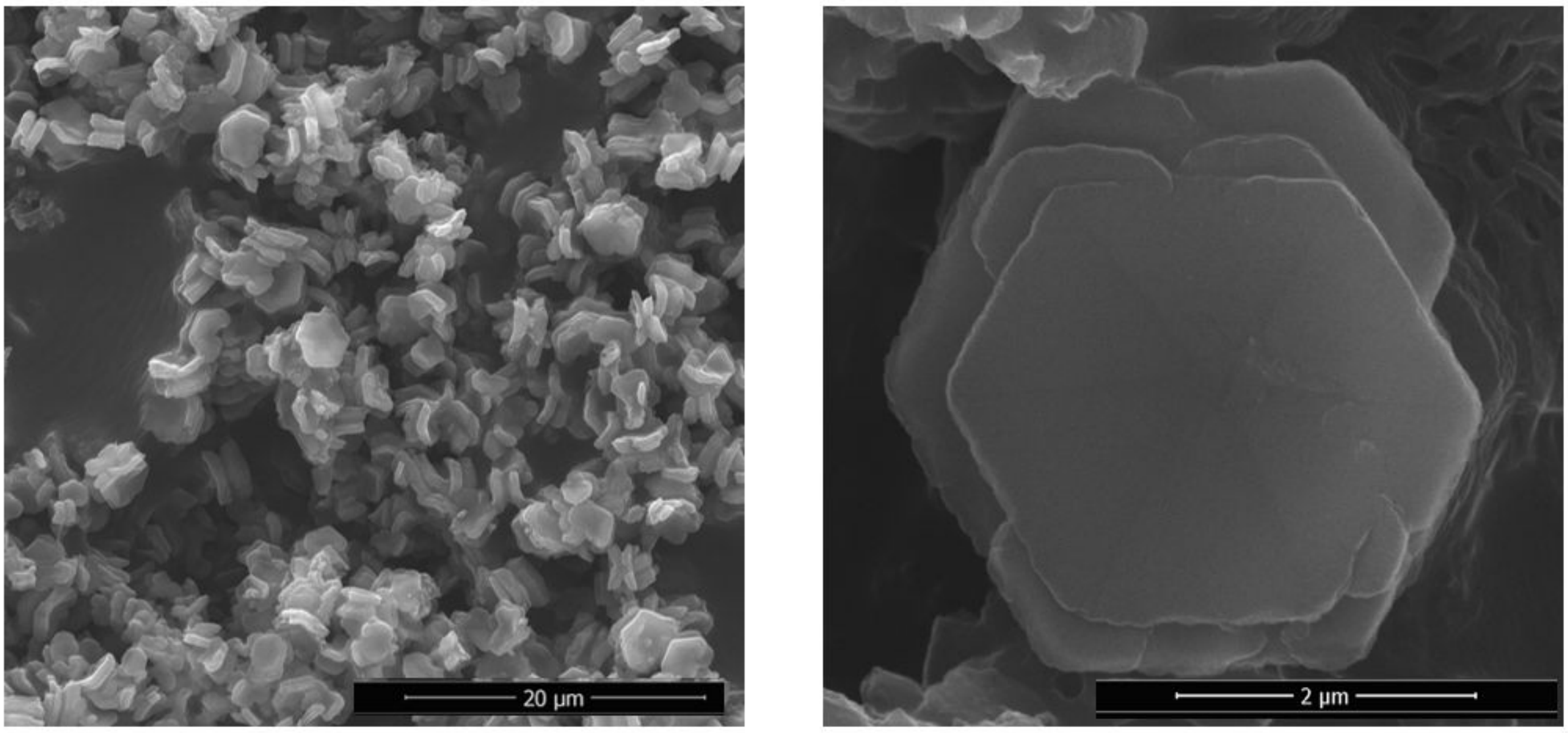

Figure S8. SEM of the modulated synthesis using 120 equiv. PhOH. Reaction conditions: M:L ratio = 1:2, DMF, 20 mM, 120 °C, 48 h.

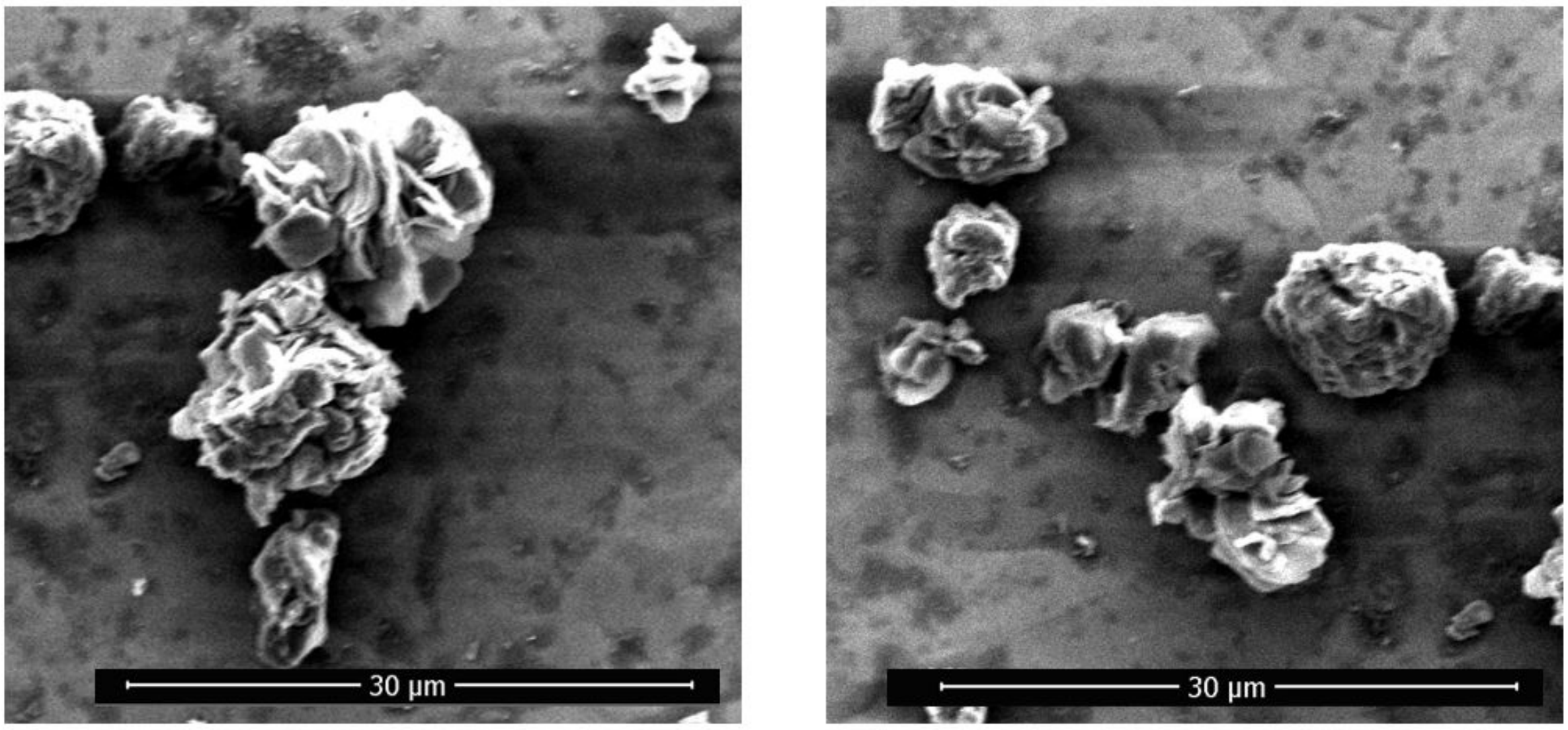

Figure S9. SEM of the modulated synthesis using 120 equiv. catechol. Reaction conditions: M:L ratio = 1:2, DMF, 20 mM, 120 °C, 48 h.

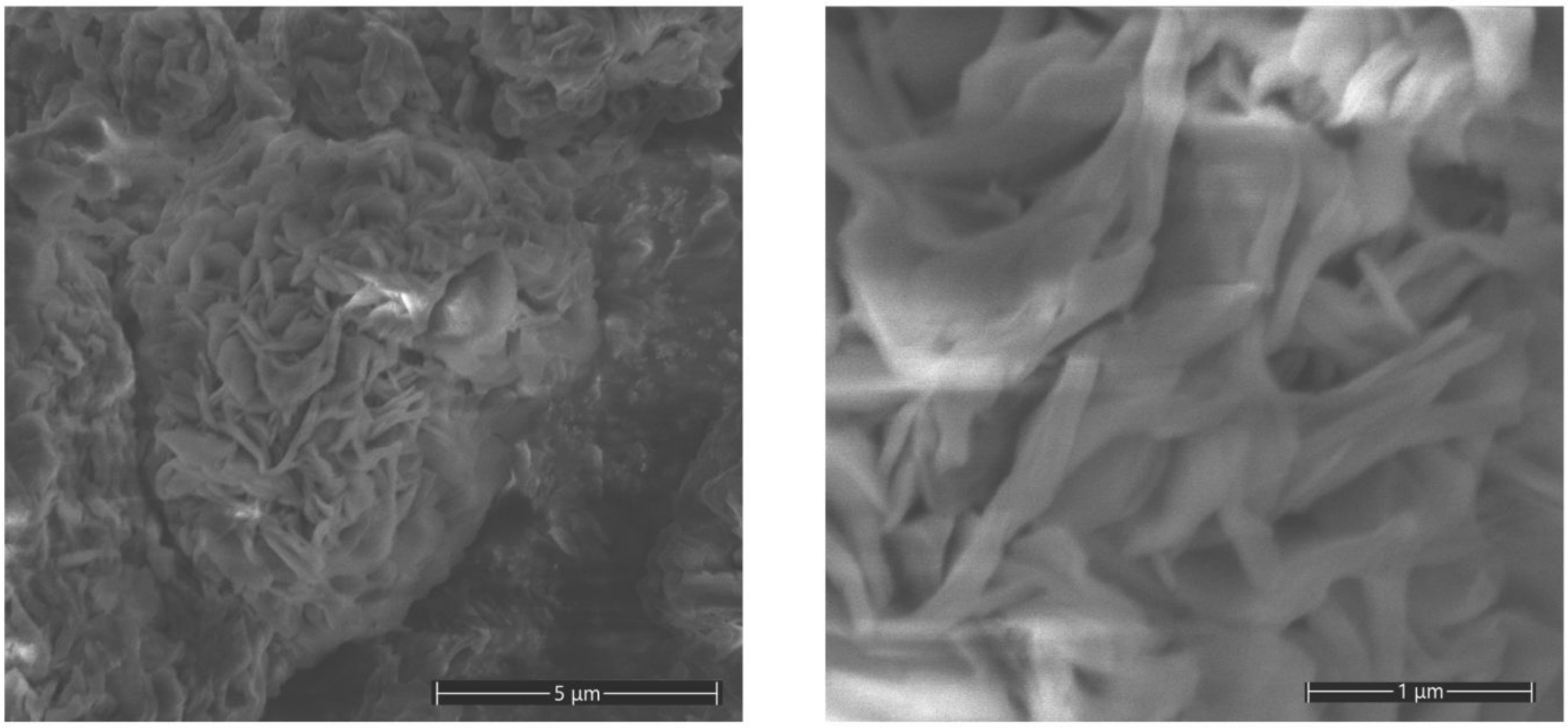

Figure S10. SEM of the modulated synthesis using 120 equiv. squaric acid. Reaction conditions: M:L ratio = 1:2, DMF, 20 mM, 120 °C, 48 h.

3.4. Modulator amount

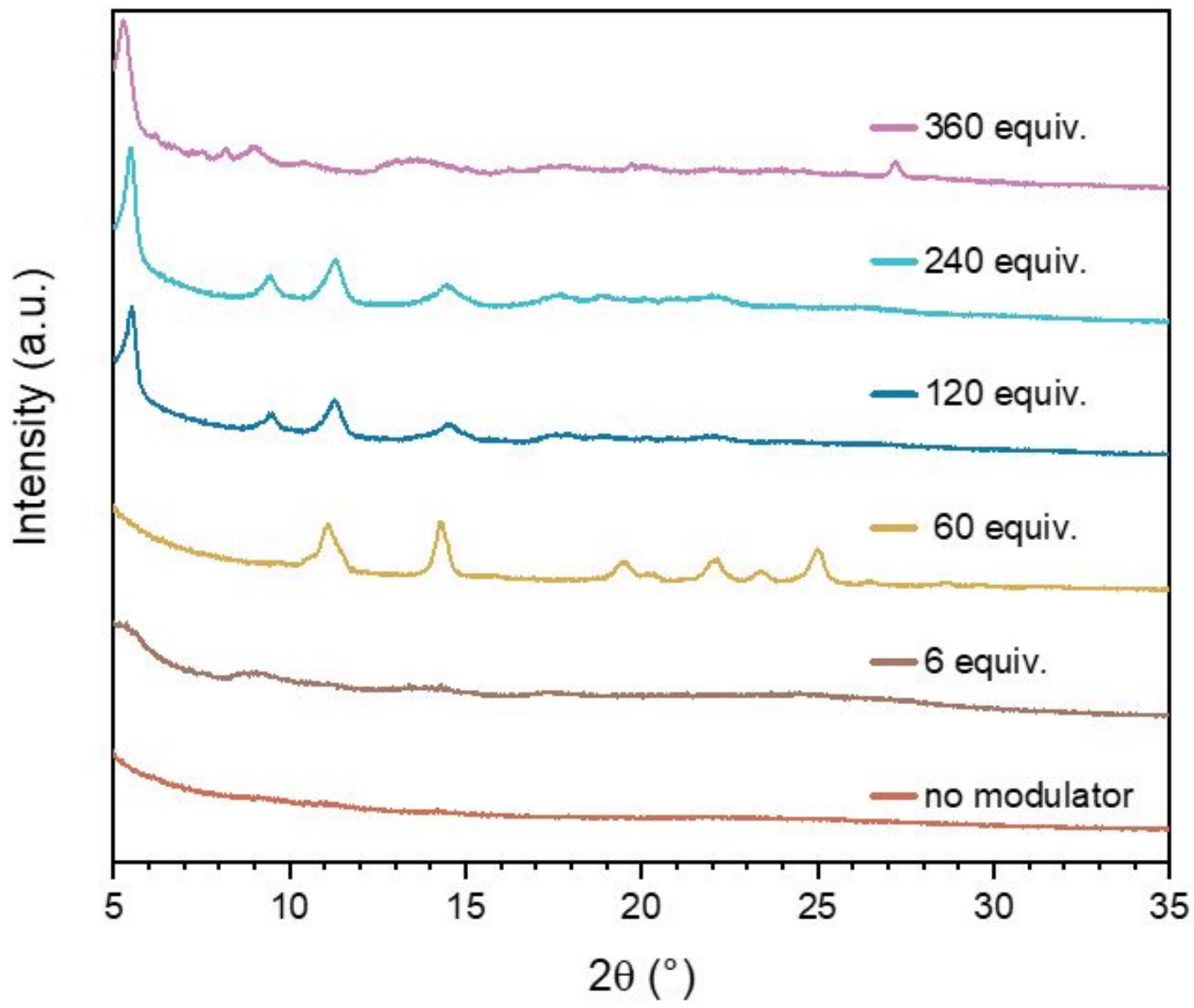

Figure S11. PXRD diffraction patterns (Bragg-Brentano) of the obtained powders resulting from varying the amount of phenol. Reaction conditions: M:L ratio = 1:2, DMF, 20 mM, 120 °C, 48 h.

3.5. Solvent

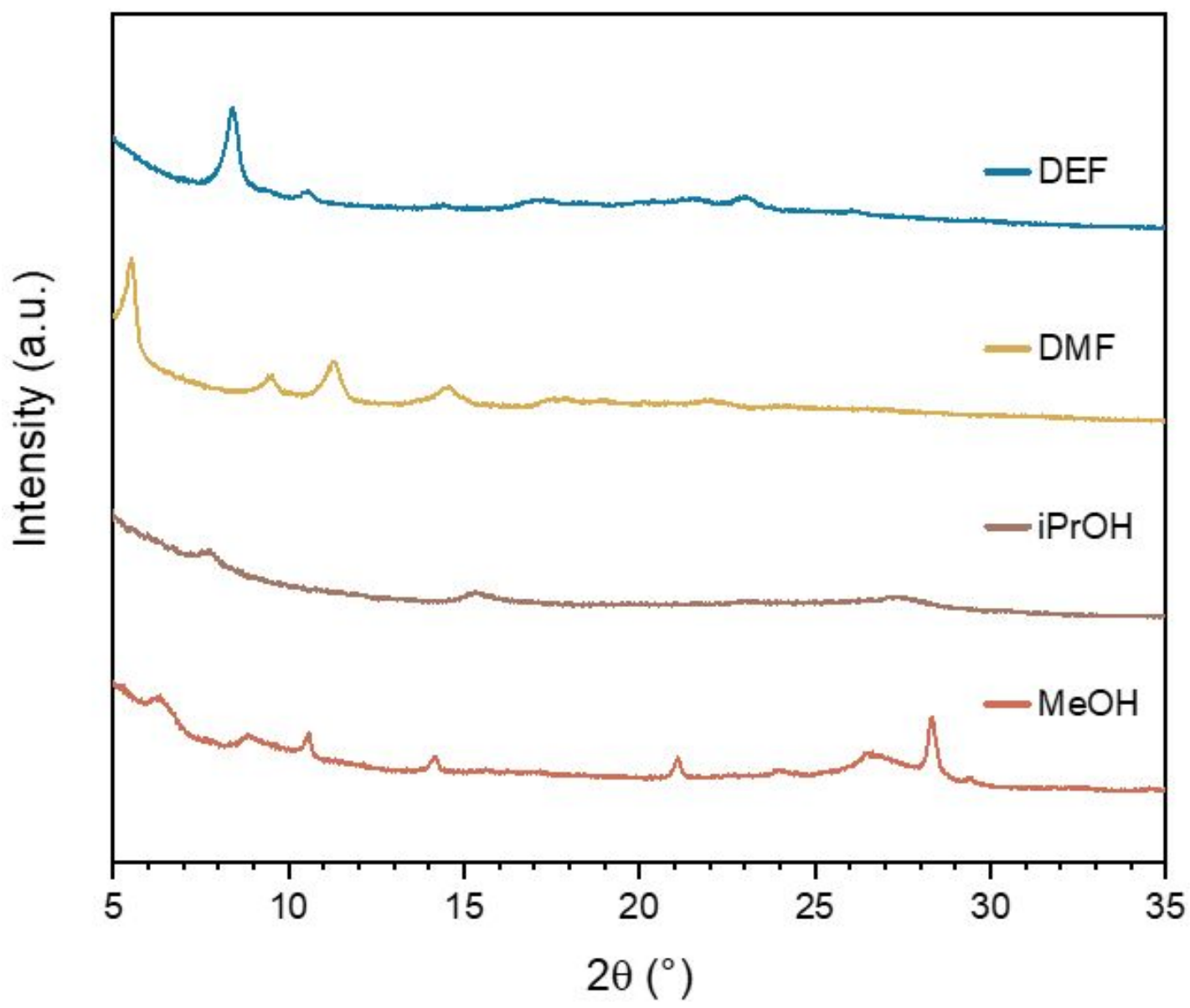

Figure S12. PXRD diffraction patterns (Bragg-Brentano) of the obtained powders resulting from varying the solvent. Reaction conditions: M:L ratio = 1:2, DMF, 20 mM, 120 equiv. PhOH, 120 °C, 48 h.

3.6. Temperature

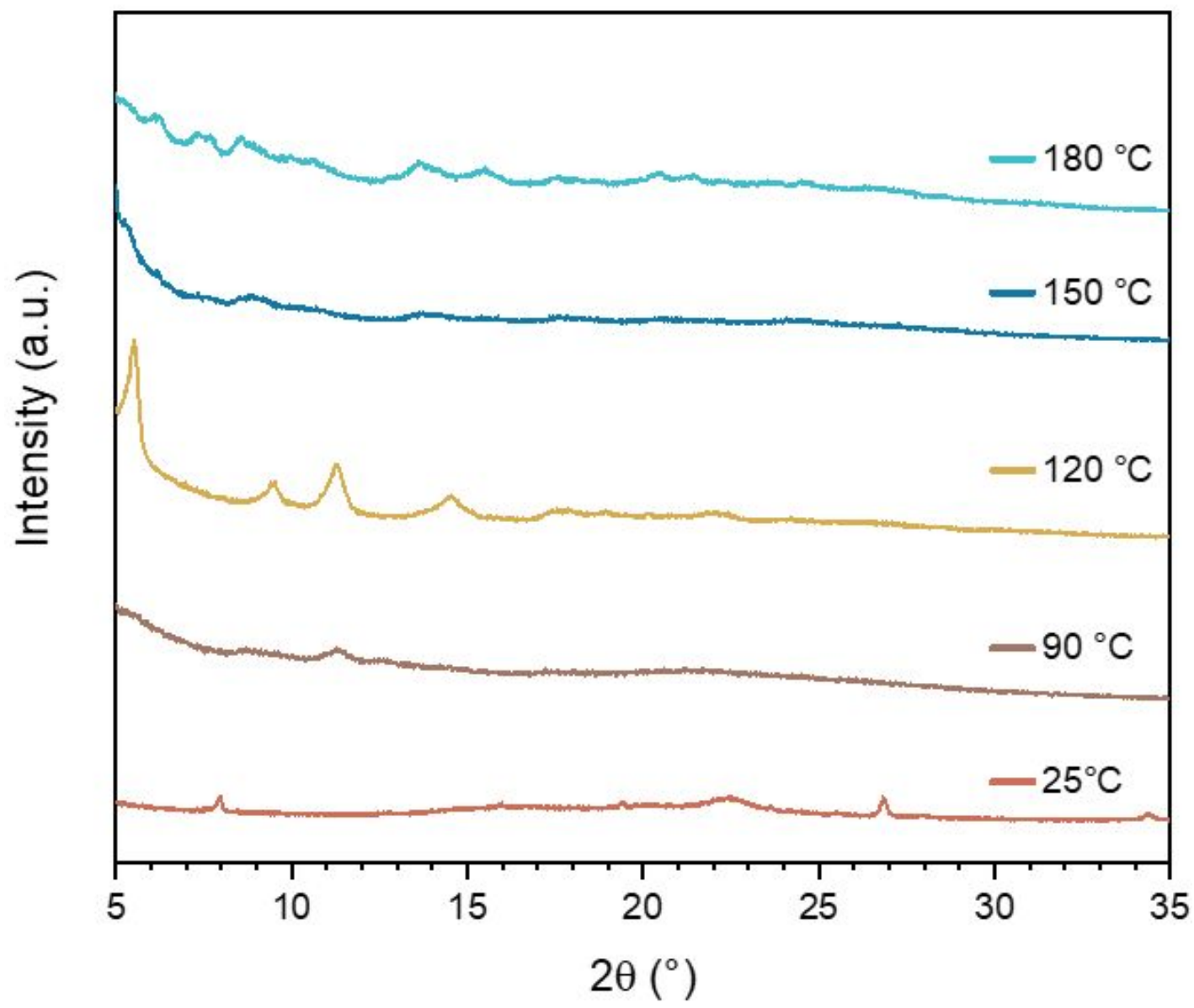

Figure S13. PXRD diffraction patterns (Bragg-Brentano) of the obtained powders resulting from varying the reaction temperature. Reaction conditions: M:L ratio = 1:2, DMF, 20 mM, 120 equiv. PhOH, 48 h.

3.7. Reaction time

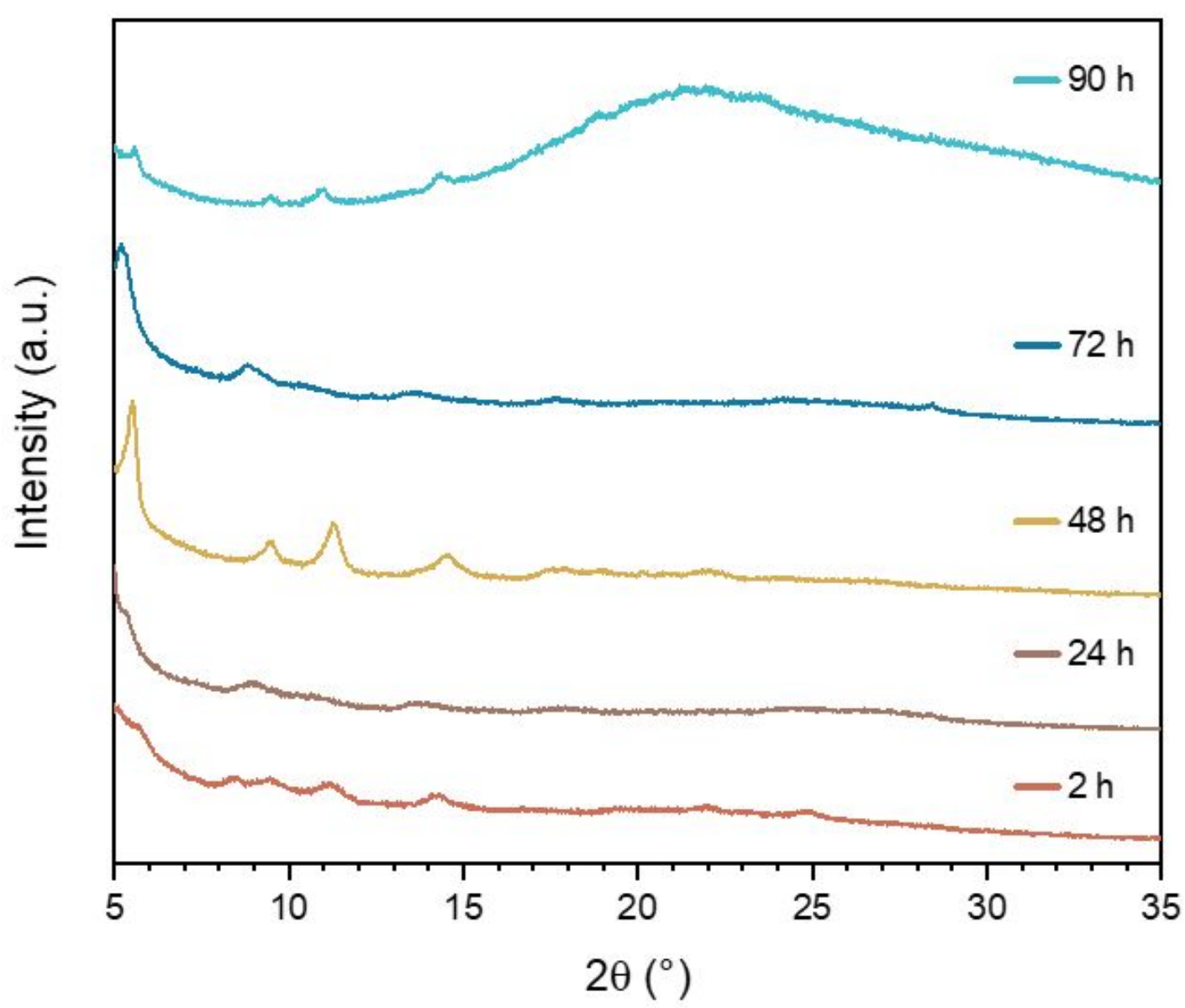

Figure S14. PXRD diffraction patterns (Bragg-Brentano) of the obtained powders resulting from varying the reaction time. Reaction conditions: M:L ratio = 1:2, DMF, 20 mM, 120 equiv. PhOH, 120 °C.

3.8. Titanium precursor

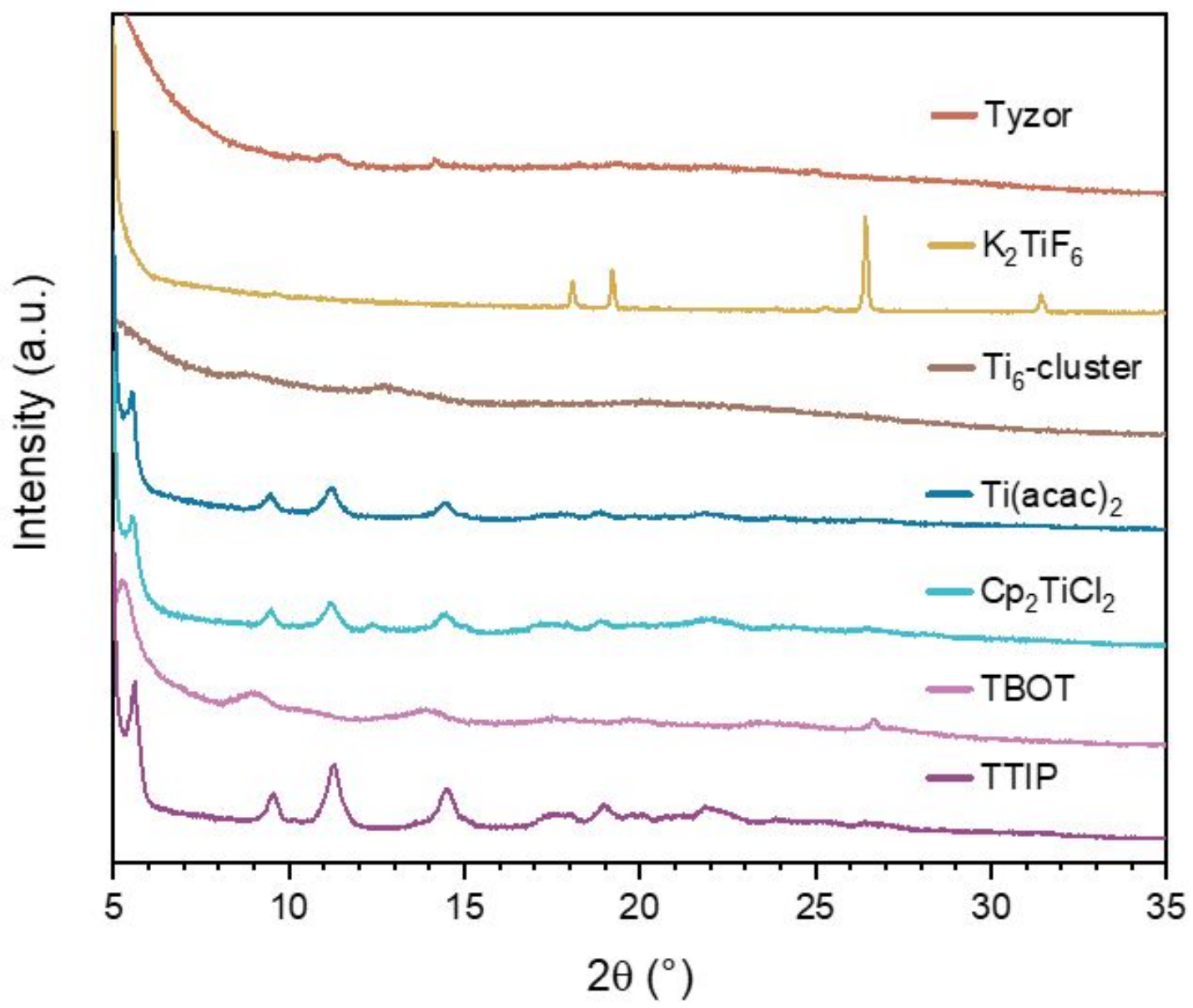

Figure S15. PXRD diffraction patterns (Bragg-Brentano) of the obtained powders resulting from varying the type of Ti(IV) precursor. Reaction conditions: M:L ratio = 1:2, DMF, 20 mM, 120 equiv. PhOH, 120 °C, 48 h.

3.9. Concentration

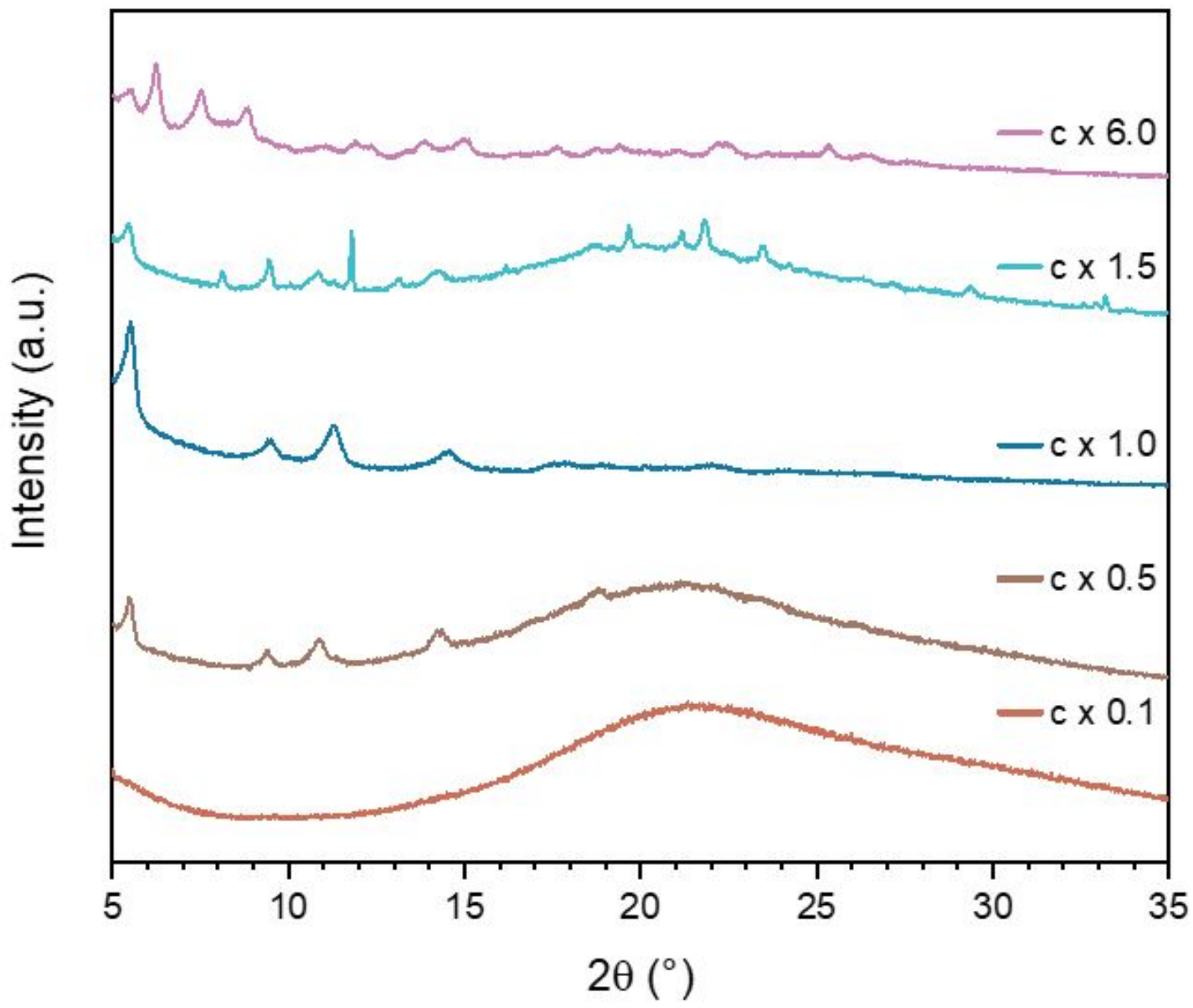

Figure S16. PXRD diffraction patterns (Bragg-Brentano) of the obtained powders resulting from varying the concentration of the Ti(IV) precursor. Reaction conditions: M:L ratio = 1:2, DMF, c x 1.0 = 20 mM, 120 equiv. PhOH, 120 °C, 48 h.

3.10. Heating rate

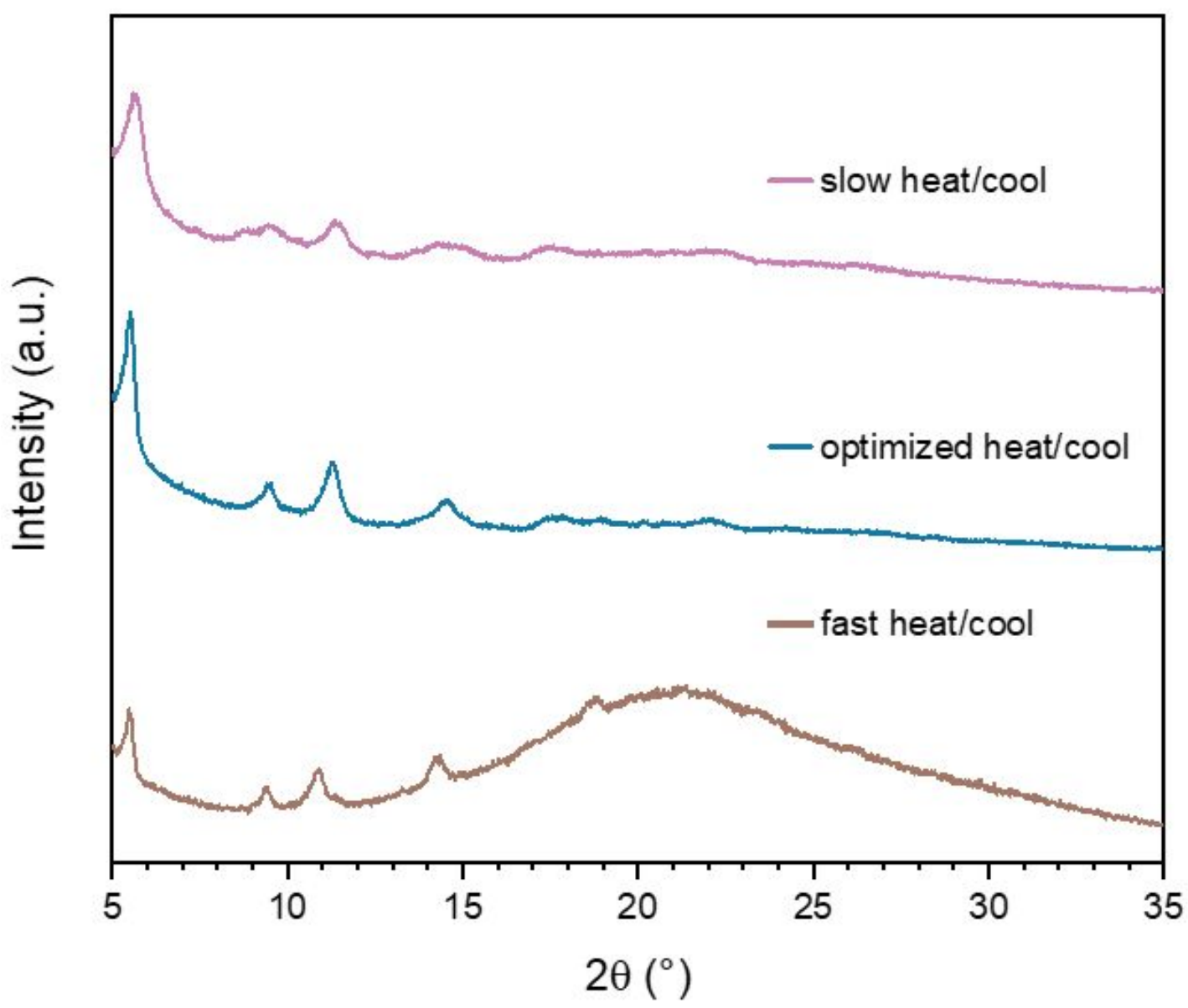

Figure S17. PXRD diffraction patterns (Bragg-Brentano) of the obtained powders resulting from varying the heating and cooling rates. Slow = heat in 24 h, keep T for 48 h, cool in 24 h. Optimized = heat in 1 h, keep T for 48 h, cool in 4 h. Fast = pre-heat oven, keep T for 48 h, cool immediately by water bath. Reaction conditions: M:L ratio = 1:2, DMF, 20 mM, 120 equiv. PhOH, 120 °C, 48 h.

S4. Modulation

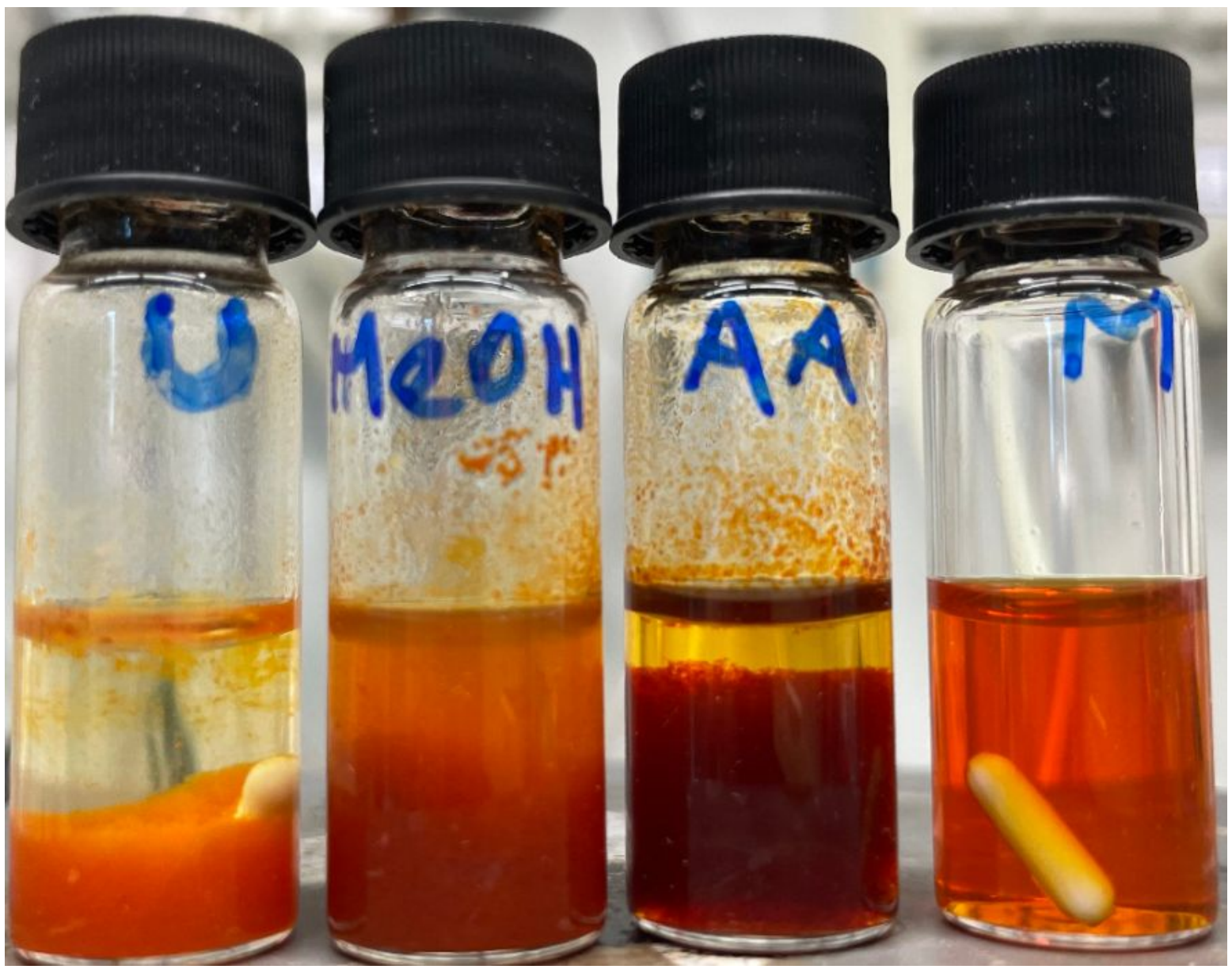

Figure S18. Digital image of the precipitation products after addition of TTIP (1 equiv.) to a solution of EA (c = 3.3 mM, 5 x less concentrated than in the standard synthesis of TUW-11) in DMF (2 mL), with 200 equiv. of different modulators. U: unmodulated (just TTIP and EA), MeOH: methanol, AA: acetic acid and M: modulated (PhOH). Instant precipitation in the case of U, MeOH and AA can be observed, while the modulated experiment stays in solution, without any precipitation of any amorphous solid. Similar results (no precipitation) were achieved with catechol or oxalic acid, however, the TUW-11 phase could not be isolated with these stronger modulators at the optimal synthetic conditions.

4.1. Retention of Ti in liquid phase

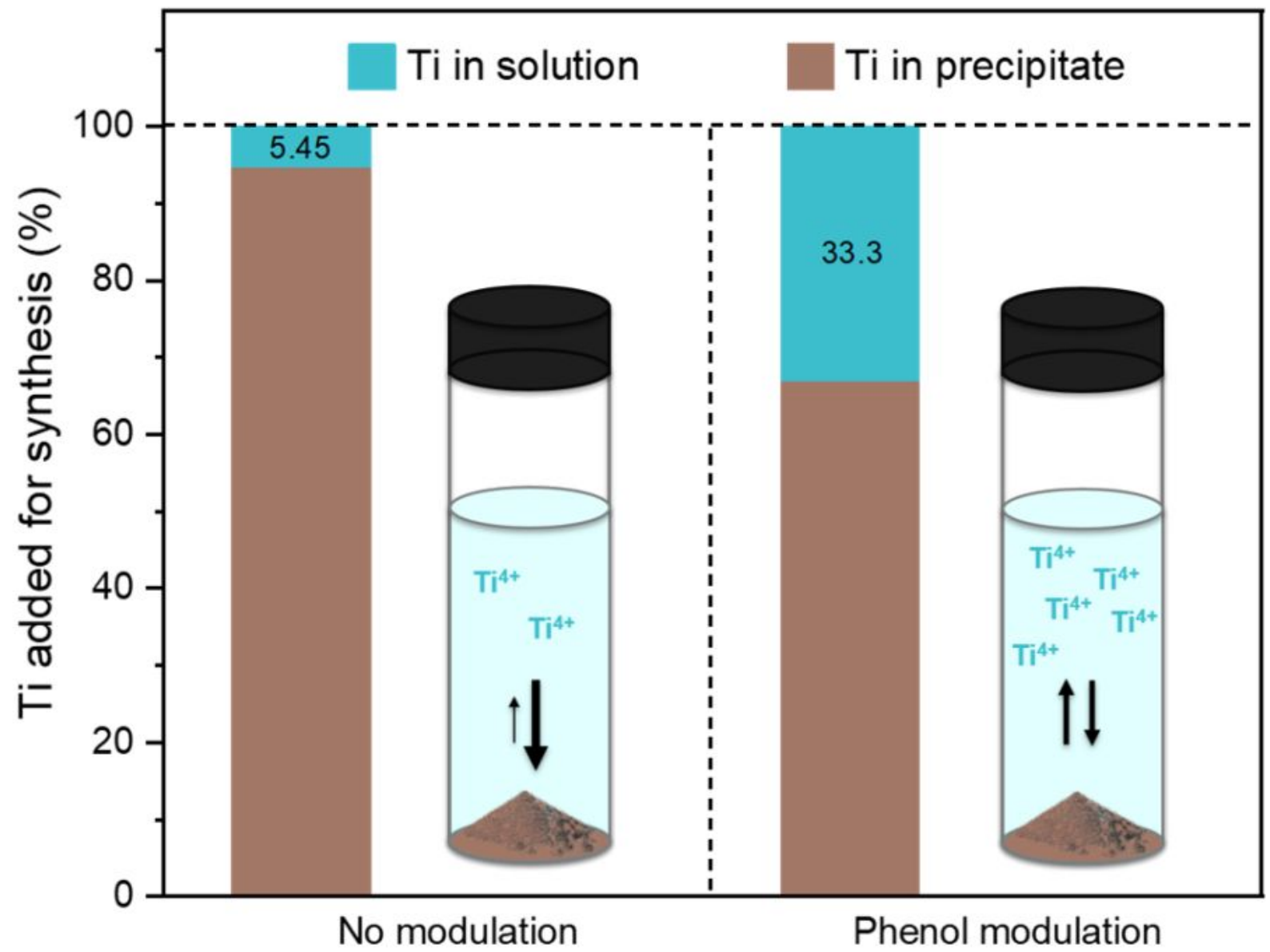

Figure S19. Ti amount found (through TXRF) in the liquid phase 5 min after adding TTIP to the EA slurry in DMF for the unmodulated (0 equiv. PhOH, left) *vs*. the modulated (240 equiv. PhOH, right) experiment. The PhOH modulation alters the binding equilibrium of Ti-EA and allows for more Ti to stay in solution, even at room temperature.

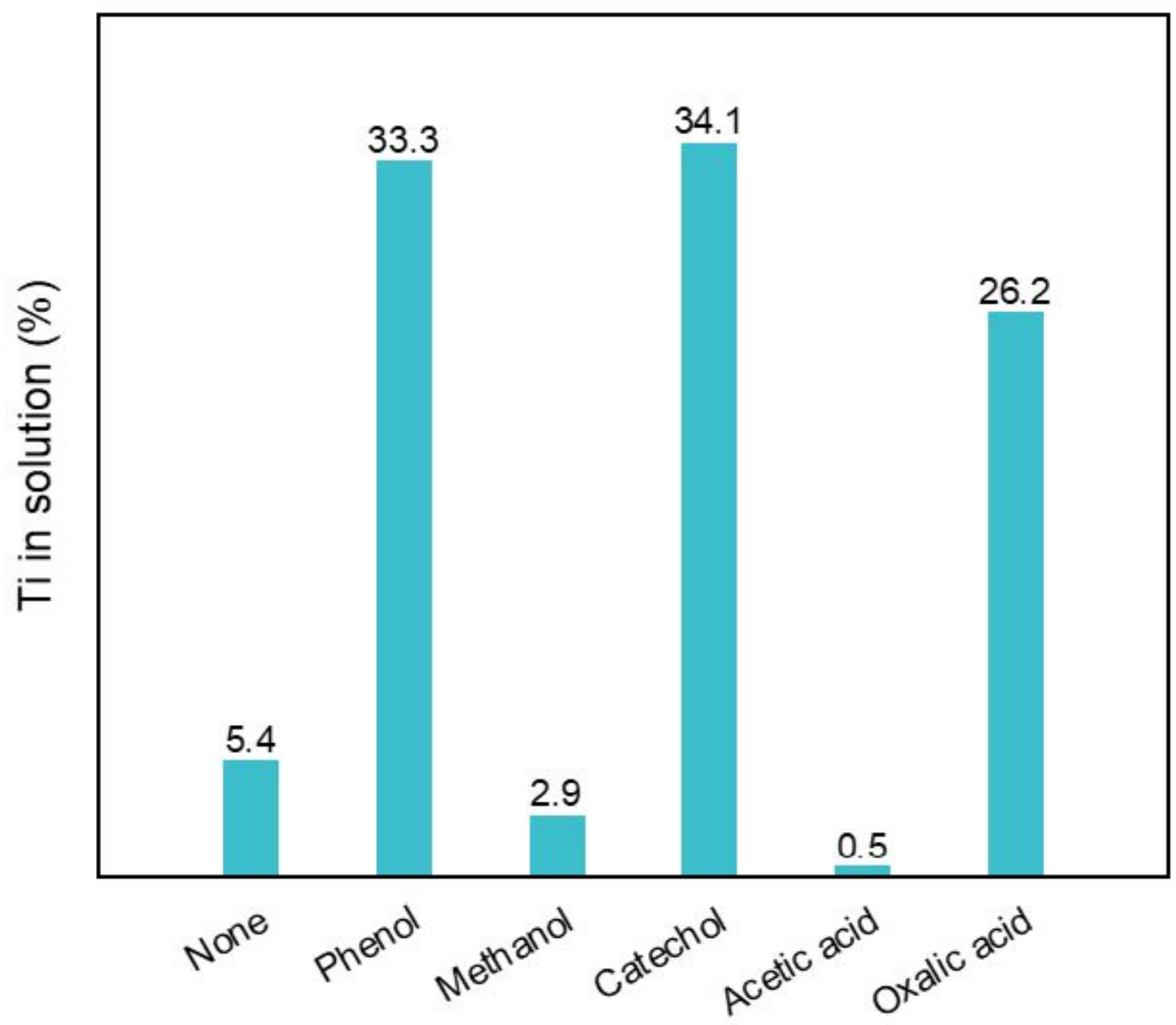

Figure S20. Ti amounts found (through TXRF) in the liquid phase 5 min after adding TTIP to the EA slurry in DMF with different modulator types (each 240 equivalents). Phenol shows a similar retention

of Ti in the solution compared to chelating modulators, such as catechol and oxalic acid. However, phenol can be exchanged during the synthesis, allowing for a controlled crystal growth.

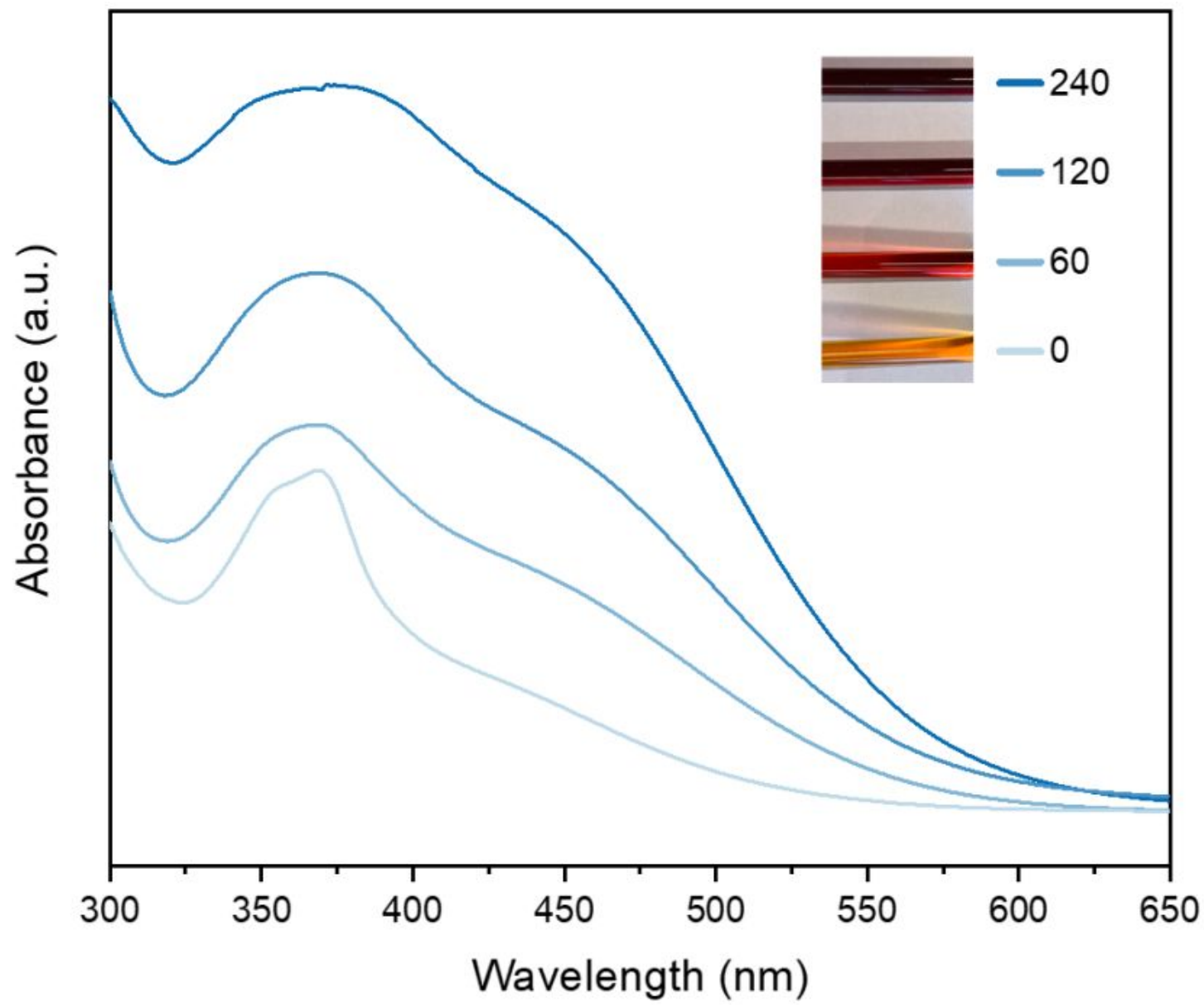

Figure S21. UV-vis absorption spectra of the supernatant of the experiments described above. With increasing amounts of phenol, the light absorption caused by Ti-phenolate complexes also increases.

4.2. Competitive coordination titration followed by NMR

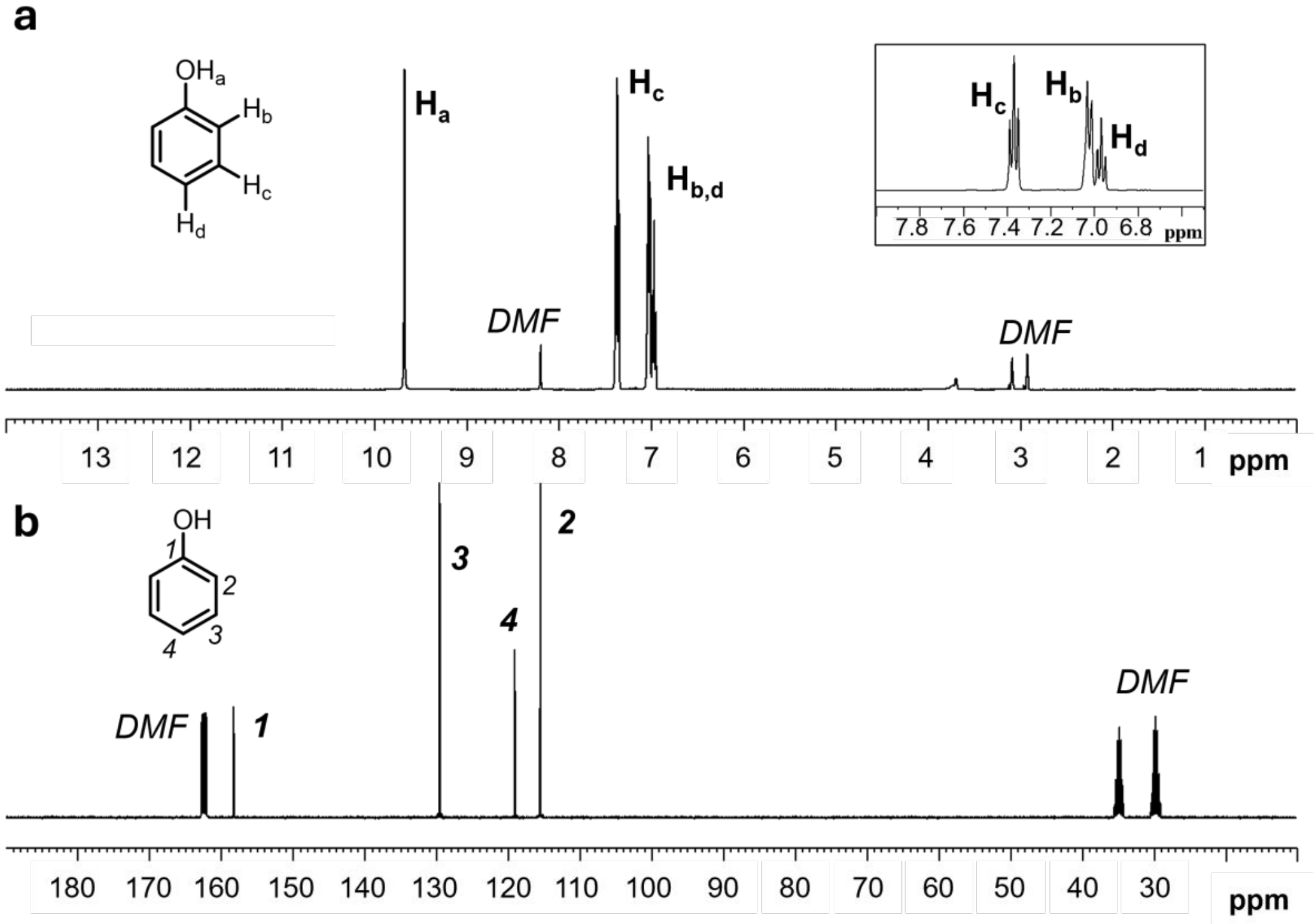

Figure S22. a) ^{1}H NMR spectrum and b) ^{13}C NMR spectrum of phenol, including assigning of all signals.

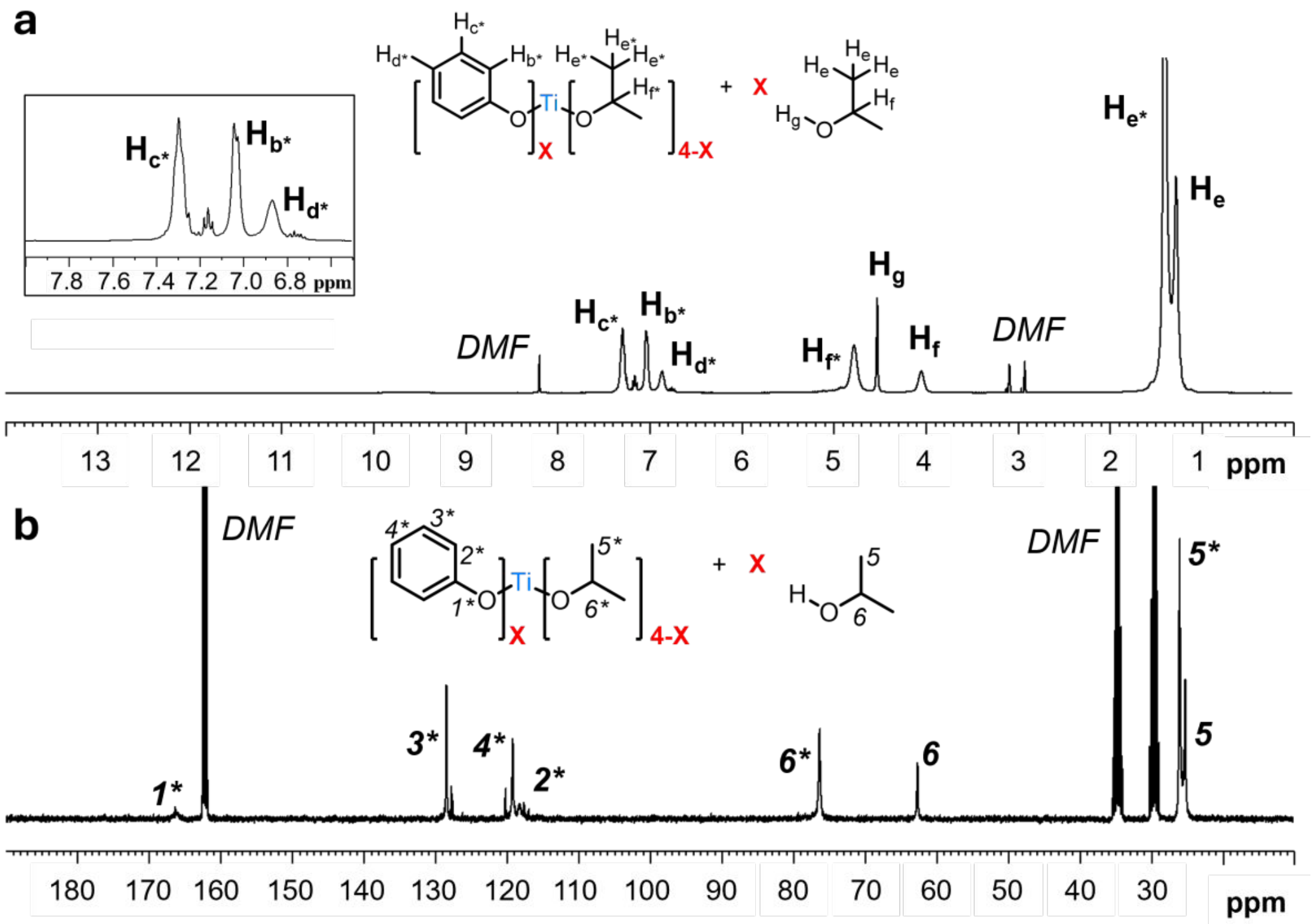

Figure S23. a) ^{1}H NMR spectrum and b) ^{13}C NMR spectrum of phenol after the addition of TTIP, including the assignment of all signals.

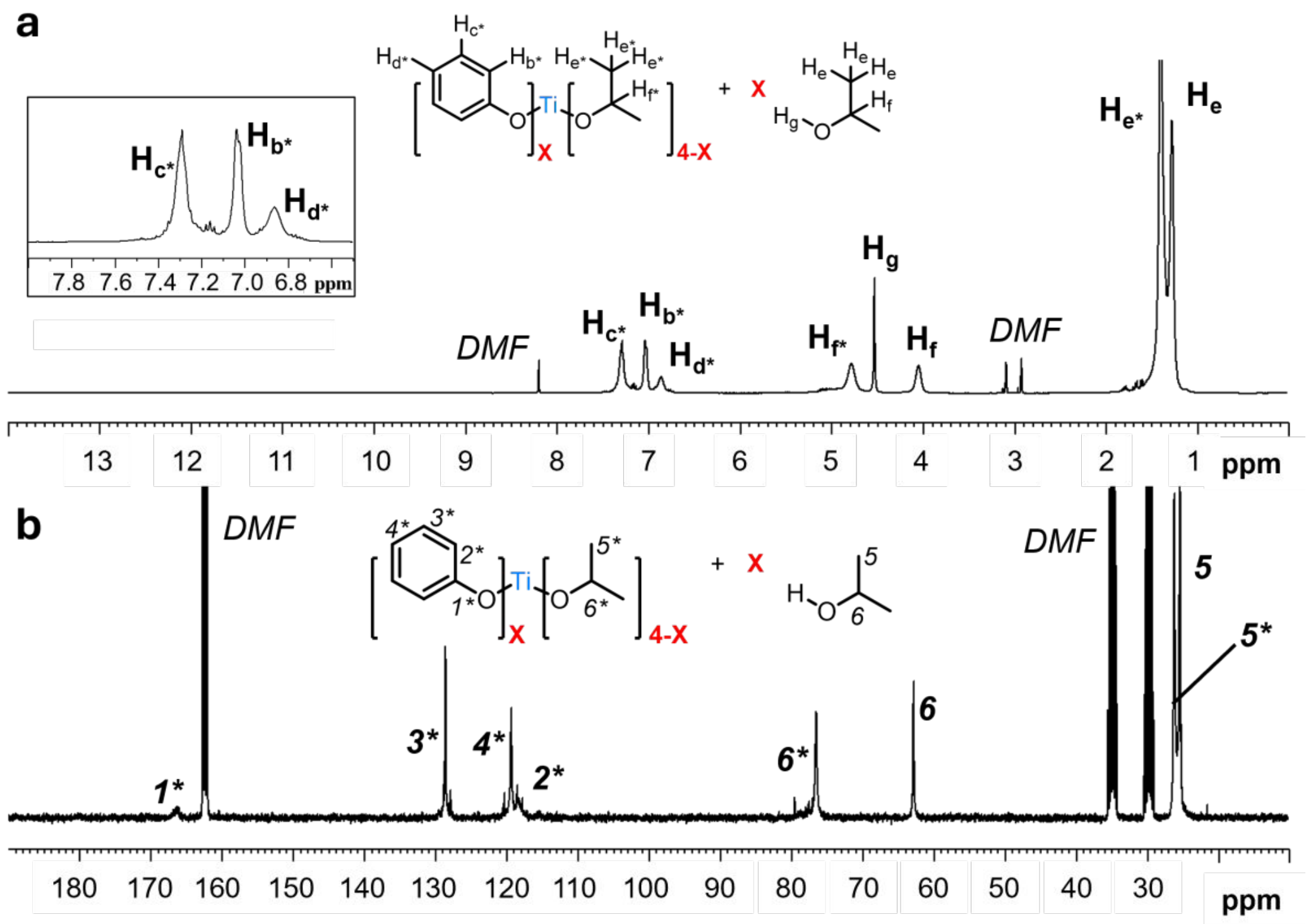

Figure S24. a) ^{1}H NMR spectrum and b) ^{13}C NMR spectrum of phenol and TTIP after the addition of 1 mg ellagic acid, including the assignment of all signals. Note here that the free isopropanol signals are increasing in intensity. The signals of ellagic acid are not visible due to possible formation/precipitation of a titanium-ellagic acid complex.

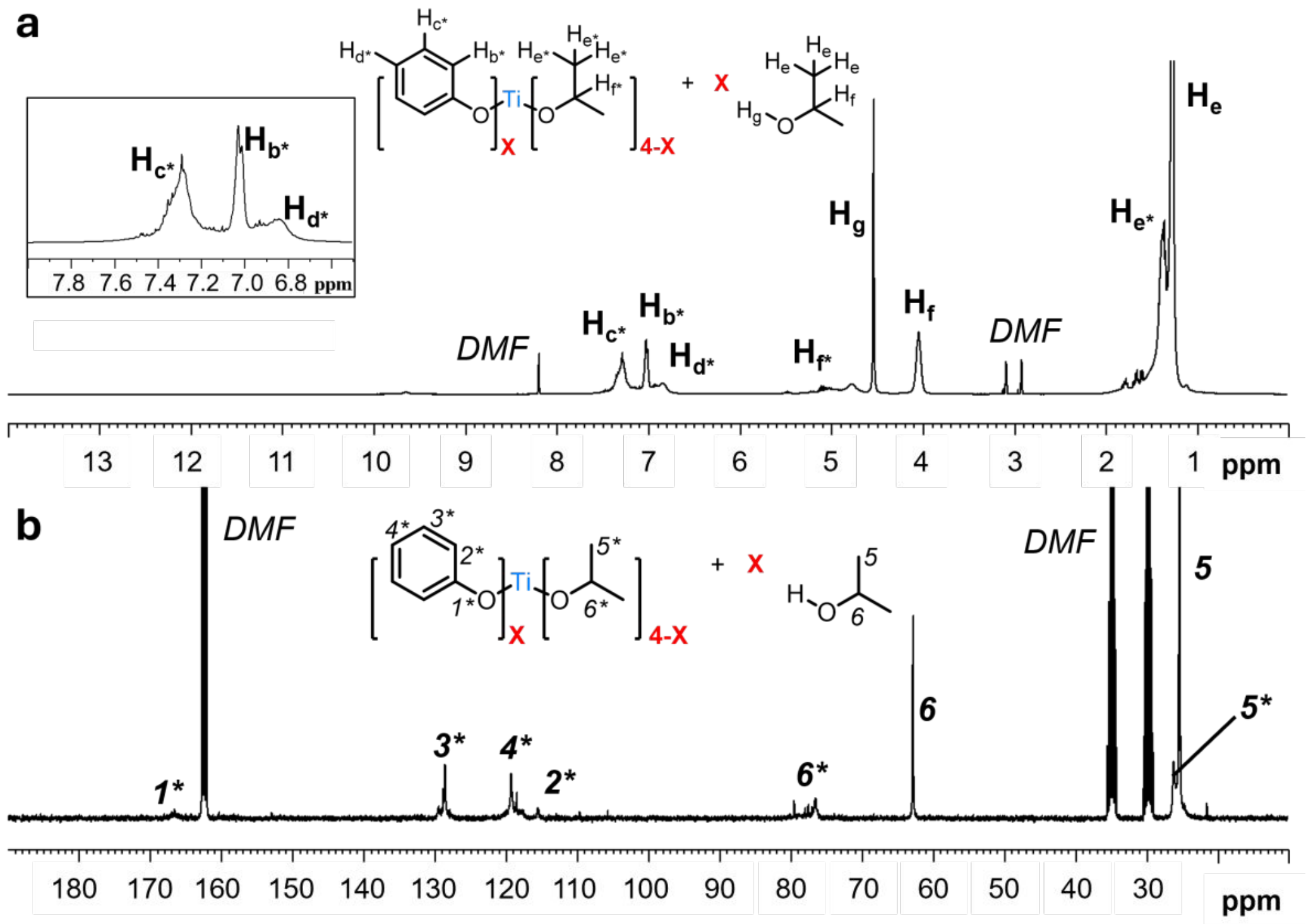

Figure S25. a) ^{1}H NMR spectrum and b) ^{13}C NMR spectrum of phenol and TTIP after the addition of 2 mg ellagic acid, including the assignment of all signals. Note here that the free isopropanol signals are

increasing in intensity. The signals of ellagic acid are not visible due to possible formation/precipitation of a titanium-ellagic acid complex.

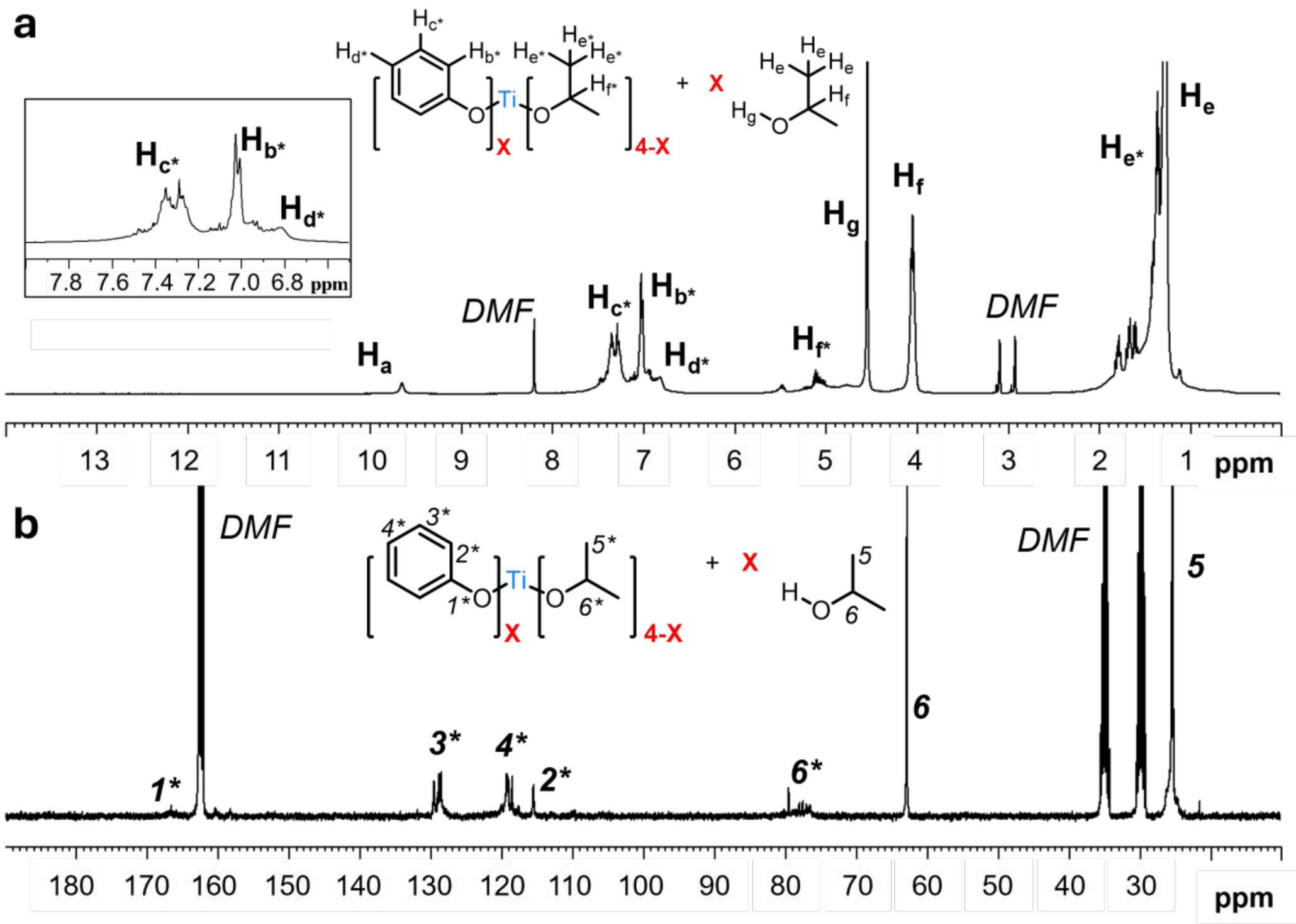

Figure S26. a) ^{1}H NMR spectrum and b) ^{13}C NMR spectrum of phenol and TTIP after the addition of 3 mg ellagic acid, including the assignment of all signals. Note here that the free isopropanol signals are increasing in intensity. The signals of ellagic acid are not visible due to possible formation/precipitation of a titanium-ellagic acid complex.

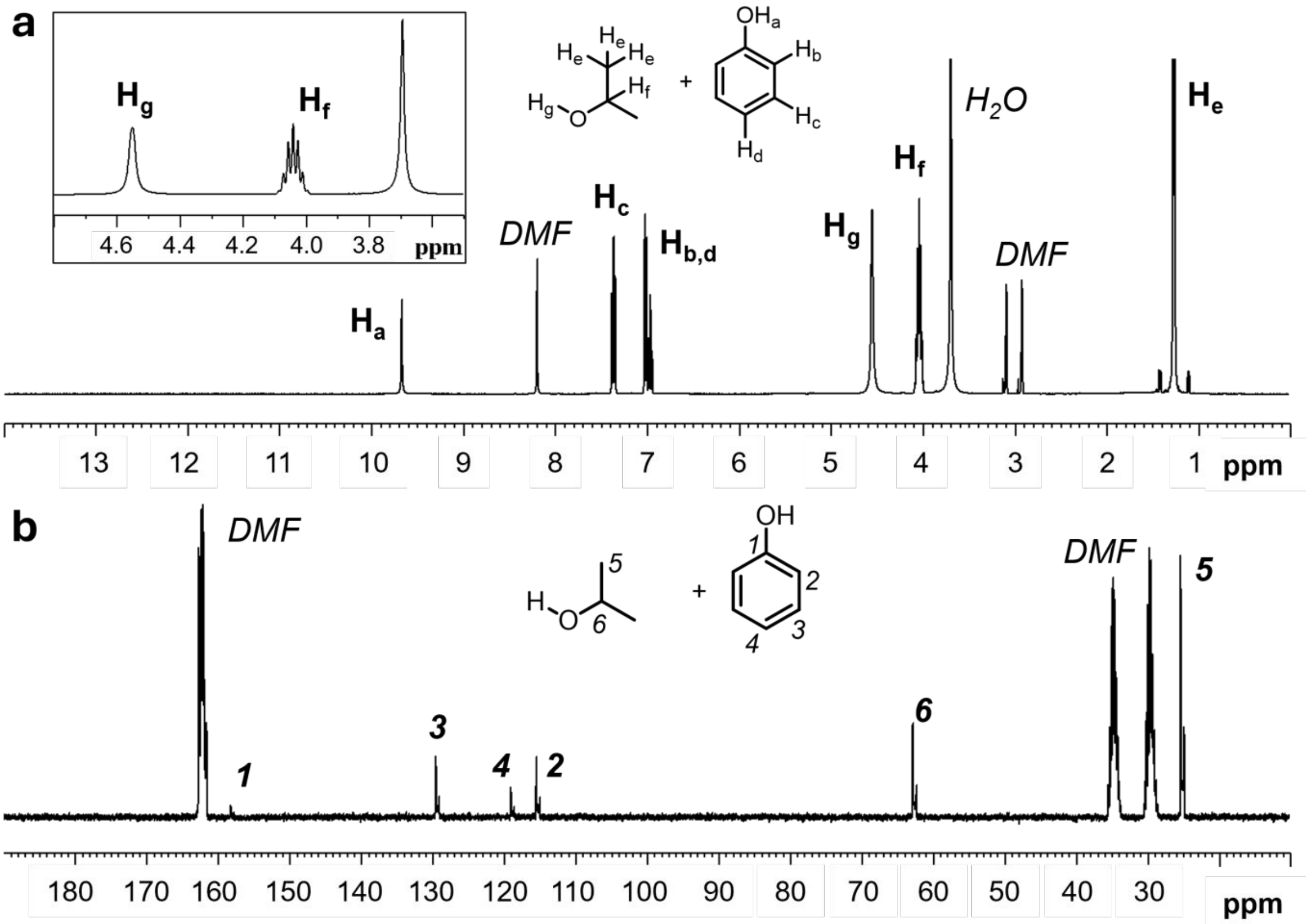

Figure S27. a) ^{1}H NMR spectrum and b) ^{13}C NMR spectrum of phenol and TTIP after the addition of 4 mg ellagic acid, including the assignment of all signals. Note here that only signals of free phenol and isopropanol are observed. The signals of ellagic acid are not visible due to precipitation of the titanium complex.

4.3. Phenol-assisted synthesis of other Ti-MOF materials

We adapted reported synthesis methods of Ti-MOFs with similar coordination motifs by exchanging the original modulators/additives by phenol. For this, we performed screenings in terms of precursor amounts, phenol equivalents, temperature, solvent composition and reaction time. The phenol-assisted procedures yielding satisfactory results and showing the transferability of this untapped modulator are described below.

Phenol-assisted **Ti-CAT-5** was synthesized by a modified method according to a previous method reported elsewhere.[9] For this, 50 mg of hexahydroxytriphenylene (HHTP) and 5 mL of anhydrous DMF with 3.4 g of phenol (240 equivalents) were charged into a 23 mL Teflon-lined autoclave. Under inert atmosphere, 45 µL of TTIP were dropped onto the slurry and the reaction mixture was stirred for 1 h. The autoclave was closed and placed into a pre-heated oven at 180 °C for 48 h. After cooling down to room temperature, the product was washed with DMF (3 x 5 mL), isolated through centrifugation and dried at 80 °C.

Phenol-assisted **IEF-11** was synthesized by a modified method according to previous methods reported elsewhere.[10,13] For this, 50 mg of squaric acid (SqA), 114 mg of $Ti(acac)_2$ and 4.3 mL of H_2O with 1.6 g of phenol (40 equivalents) were charged into a 10 mL Teflon-lined autoclave. The autoclave was closed and placed into a pre-heated oven at 120 °C for 24 h. After cooling down to room temperature, the product was washed with H_2O (3 x 4 mL), isolated through centrifugation and dried at room temperature.

Phenol-assisted **NTU-9** was synthesized by a modified method according to previous methods reported elsewhere.[11,17] For this, 950 mg of 2,5-dihydroxyterephthalic acid (H_4DOBDC) and 6 mL of DEF with 565 mg of phenol (5 equivalents) were charged into a 23 mL Teflon-lined autoclave. Under inert atmosphere, 350 µL of TTIP were dropped onto the slurry. The autoclave was closed and placed into an oven and heated to 200 °C for 20 h. After cooling down to room temperature, the product was filtered and washed with DEF (3 x 6 mL) and dried at 80 °C.

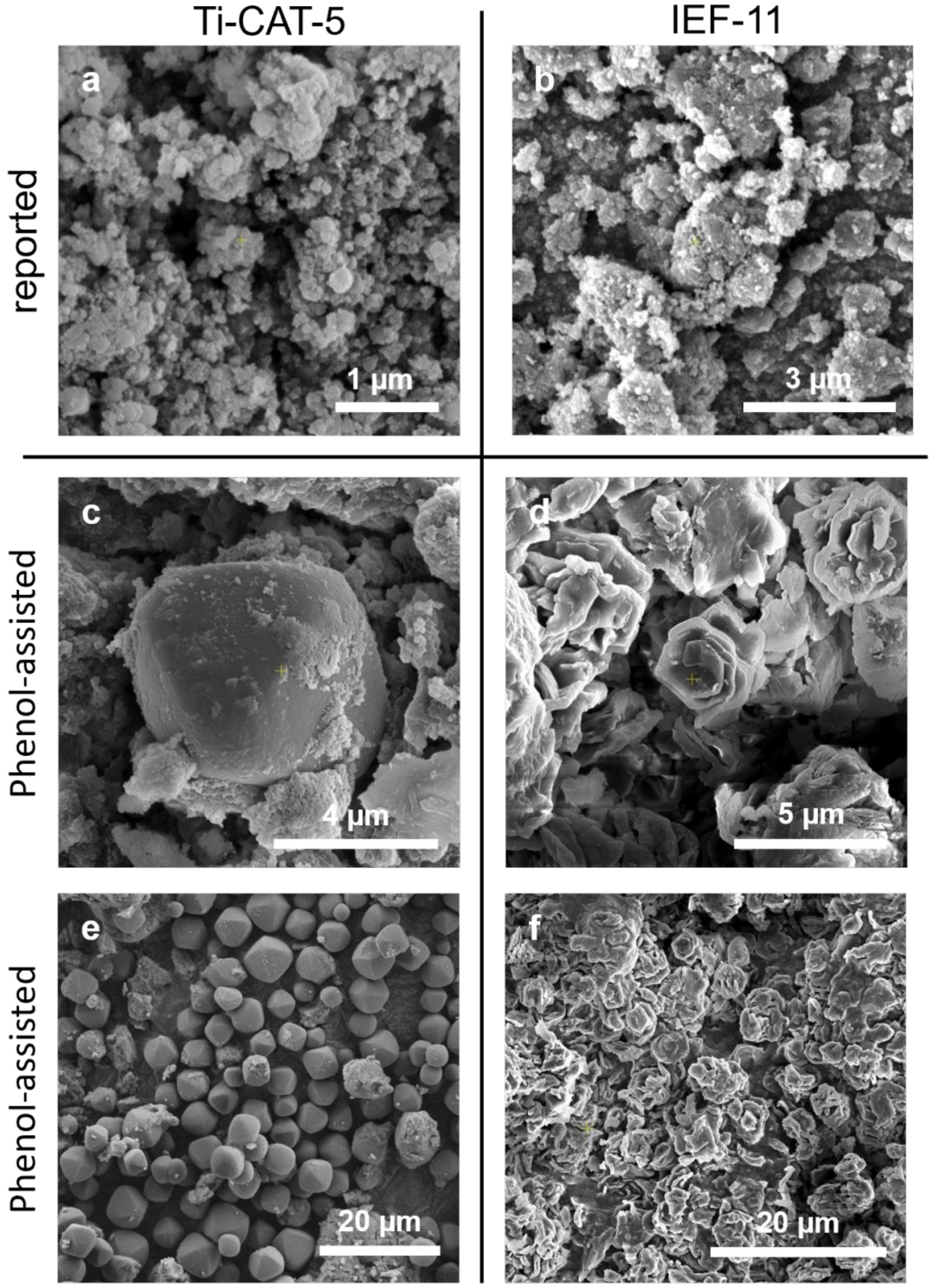

Figure S28. SEM images of Ti-MOF materials synthesized through reported *vs*. phenol-assisted methods. Left column: Ti-CAT-5 reported (**a**) and phenol-assisted (**c, e**). Right column: IEF-11 reported (**b**) and phenol-assisted (**d, f**).

Table S3. Full width at half maximum (FWHM) for characteristic peaks of phenolate and carboxy-phenolate Ti-MOF structures synthesized following reported *vs.* phenol-assisted synthesis conditions described above.

MOF	Peak position 2θ (°)	Reported synthesis FWHM (°)	Phenol-assisted synthesis FWHM (°)
Ti-CAT-5	10.9	0.22	0.09
	11.9	0.22	0.09
	23.0	0.18	0.09
IEF-11	12.5	0.41	0.28
	29.6	0.35	0.22
	37.7	0.38	0.26
NTU-9	6.9	0.45	0.30
	10.2	0.34	0.19
	15.3	0.23	0.23
COK-47(bdc)	8.3	0.64	0.88
	16.6	0.80	1.12
	25.8	0.72	1.14
MUV-11	5.6	0.33	0.25
	9.7	0.59	0.46
	15.0	0.56	0.36

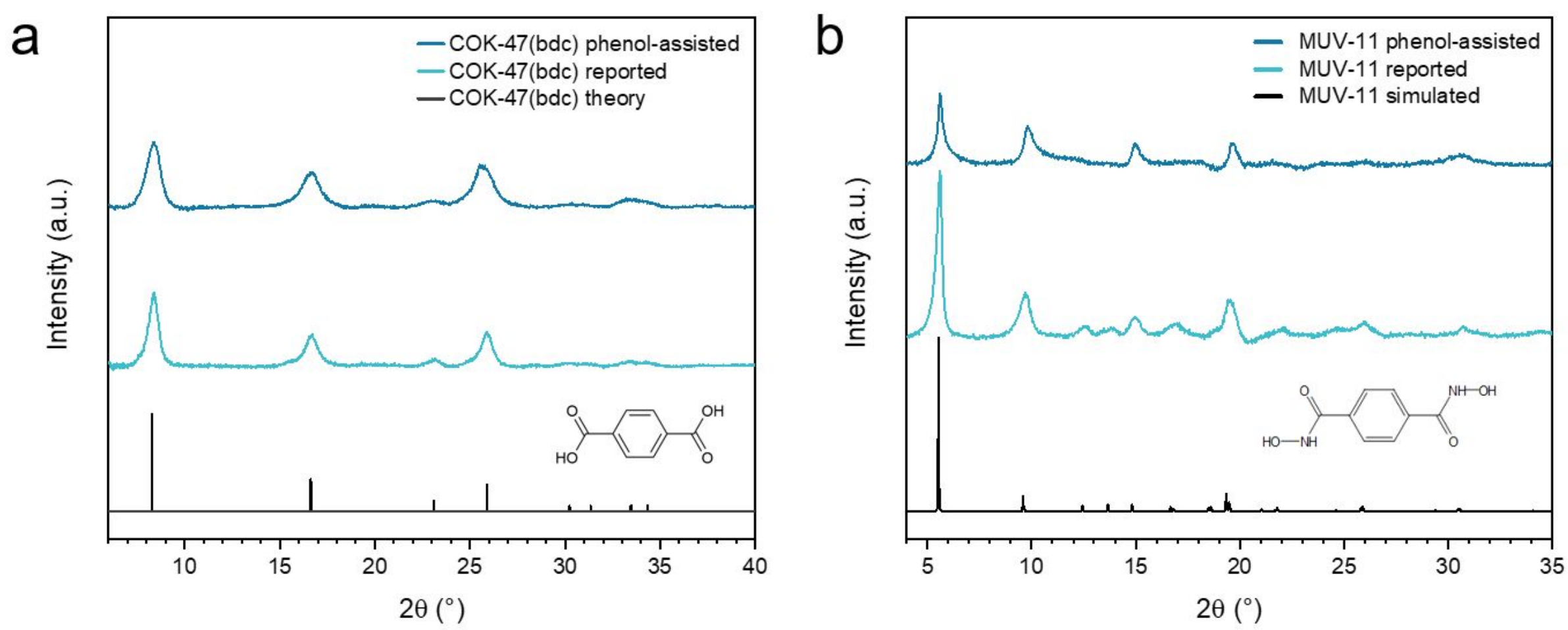

Figure S29: Capillary PXRD diffraction patterns of the obtained powders using the reported *vs.* the phenol-assisted syntheses for COK-47(bdc) (**a**) and MUV-11 (**b**).

Limitation note: Phenol modulation was also attempted for further coordination modes, i.e. carboxylate and hydroxamate Ti-MOFs including COK-47(bdc)[18], MIL-125[14], and MUV-11[12]. For this,

the reported syntheses were replicated and phenol-assisted experiments were performed by replacing formic acid, acetic acid and methanol with phenol for COK-47(bdc), MUV-11 and MIL-125, respectively. The results show that the COK-47(bdc) and MUV-11 phases are obtainable with similar crystallinity (see **Figure S29** and **Table S3**). The MIL-125 phase could not be obtained within the conditions investigated.

S5. Further characterization

5.1. Optical microscopy

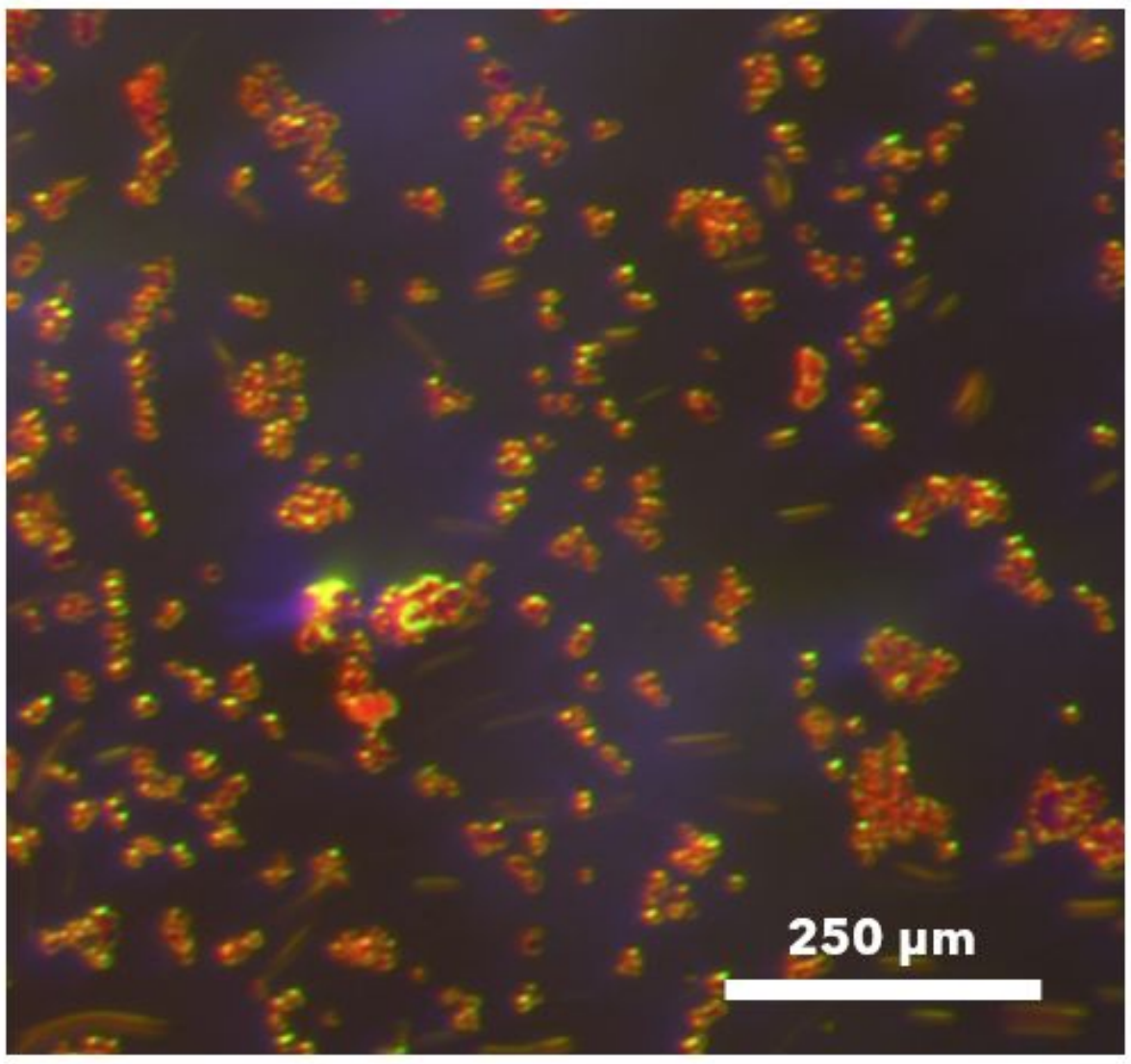

Figure S30. Optical microscopy image of the as synthesized TUW-11 red crystallites.

5.2. Continuous rotation electron diffraction (cRED)

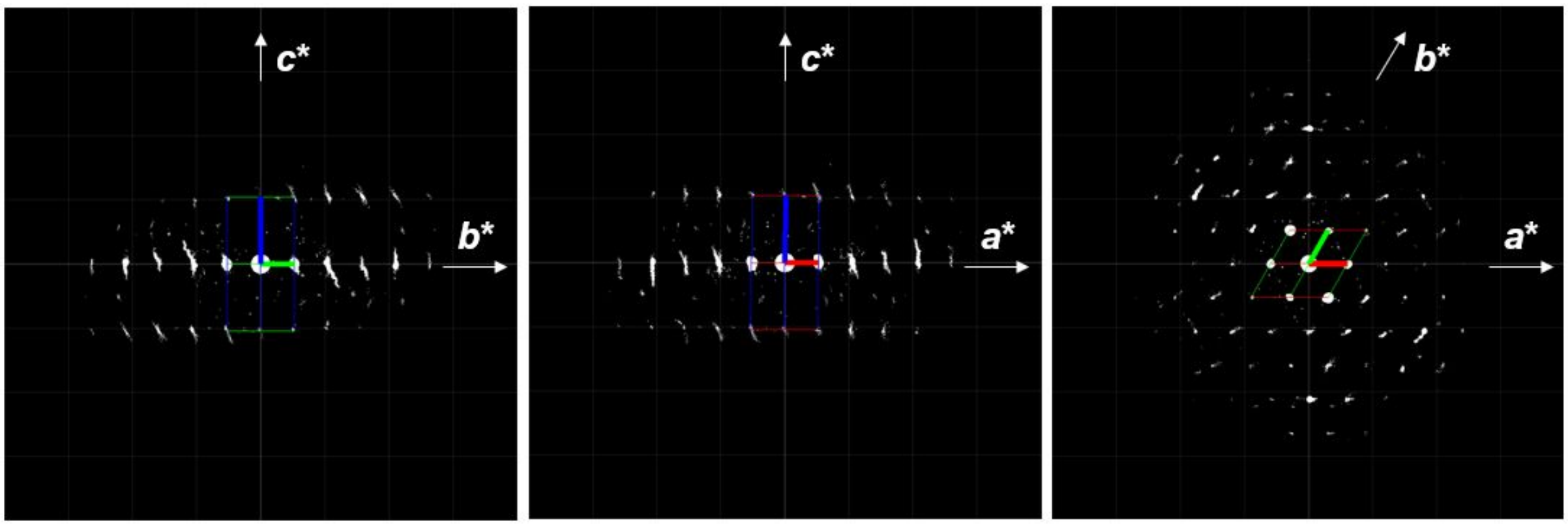

Figure S31. The 3D reciprocal lattice of TUW-11 reconstructed from the cRED data, viewing along *a**, *b** and *c**, respectively.

5.3. Scanning transmission electron microscopy (STEM)

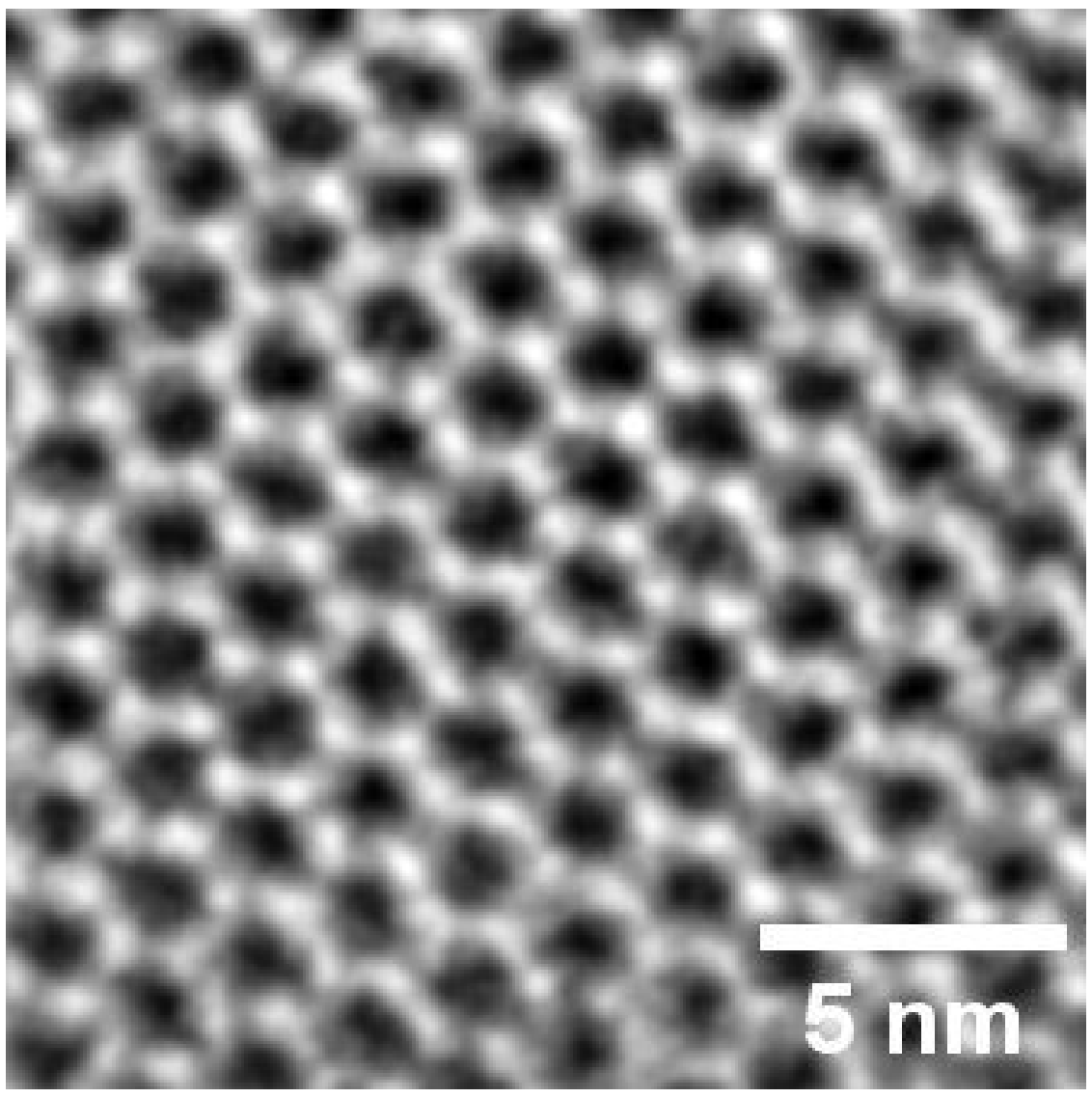

Figure S32. Annular dark-field (ADF) scanning transmission electron microscopy (STEM) image of TUW-11 viewed along [001] orientation. A bandpass filter was applied. The bright peaks in this image indicate the position of titanium in the structure.

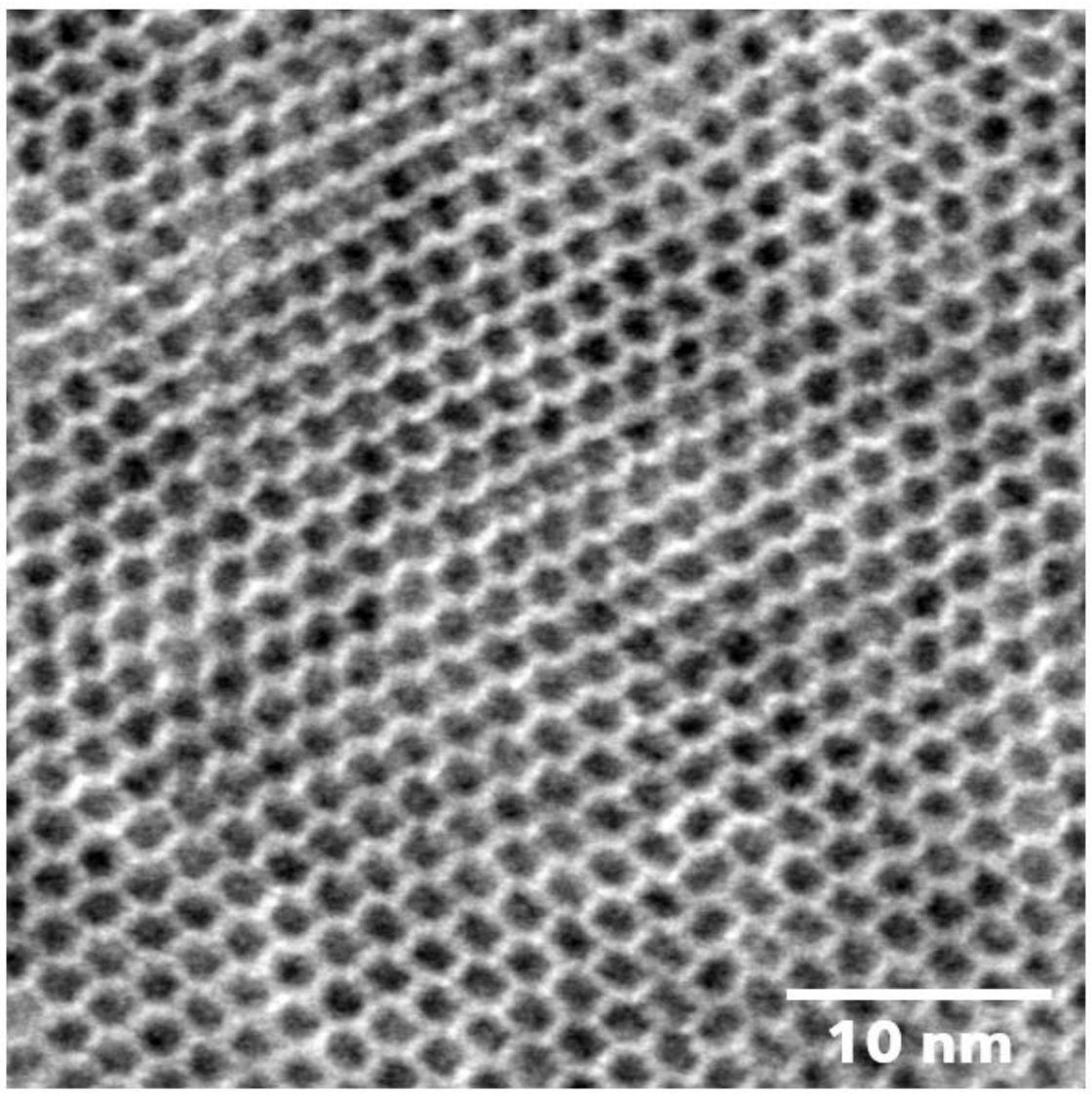

Figure S33. Integrated differential phase contrast (iDPC) scanning transmission electron microscopy (STEM) image of TUW-11 viewed along [001] orientation.

5.4. Synchrotron high resolution powder X-ray diffraction (SPXRD)

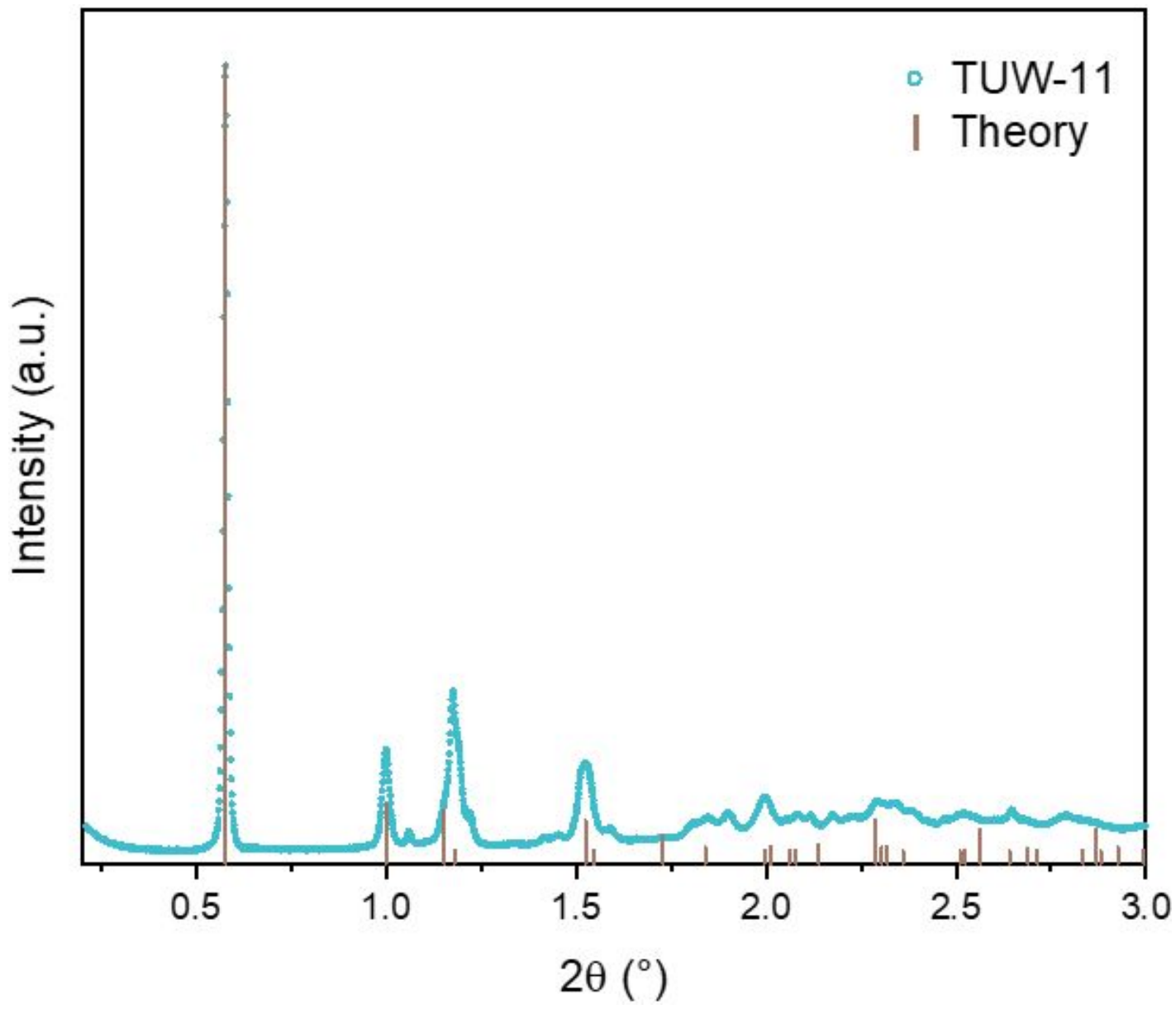

Figure S34. Experimental synchrotron powder X-ray diffraction data of as-synthesized TUW-11 MOF and the simulated pattern based on the structure model.

Table S4. Details for the Pawley fitting of TUW-11 against SPXRD data.

Sample	TUW-11
Wavelength (Å)	0.16520
Crystal system	Trigonal
Space group	$P\bar{3}1c$
Unit cell parameters (Å or °)	*a* = *b* = 18.97 Å, *c* = 9.19 Å, *α* = *β* = 90°, *γ* = 120°
R_{wp}	7.48 %
R_{exp}	1.18 %
R_p	6.6 %
GOF	6.34

5.5. X-Ray photoelectron Spectroscopy (XPS)

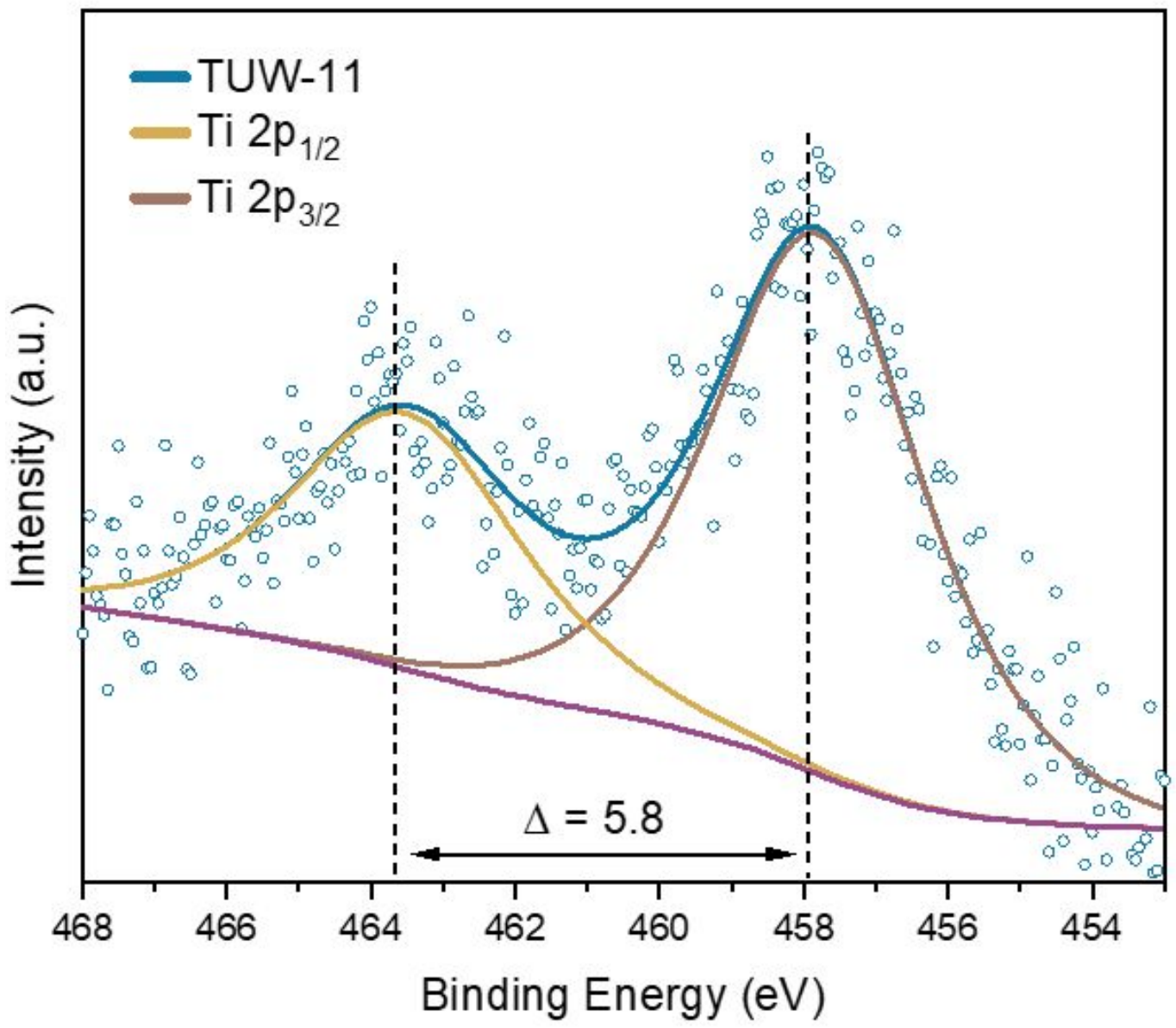

Figure S35. XPS spectrum of the as prepared TUW-11 sample in the Ti 2p edge region. Both peak positions and ratios of the Ti survey suggest an oxidation state of +IV.[19]

5.6. Extended X-ray absorption fine structure (EXAFS)

Table S5. EXAFS fitting parameters at the Ti K-edge for various samples (S_0^2 = 0.87 from Ti-foil)

	Shell	CN[a]	R[b] (Å)	σ^{2c} (Å^2)	ΔE_0^d (eV)	R factor
**TUW-11**	Ti-O_1	2.9 ± 0.1	1.98 ± 0.01	0.0030	7.8 ± 5.4	0.0197
	Ti-O_2	3.0 ± 0.1	2.01 ± 0.01	0.0030		
**Ti-foil**	Ti-Ti	2.0	2.89 ± 0.01	0.0072	5.2 ± 0.6	0.0062
**TiO_2**	Ti-O	6.0 ± 0.1	1.92 ± 0.01	0.0097	1.0 ± 0.3	0.0198
	Ti-Ti	1.0 ± 0.1	2.96 ± 0.01	0.0030		

[a] *CN*: coordination numbers; [b] *R*: bond distance; [c] σ^2: Debye-Waller factors; [d] ΔE_0: the inner potential correction. R factor: goodness of fit. Error bounds that characterize the structural parameters obtained by EXAFS spectroscopy were estimated as CN ± 20%; R ± 1 %; σ^2 ± 20%.

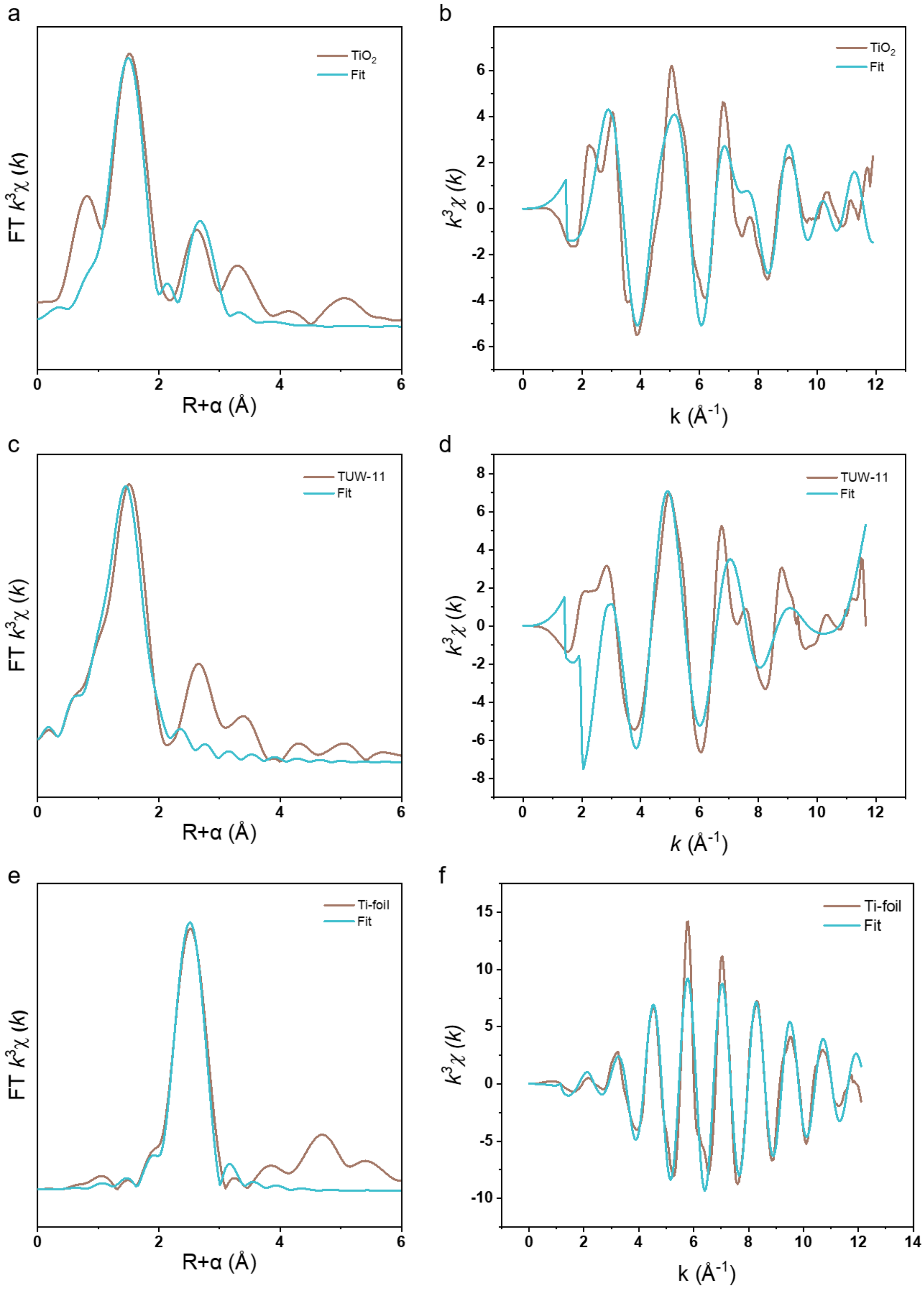

Figure S36. Fourier-transformed (FT)-EXAFS k^3-weighted $\chi(k)$ function spectra of **a)** TiO_2, **c)** TUW-11 and **e)** Ti foil. And corresponding $k^3\chi(k)$ oscillations of **b)** TiO_2, **d)** TUW-11 and **f)** Ti foil.

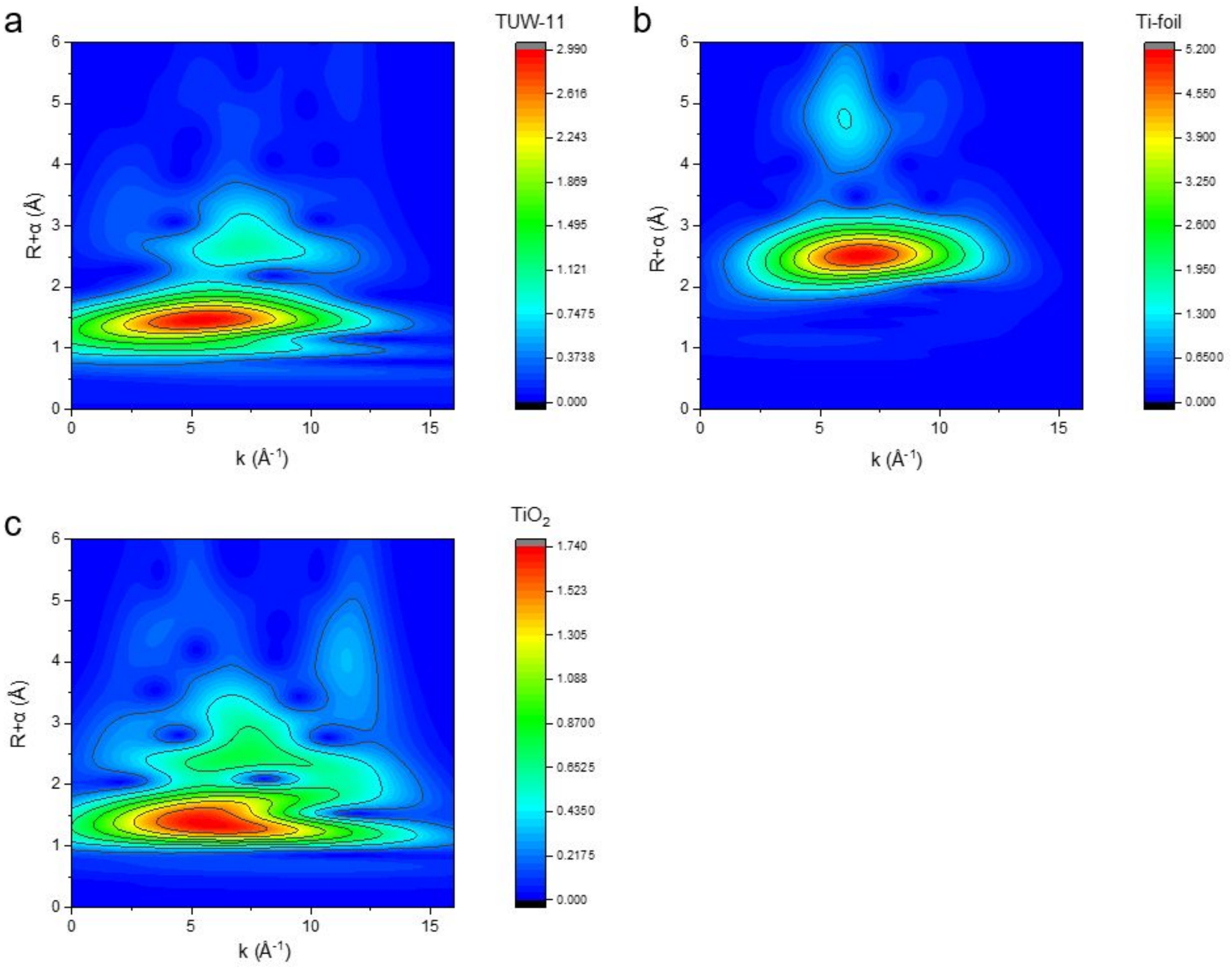

Figure S37. Wavelet transform (WT) contour plots of **a)** as synthesized TUW-11, **b)** Ti-foil and **c)** TiO_2.

5.7. Fourier-transform infrared and Raman spectroscopy (FTIR and Raman)

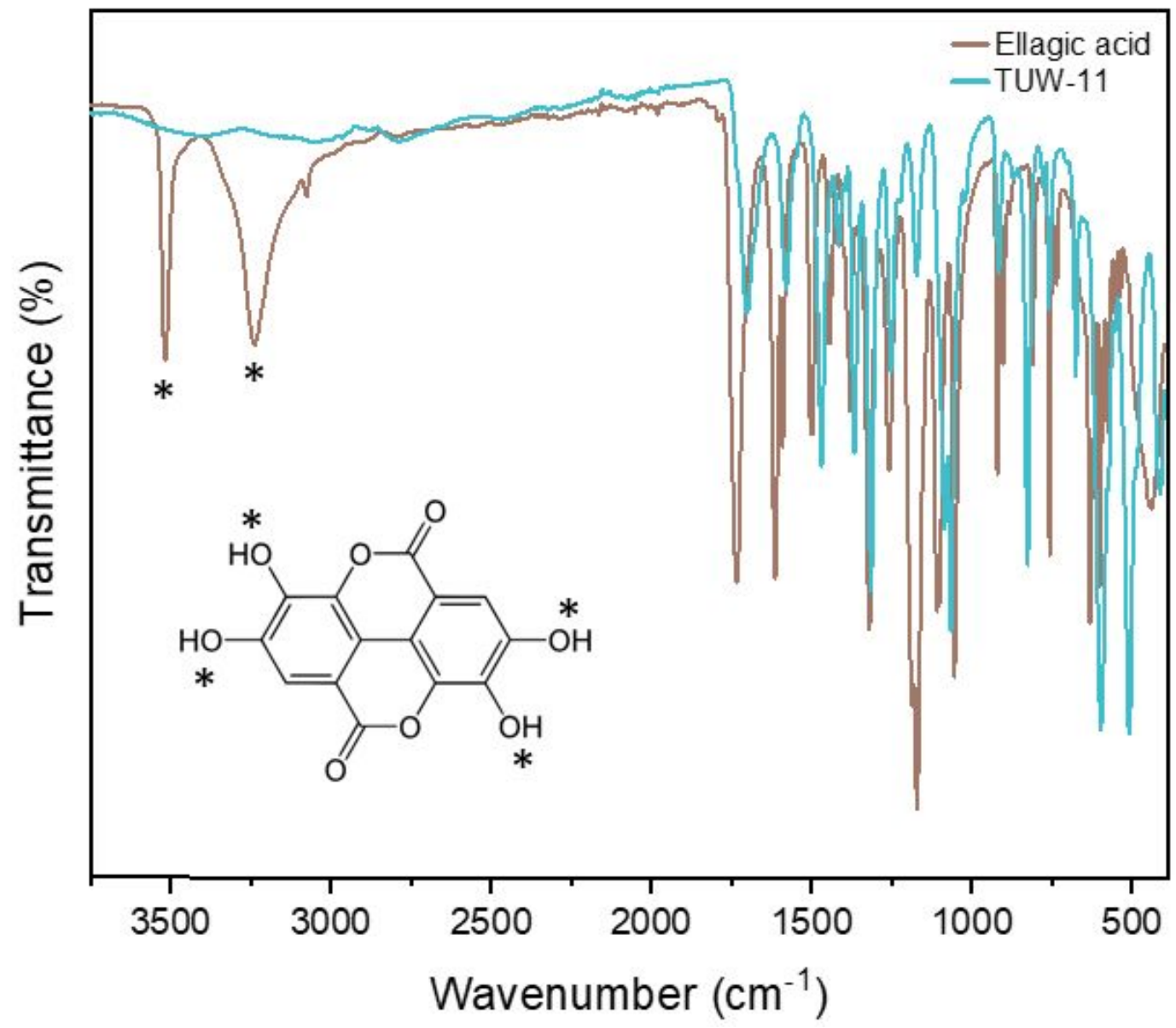

Figure S38. FTIR spectra of EA (brown) and TUW-11 (light blue). The spectra demonstrate the hydroxy bands at 3518 and 3240 cm^{-1} of the free linker disappearing in TUW-11, speaking for a lack of free hydroxy groups in TUW-11.

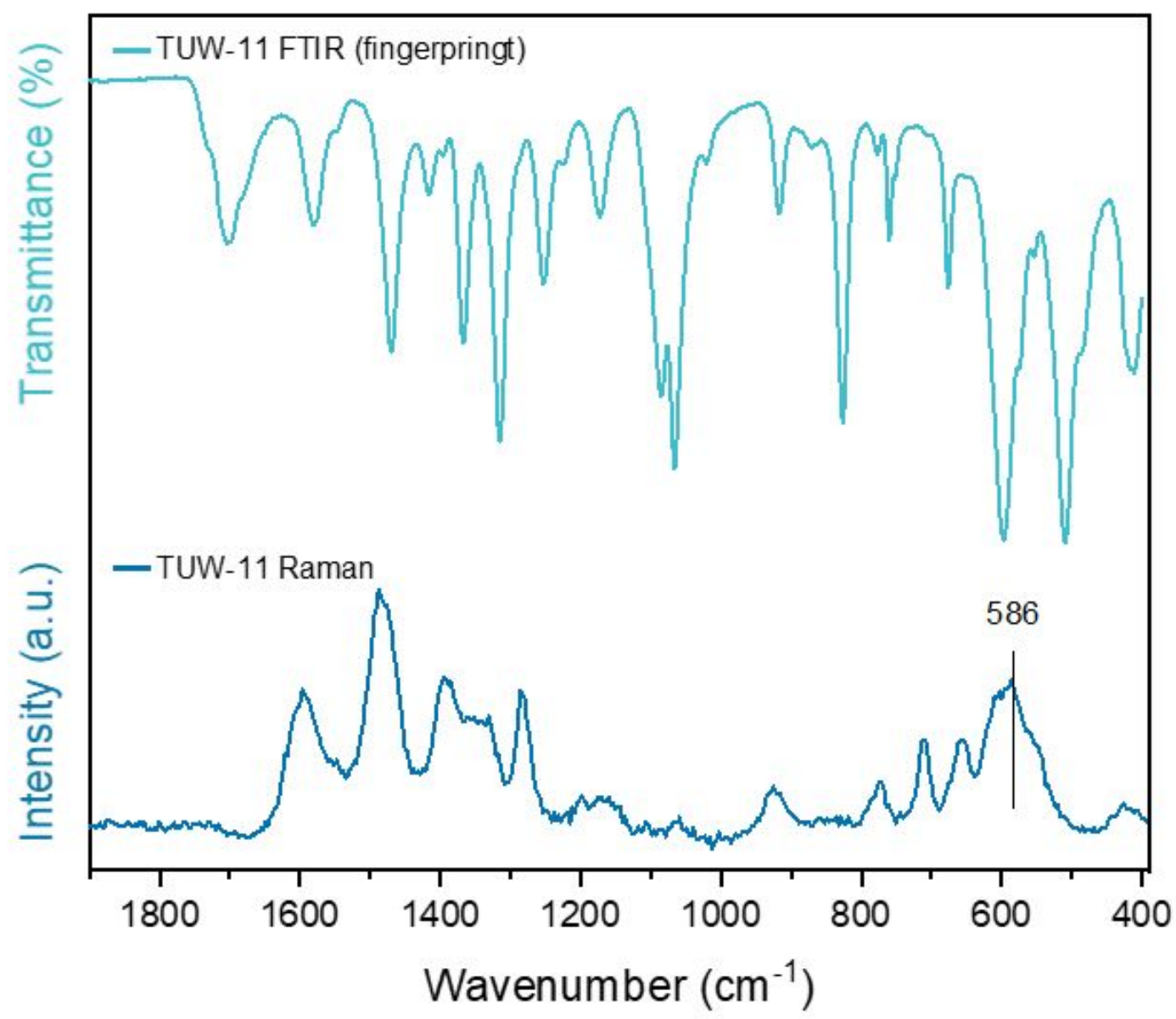

Figure S39. Superimposed FTIR (fingerprint region, light blue) and Raman (navy) spectra of TUW-11, showing the prominent metal-oxygen stretching mode from the chelate linker coordination to metal centers at 586 cm^{-1}.[20,21]

5.8. ^{1}H-NMR of digested TUW-11

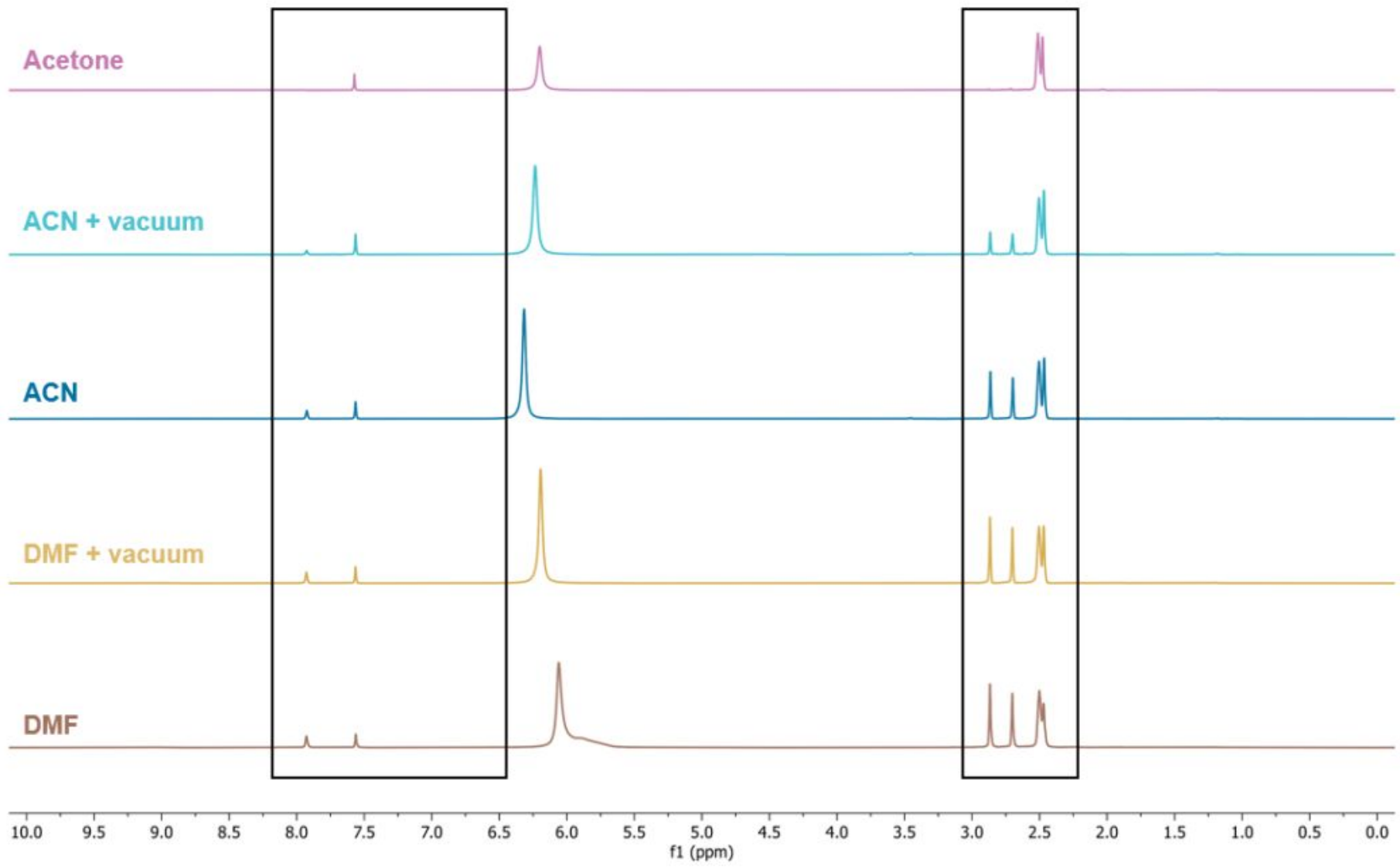

Figure S40. ^{1}H-NMR spectra of digested TUW-11 solids (5 mg) in DMSO-d_6 (500 µL) and concentrated DCl (20 µL) for different washing procedures. DMF (brown), DMF + vacuum (yellow), ACN (navy), ACN + vacuum (light blue), Acetone (pink). The peak at 6.2 ppm is related to adsorbed H_2O by DMSO-d_6, which exchanges protons with DCl.

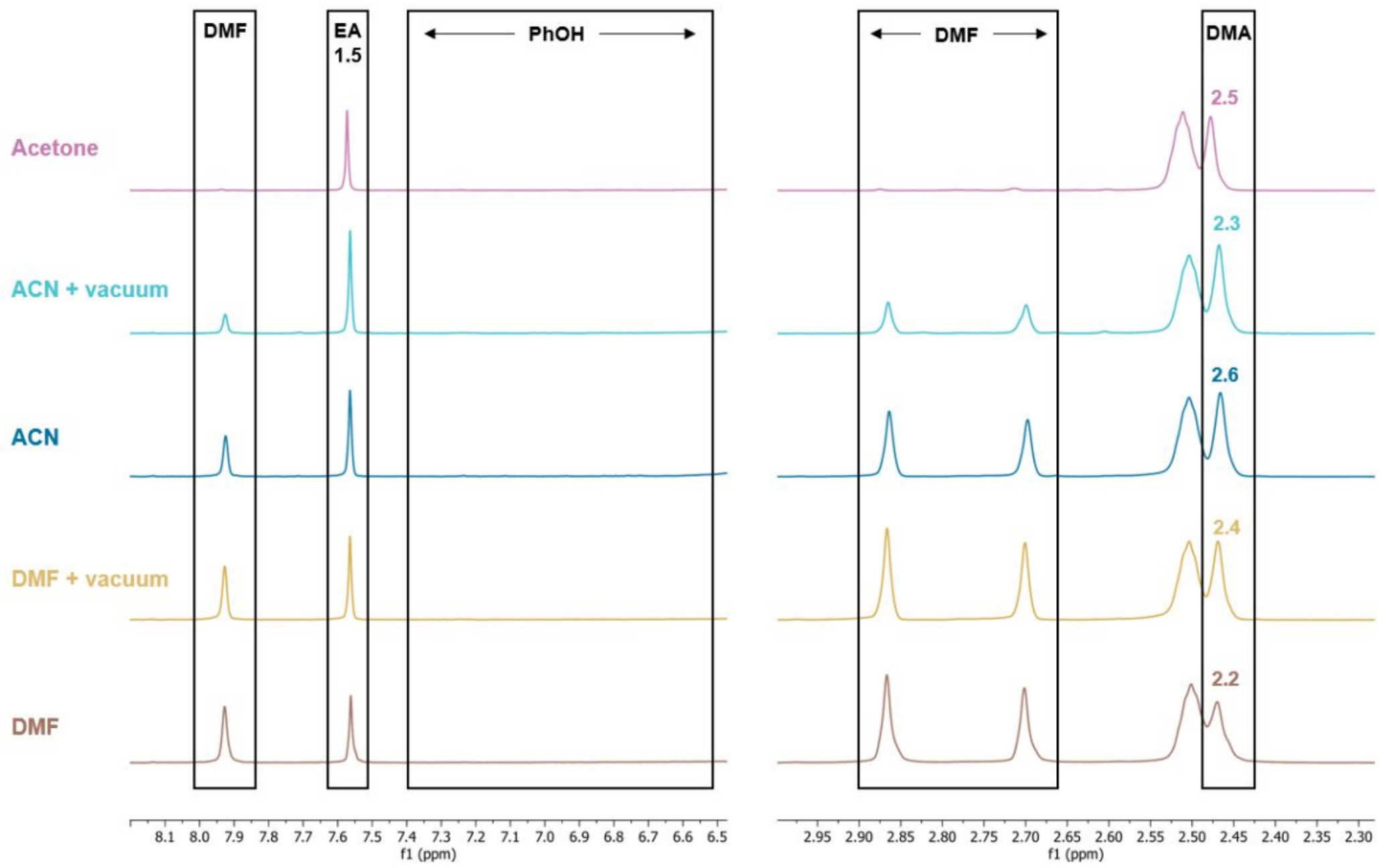

Figure S41. Detail of the above shown spectra showing specific signals for DMF, EA, and DMA, showing acetone's ability to remove DMF, while also preserving the structural integrity of TUW-11 (see **Figure S40** for PXRD). Furthermore, the relative intensities of the peaks show an average relative molar ratio of 1.5 to 2.4 for EA:DMA, going well in line with the proposed molecular formula. A lack of PhOH-specific peaks can also be observed, excluding residual phenol in TUW-11. The peak at 2.5 is related to DMSO-d_5.[22]

5.9. Solvent exchange

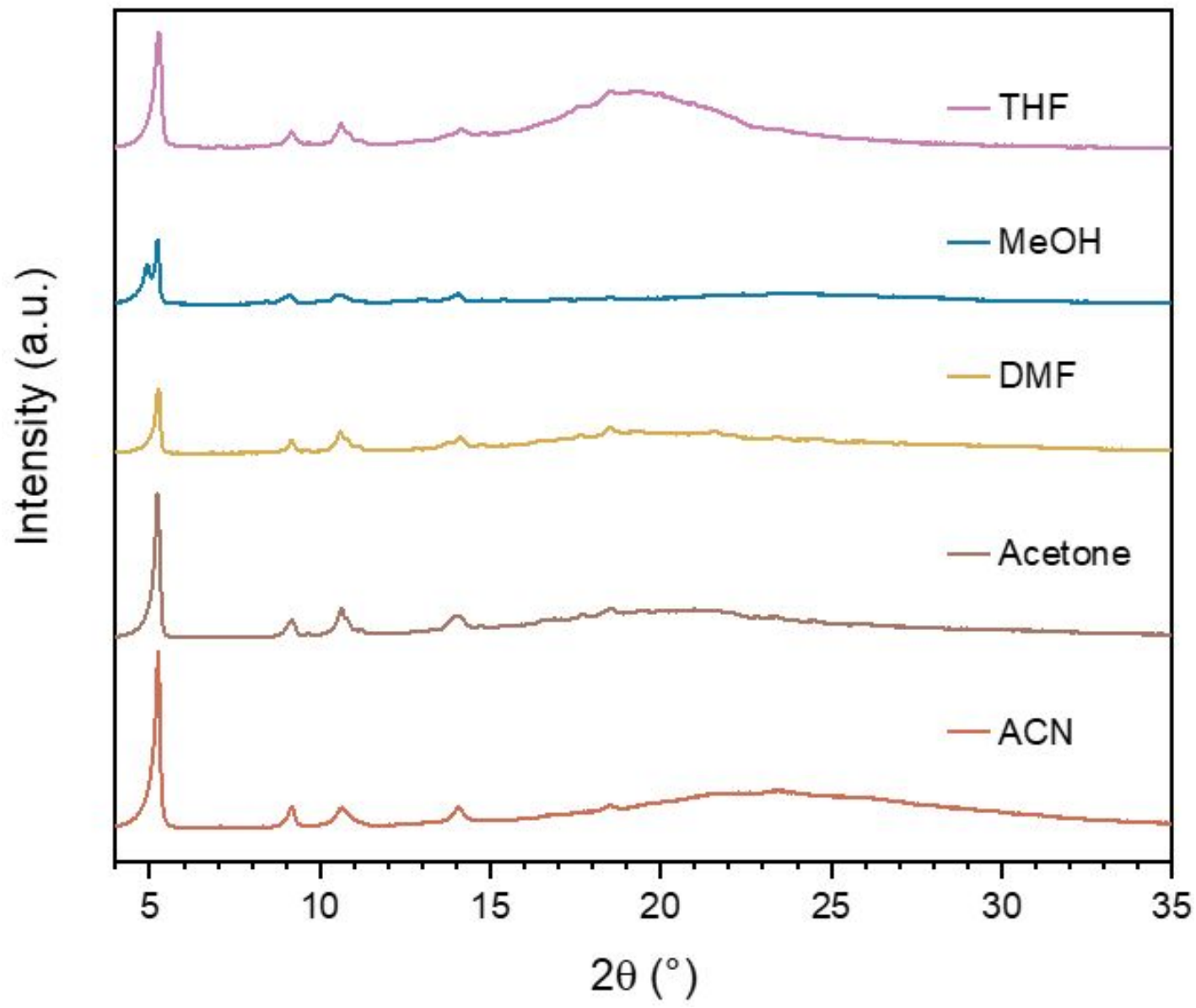

Figure S42. Capillary PXRD patterns of the solvent exchanged (3x, overnight) TUW-11 samples measured in a 1.0 mm capillary. Note: the observed peak splitting may indicate a possible solvent-induced framework distortion, analogous to the distorted phase recently reported for the similarly layered framework NTU-9.[23]

5.10. Thermogravimetric analysis (TGA)

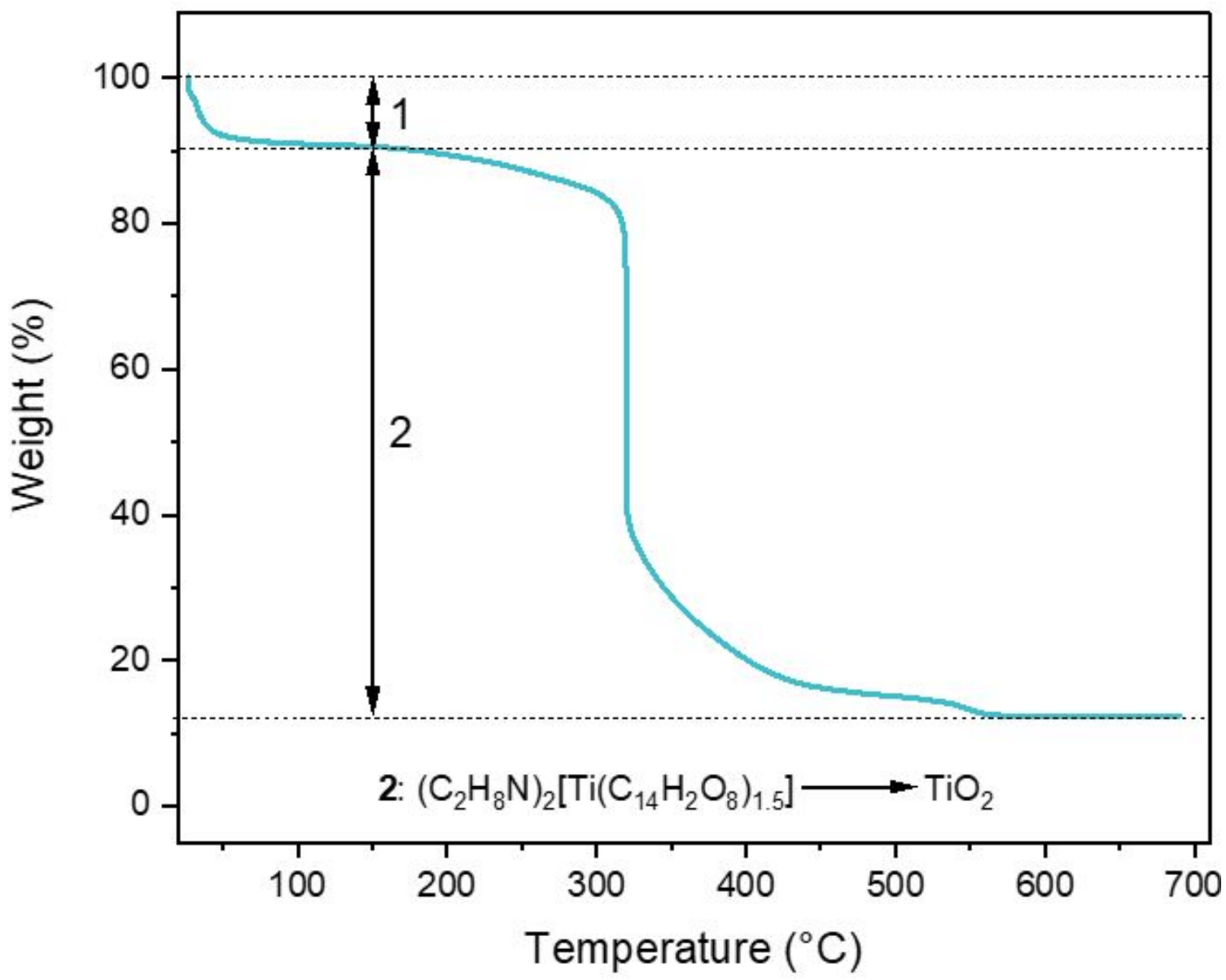

Figure S43. TGA curve under air of TUW-11 (acetone washed and activated in vacuum at 120 °C overnight), showing the expected organic mass loss from the theoretical formula $(C_2H_8N)_2[Ti(C_{14}H_2O_8)_{1.5}]$

5.11. Cation exchange

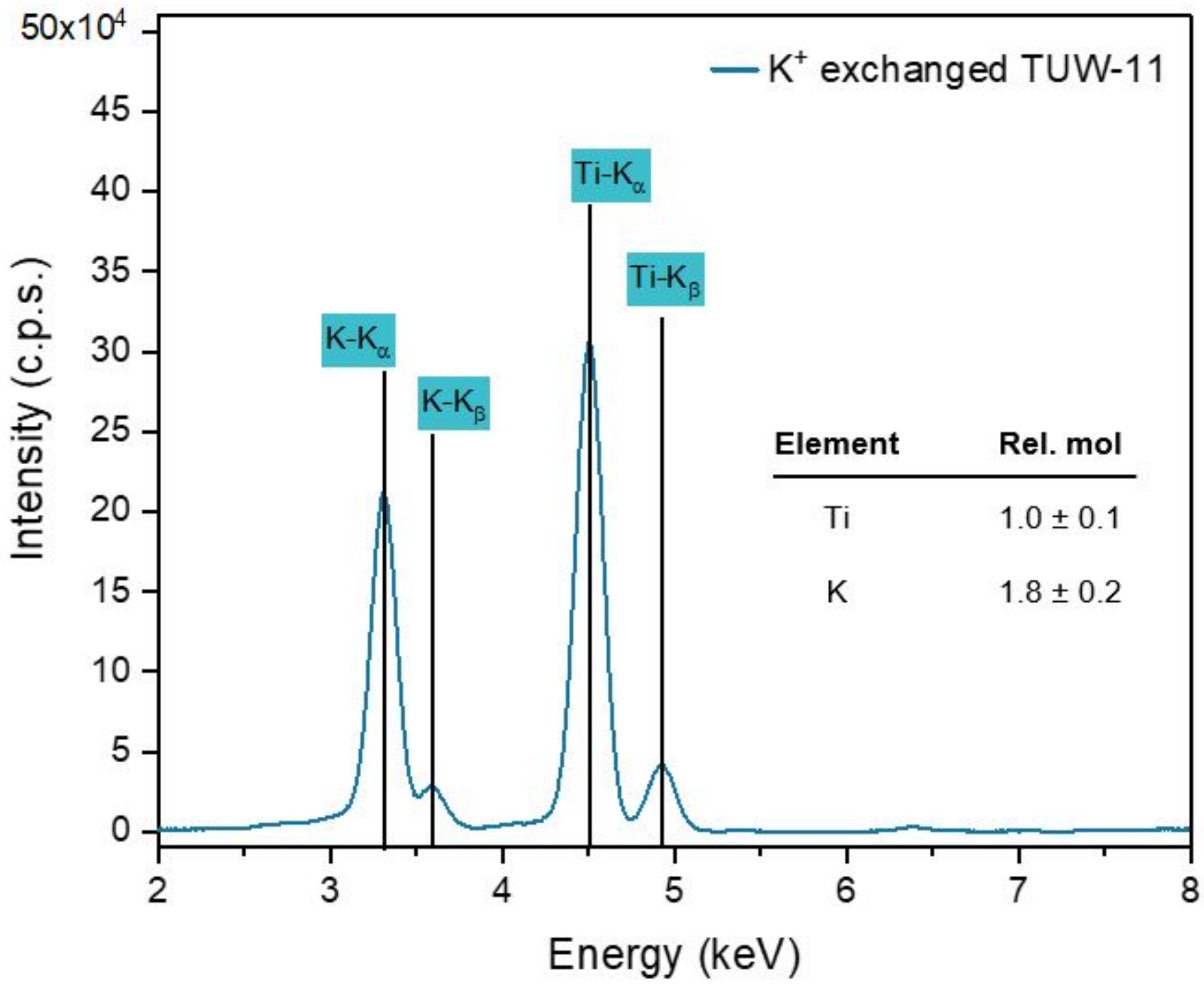

Figure S44. XRF spectrum of the cation exchanged TUW-11. The as-prepared material was solvent exchanged with a fresh saturated KCl aqueous solution, stirred vigorously and left overnight three times. Afterwards, the material was washed with fresh DI water 3x and then dried at 60 °C. The spectrum of the solid shows the characteristic lines for Ti and K. The quantification results in relative molar ratios of 1.0 : 1.8, aligning well with the proposed molecular formula of TUW-11.

5.12. pH stability

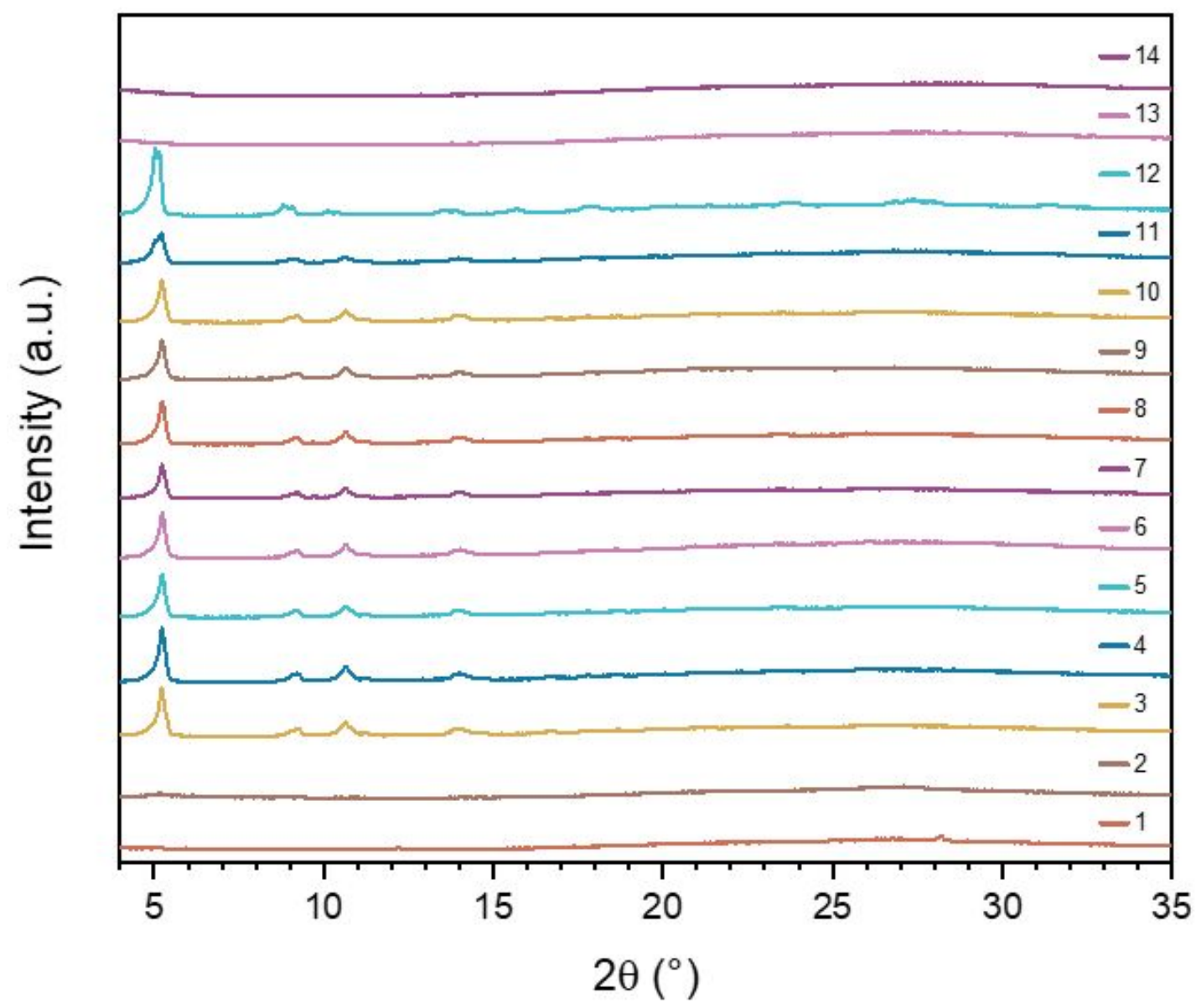

Figure S45. Capillary PXRD patterns of TUW-11 powders immersed in different pH aqueous solutions. pH < 7 was prepared with HNO_3 and pH > 7 with NaOH. The powders were dispersed in the respective solutions (c = 5 mg mL^{-1}), then left for 24 h and subsequently transferred to a 1.0 mm capillary for measurement. The structural stability window of pH = 3-12 is observed for TUW-11.

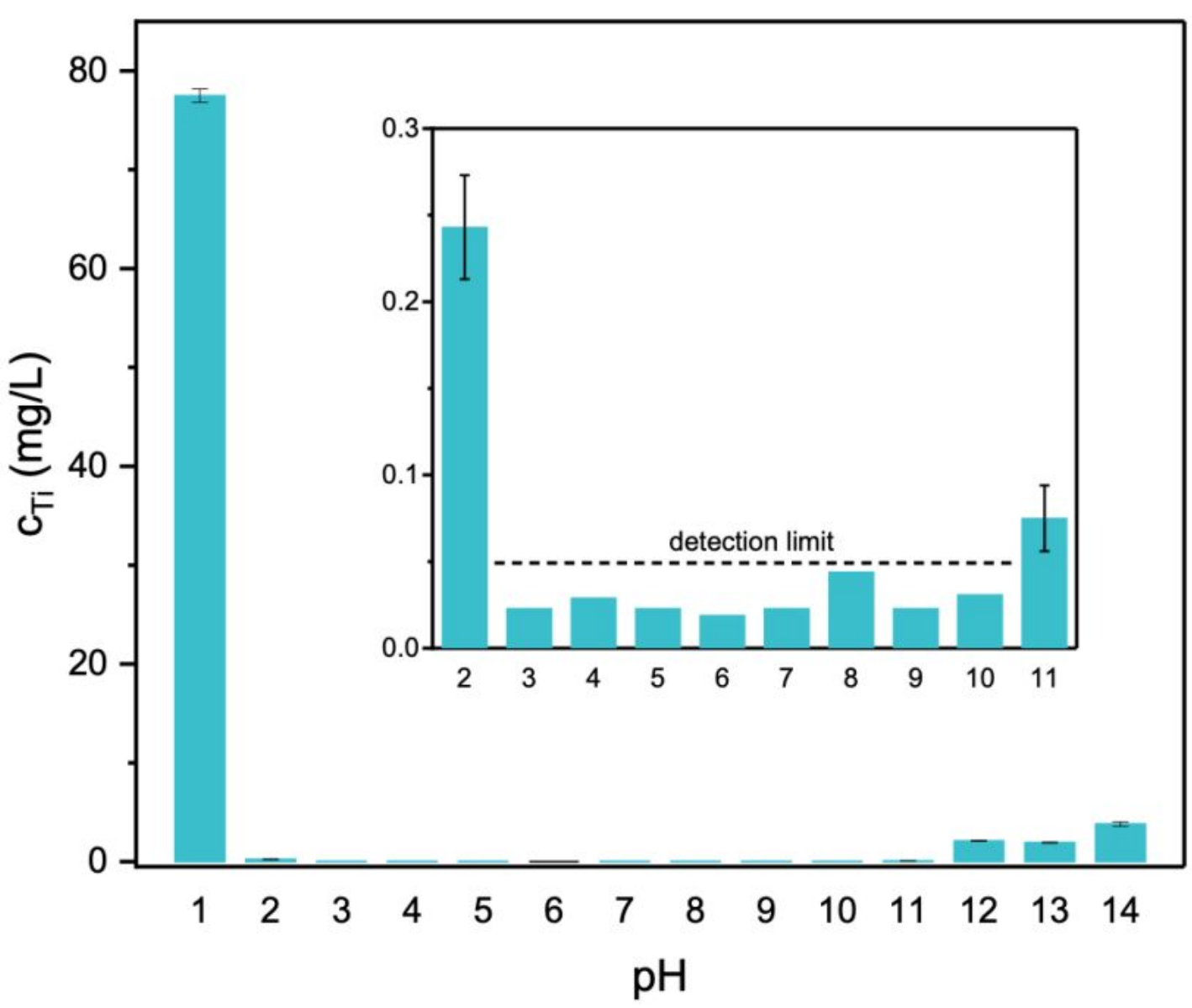

Figure S46. Concentration of Ti found through TXRF in the liquid phase after pH-treatment. Inset: detail for low concentrations.

Table S6. pH changes and Ti leached after pH-treatment

pH	pH after immersion	Ti concentration (mg L^{-1})	Ti (ng)	Ti leached (%)
1	1.1	77.48 ± 0.69	1.91×10^5	19.0
2	2.3	0.243 ± 0.03	510	0.06
3	3.9	< 0.023	< 50	< 0.01
4	4.4	< 0.029	< 64	< 0.01
5	4.6	< 0.023	< 53	< 0.01
6	4.7	< 0.019	< 42	< 0.01
7	4.9	< 0.023	< 49	< 0.01
8	5.1	< 0.044	< 99	< 0.01
9	4.6	< 0.023	< 46	< 0.01
10	5.2	< 0.031	< 63	< 0.01
11	7.9	0.075 ± 0.02	185	0.02
12	11.5	2.100 ± 0.06	4.33×10^3	0.52
13	12.7	1.936 ± 0.06	4.69×10^3	0.47
14	13.9	3.818 ± 0.22	9.24×10^3	0.94

"<" indicates maximal concentrations below the detection limit of the device (in this case 50 ppb). Maximal amounts of Ti ng are given that could still exist below the detection limit, together with the corresponding theoretical % of Ti leached this would account for.

$$\text{Ti leached \%} = m_{(solution)} / m_{(MOF)} \times 100$$

Note: Combination of these results implies a pH stability in a pH-range of 3-11.

5.13. Physisorption

As discussed in the main text, CO_2 adsorption at 195 K provided a more reliable assessment of the accessible porosity of TUW-11 compared to N_2 adsorption at 77 K. However, CO_2 adsorption may also be subject to diffusion limitations in materials with even narrower pore apertures approaching the kinetic diameter of CO_2, potentially leading to an underestimation of the accessible porosity. For the sake of completion, further isotherms (N_2, H_2O and CO_2 at 77, 298 and 298 K, respectively) were also measured and the data are shown below.

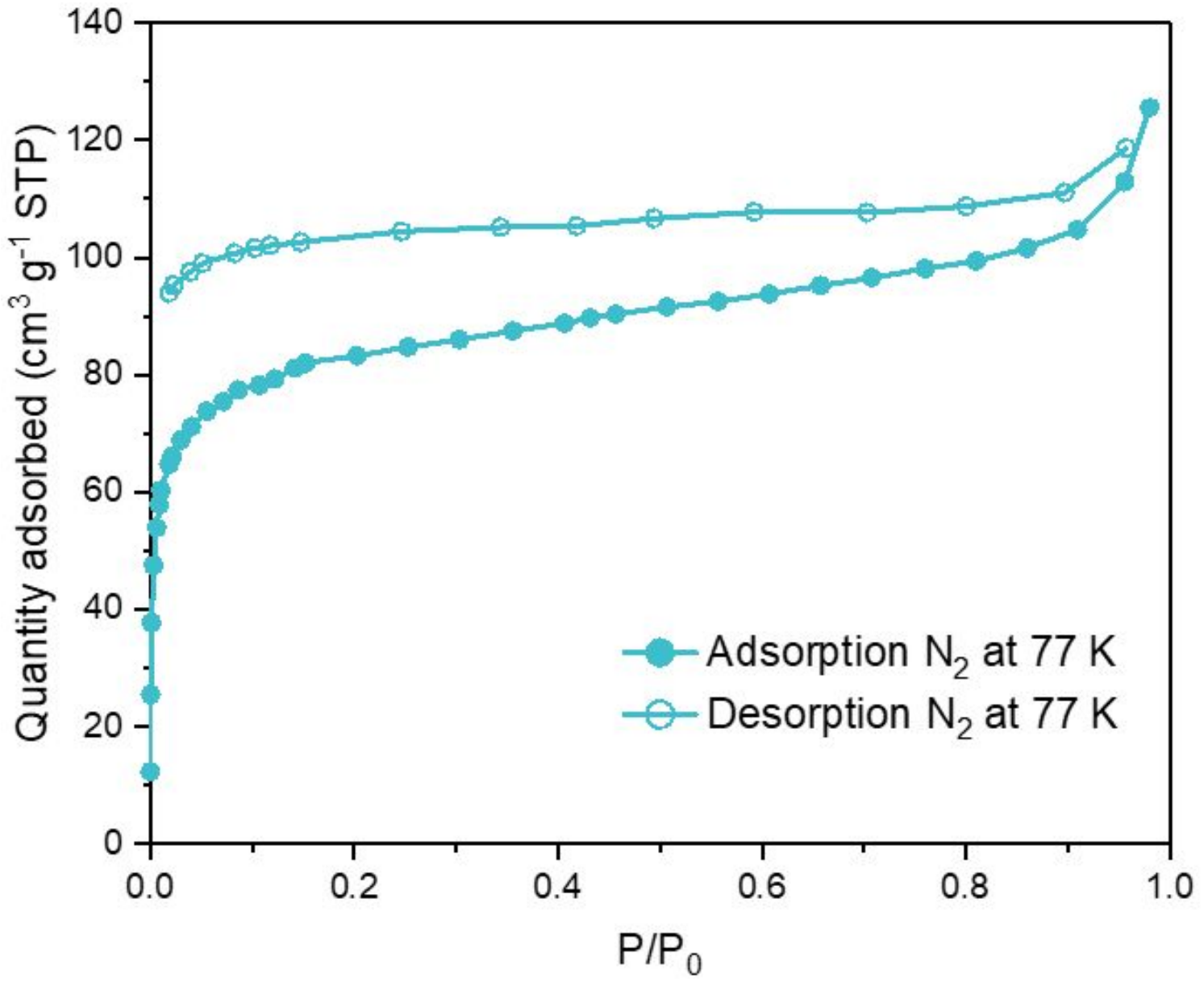

Figure S47. N_2 physisorption isotherms at 77 K.

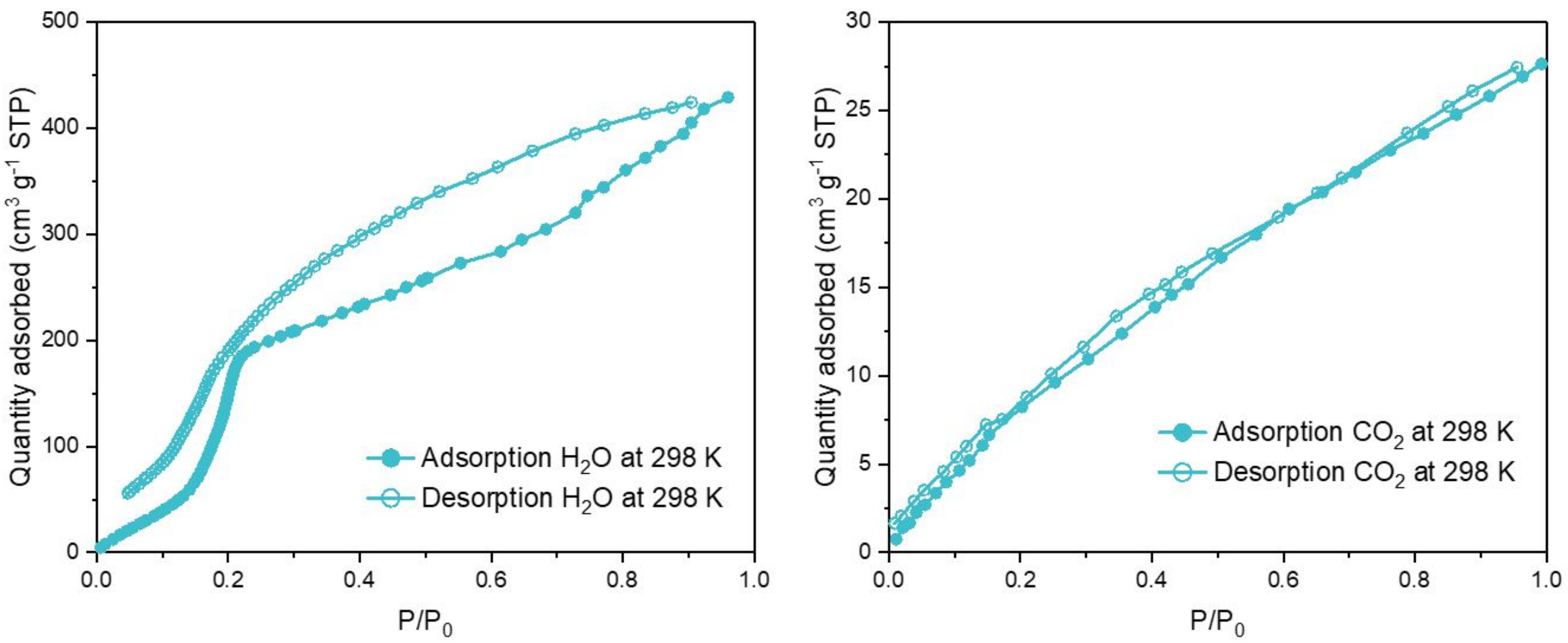

Figure S48. H_2O and CO_2 physisorption isotherms at room temperature (298 K).

Calculated BET surface area

To provide a reference point for surface area and pore volume, we analyzed the CIF-file of the idealized structure model of TUW-11 using Poreblazer. The calculated surface area for N_2 at 77 K (closest

standard reference to the CO_2/195 K experiment) is approximately 920 m^2 g^{-1} and the network-accessible pore volume is 0.38 cm^3 g^{-1}, in good agreement with the experimentally determined pore volume of 0.36 cm^3 g^{-1}.

5.14. UV-vis absorption of reference materials

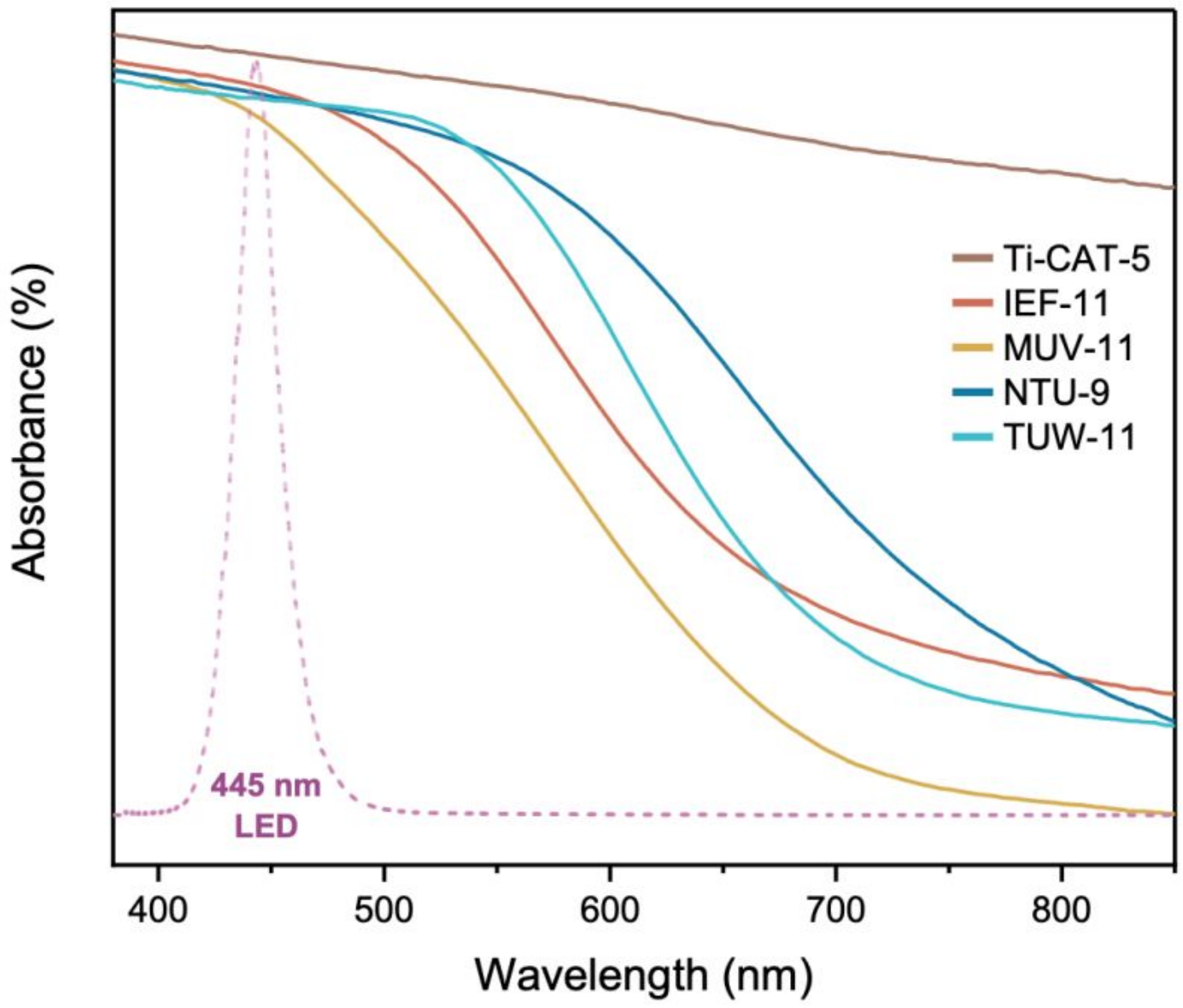

Figure S49. DRS spectra of all prepared phenolate-based MOFs overlapped with the spectral flux of the LED lamp (445 nm) used for excitation during dye-removal experiments.

Table S7. Resulting optical band gaps using ½ or 2 as an exponent for the Tauc functions for TUW-11 and prepared reference materials as well as the reported values in literature. Ti-CAT-5 lacks an absorption edge and hence, the tangent method could not be implemented.

MOF Material	Tauc Fn $[F(R)h\nu]^{½}$	Tauc Fn $[F(R)h\nu]^{2}$	Reported
**TUW-11**	**1.68**	**1.99**	-
**NTU-9**	1.67	1.94	1.72 [24]
**Ti-CAT-5**	-	-	-
**IEF-11**	1.88	2.26	2.45 [10]
**MUV-11**	1.90	2.42	2.01 [12]

5.15. Electrochemistry

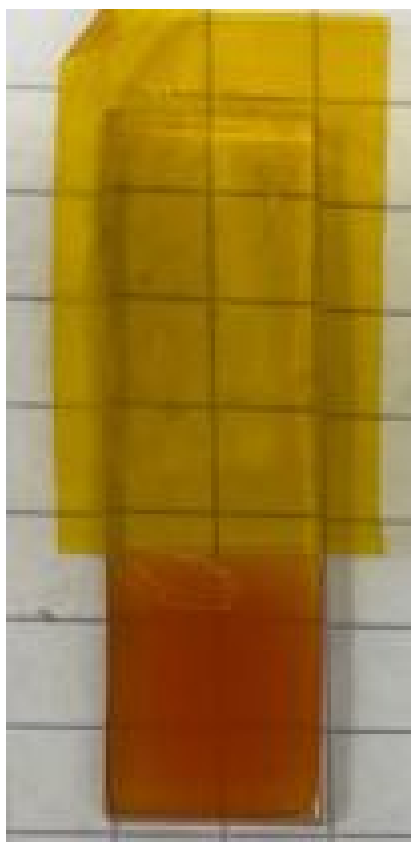

Figure S50. Digital image of the TUW-11 drop-casted FTO covered glass slides used as working electrodes.

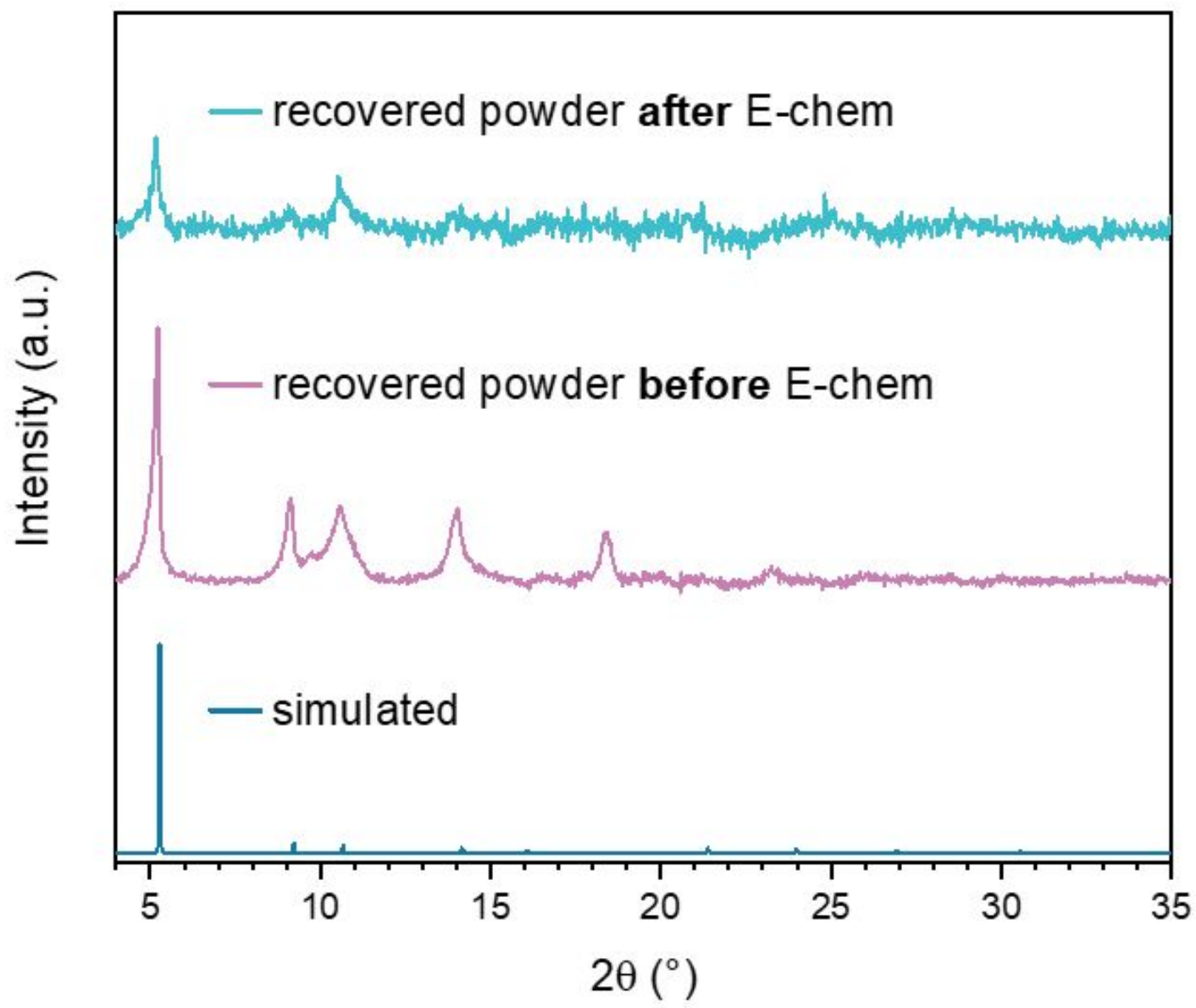

Figure S51. Capillary PXRD of scratched-off powder from the FTO-electrode before and after electrochemistry measurements. The total amount of recoverable TUW-11 is 0.15 mg (see electrode preparation for details).

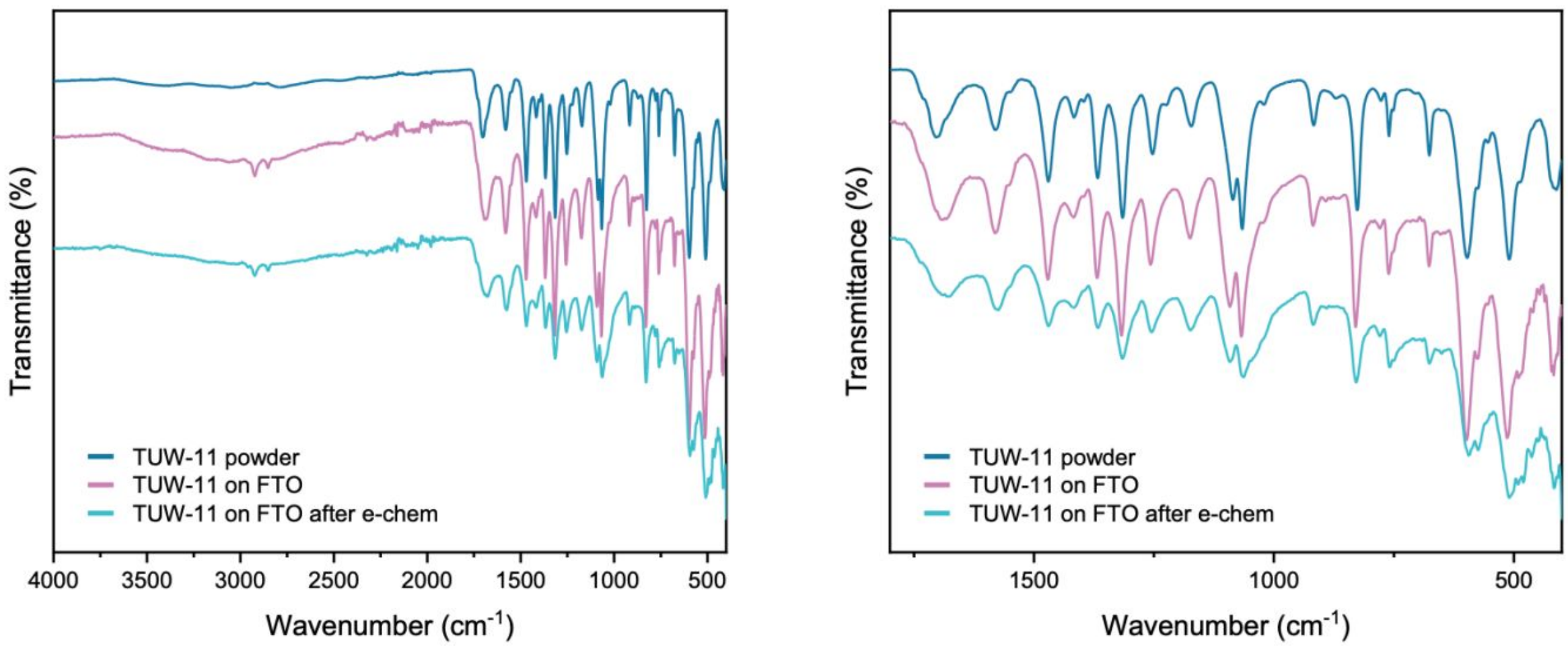

Figure S52. FTIR of TUW-11 on the electrode before and after electrochemistry measurements

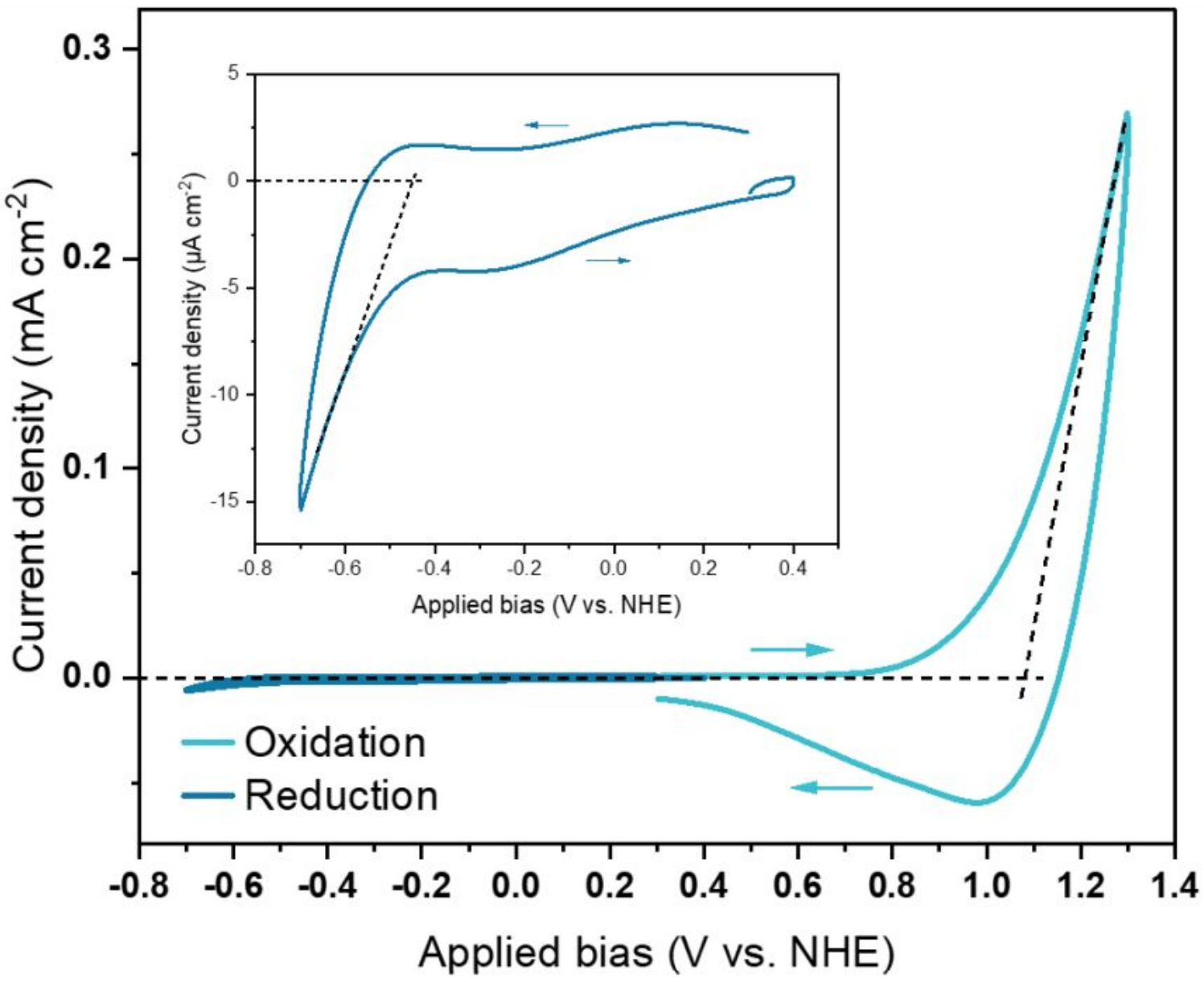

Figure S53. Cyclovoltammetry measurements of TUW-11 coated FTO electrodes to determine electronic band gap and band alignment.

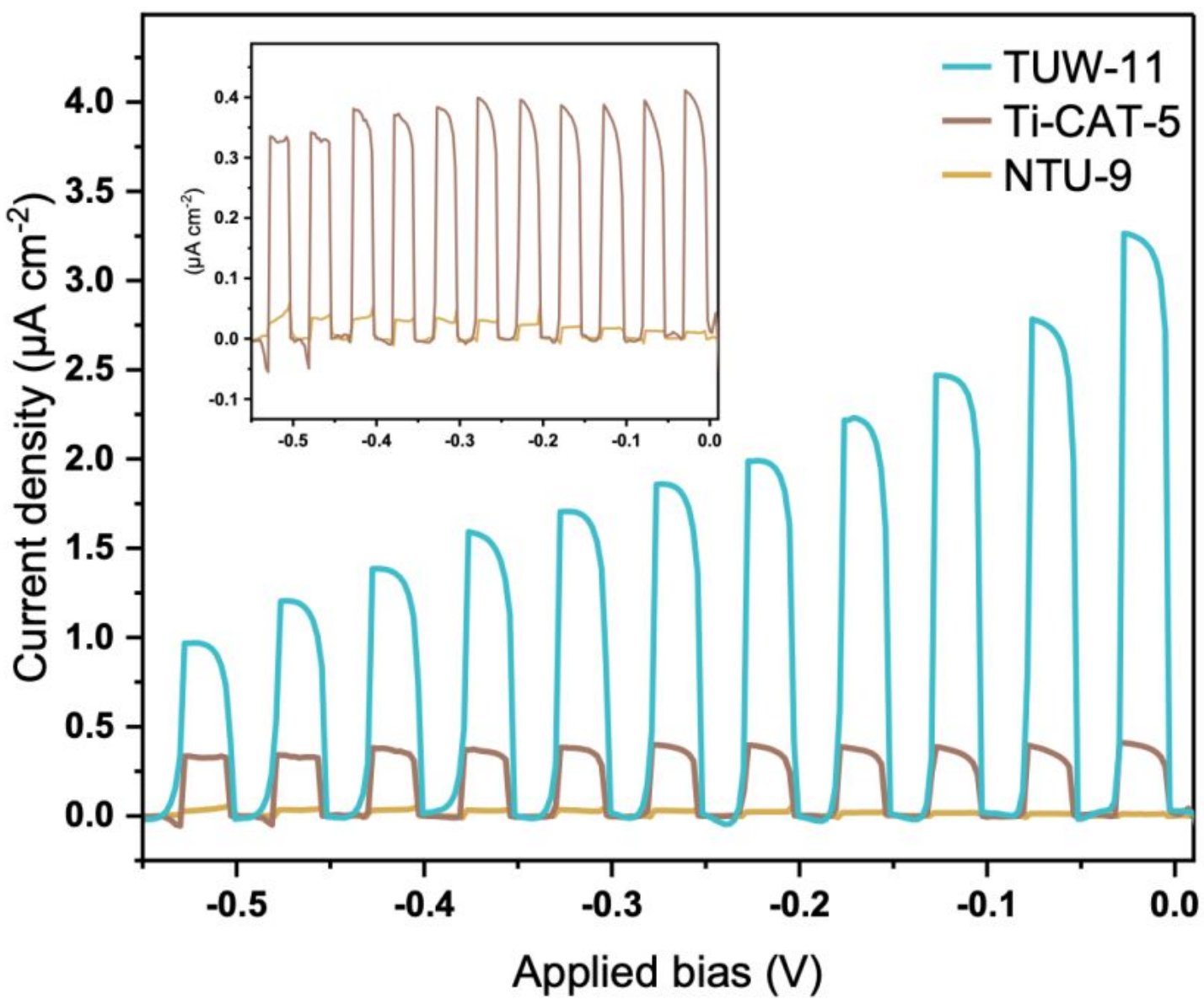

Figure S54. Chopped light linear sweep voltammetry measurements of TUW-11 (light blue) Ti-CAT-5 (brown) and NTU-9 (yellow) coated FTOs showing these reference materials' photo-response under negative bias.

Table S8. Photocurrents at different applied potentials (0 and -0.5 V) for TUW-11 and other reference materials.

MOF Material	Photocurrent at 0 V (μA cm^{-2})	Photocurrent at -0.5 V (μA cm^{-2})	Source
**TUW-11**	**3.20** *	**0.96** *	* This work
**NTU-9**	0.01 *<br>0.06 [24]	0.06 *	* This work, [24]
**Ti-CAT-5**	0.39 *	0.33 *	* This work
**IEF-11**	0.06 [25]	0.60 [10]**	[25], [10]
**MUV-11**	0.60 [12]	0.20 [12]	[12]

** at +1.6 V

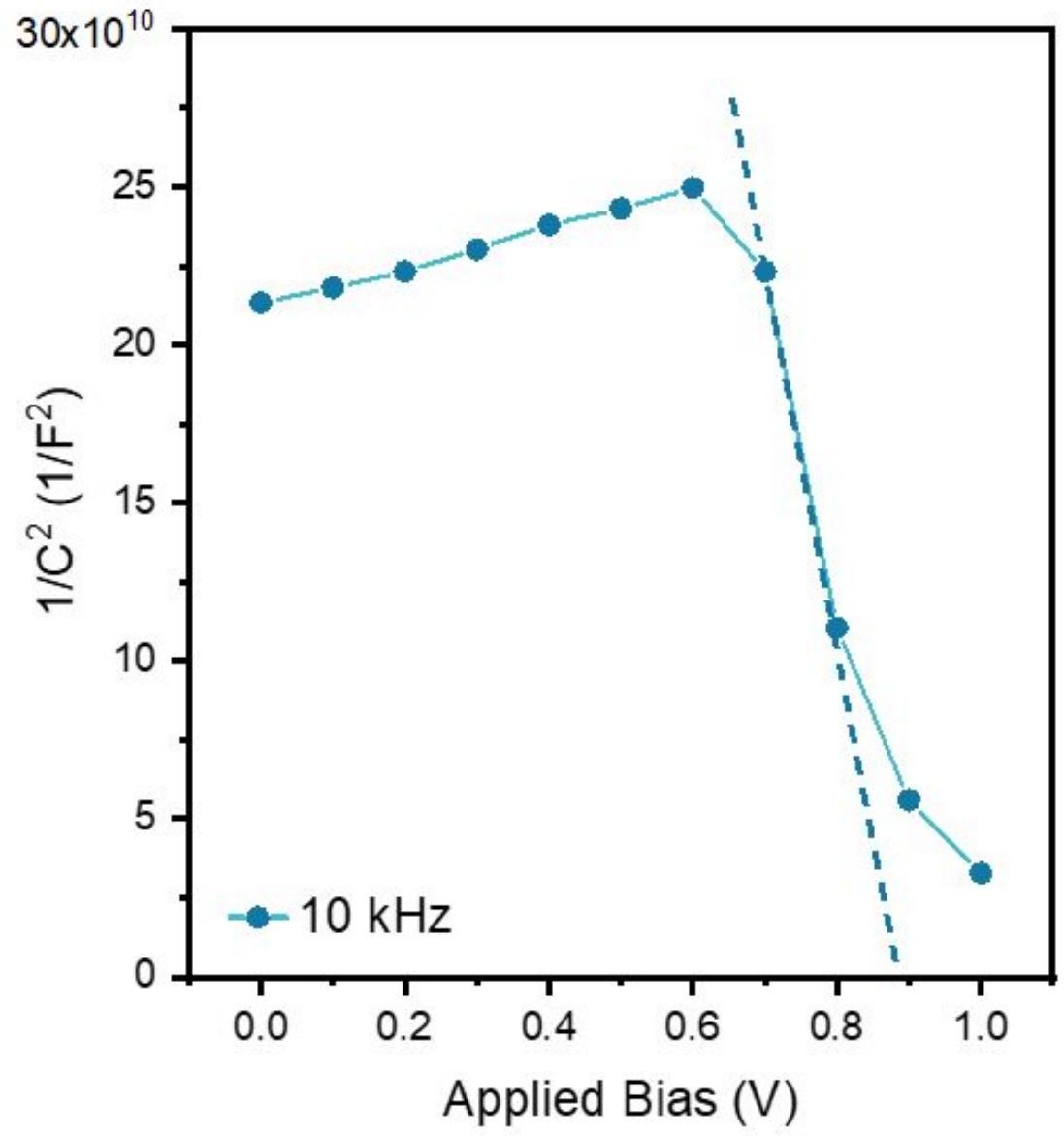

Figure S55. Mott-Schottky plot at 10 kHz with a negative slope (p-type) and a flat band potential of 0.88 V

5.16. Electron paramagnetic resonance (EPR)

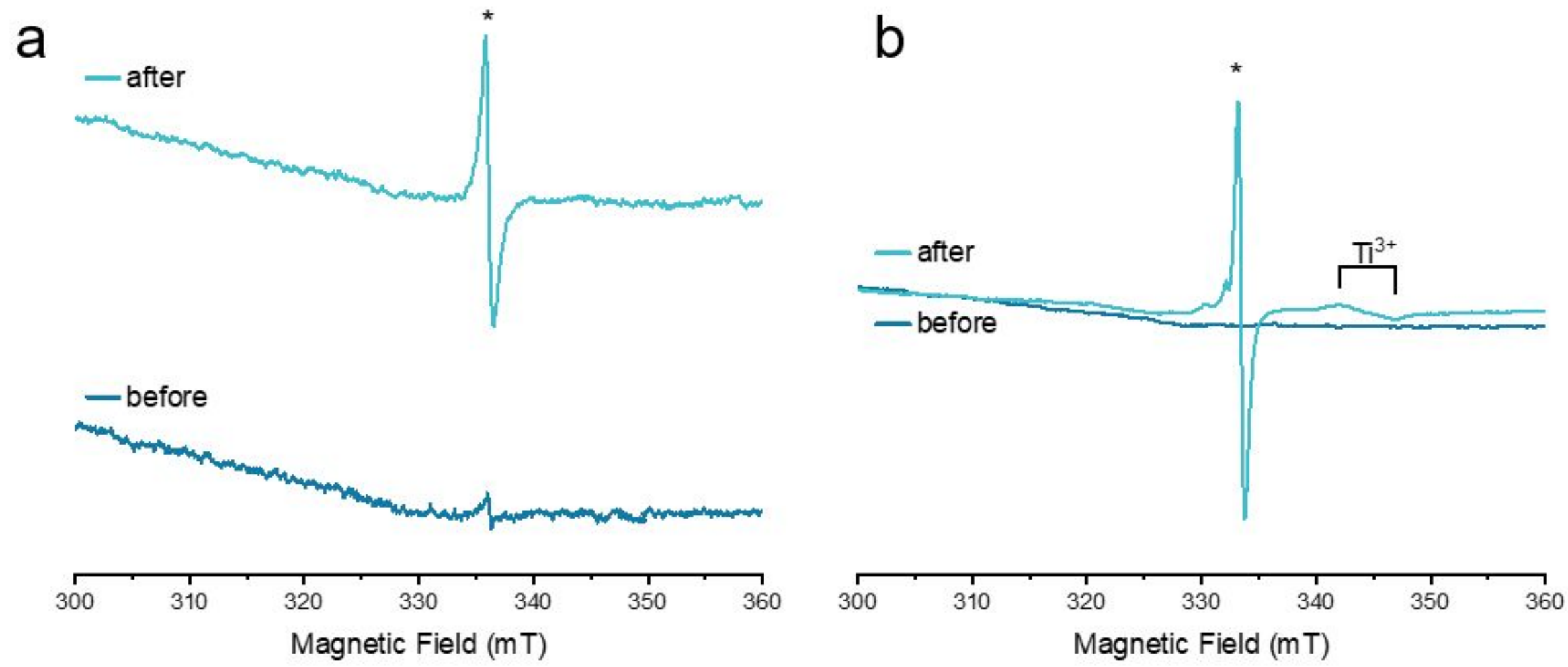

Figure S56. EPR spectra of MOF samples recorded in solid state at 77 K before (dark blue) and after 20 min illumination (light blue) for TUW-11 (**a**) and the reference MIL-125 (**b**). The signal corresponding to oxygen-trapped holes around 338 mT (g-factor of 2.02) is marked with an asterisk. The signal corresponding to the formation of Ti^{3+} centered at 344 mT (g-factor of 1.95) could only be observed in the MIL-125 sample, which was accompanied with the characteristic color change (white to grey/black), absent in TUW-11.

5.17. Photoluminescence (PL) and time-resolved emission spectroscopy (TRES)

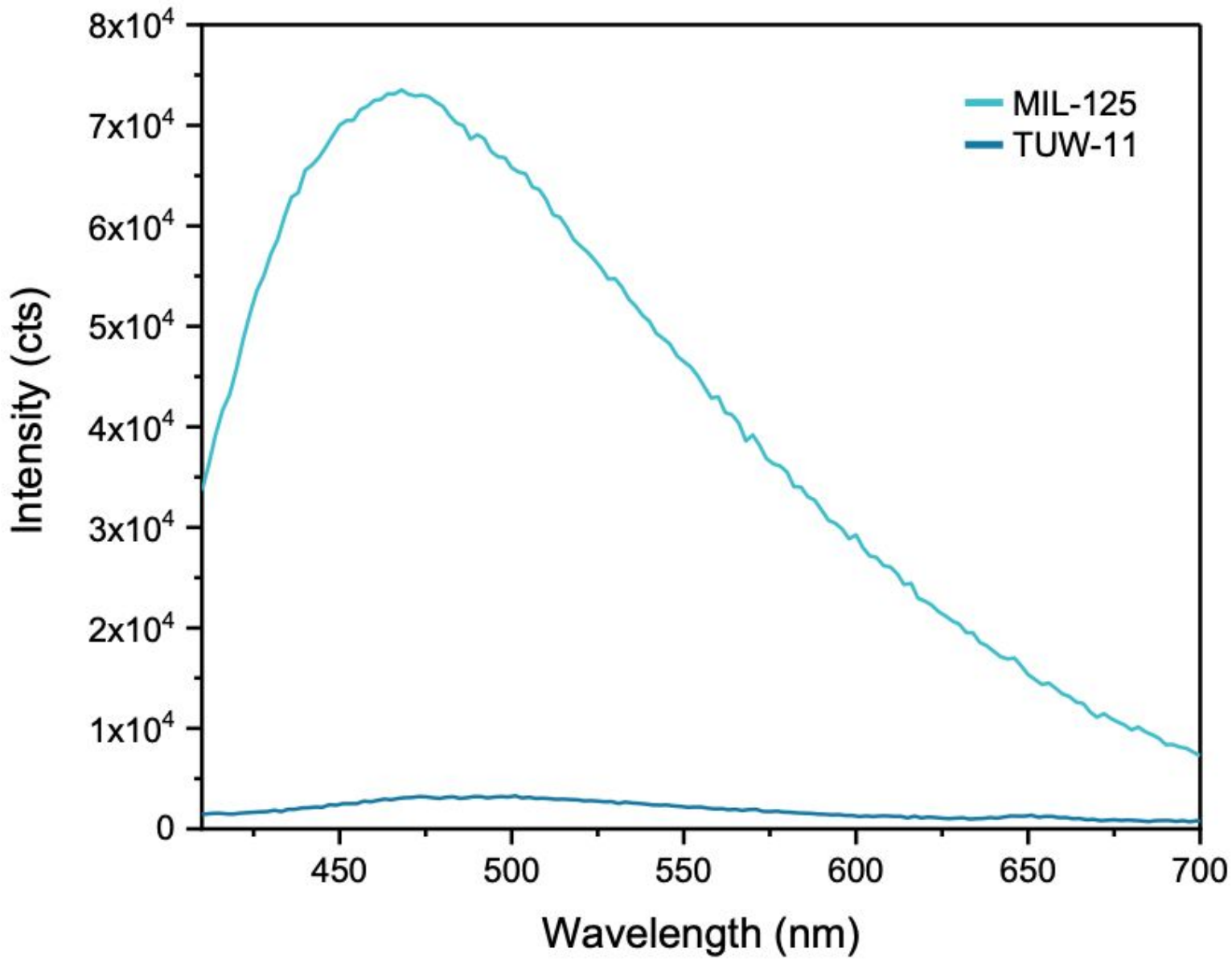

Figure S57. Photoluminescence (PL) emission spectra of MIL-125 (light blue) *vs*. TUW-11 (dark blue). Excitation λ = 378 nm.

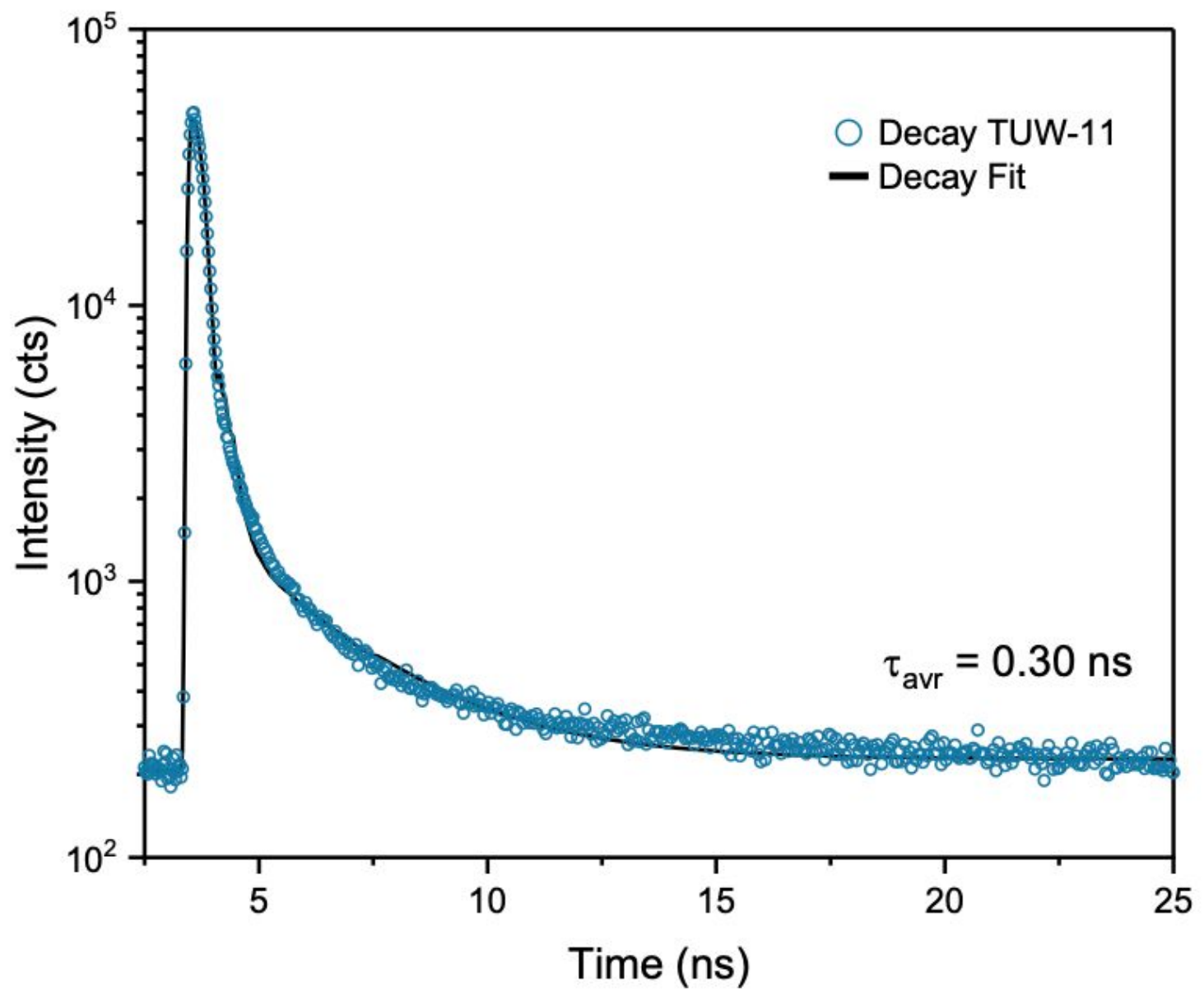

Figure S58. Time-resolved PL emission spectra for TUW-11 under 377 nm laser excitation**.**

5.18. Density of states (DOS)

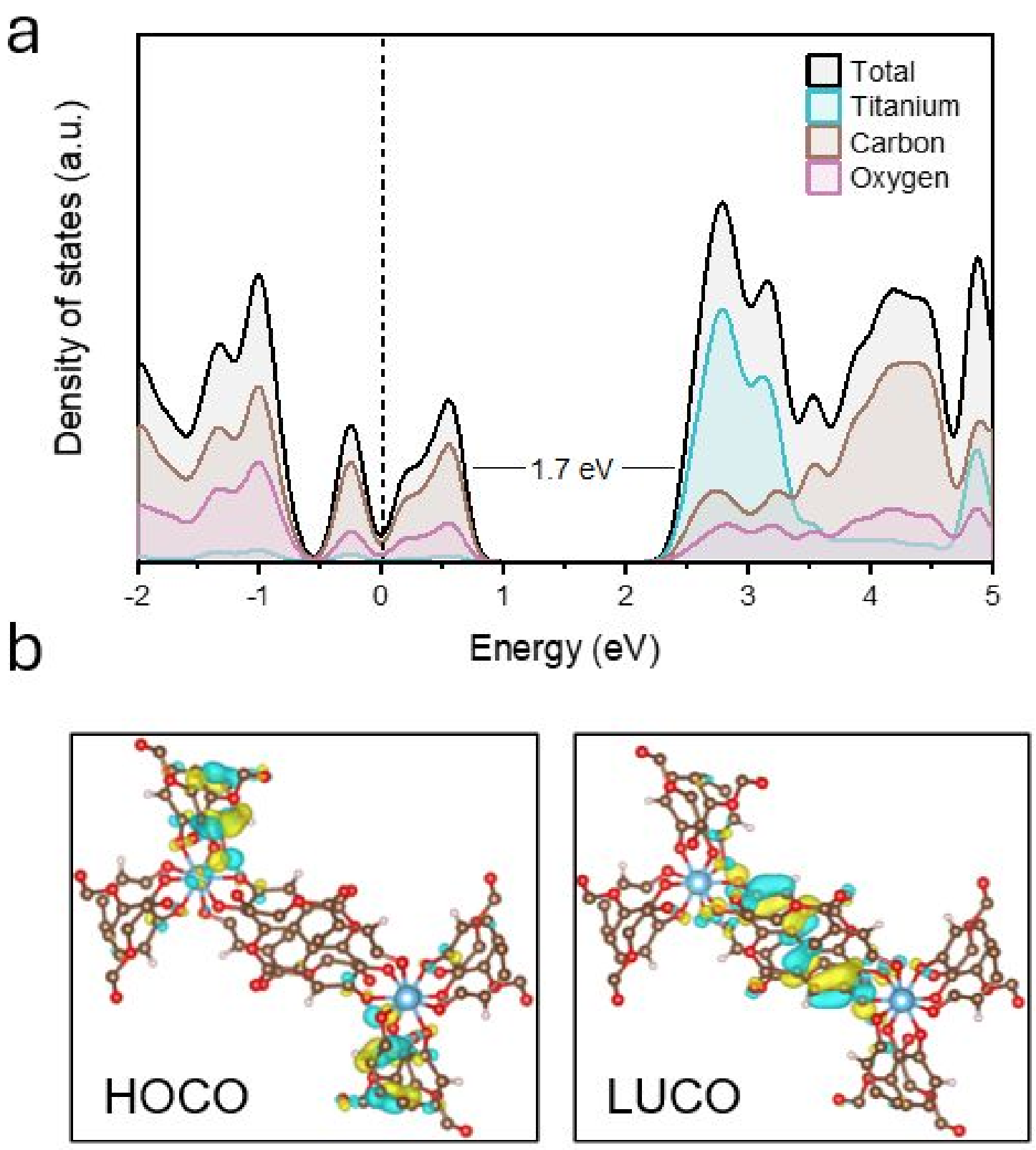

Figure S59. (a) Total (black) and projected (titanium, blue; carbon, brown; oxygen, pink) density of states for TUW-11. **(b)** Examples of isosurfaces (isosurface value 0.04 e Å^{-3}) of Kohn-Sham orbitals below (left) and above (right) the band gap in (a).

Table S9. Ti-O bond lengths obtained from the DFT-relaxed structure model of TUW-11 (see computational methods for details) and comparison with the average Ti-O distance determined experimentally by EXAFS.

Method	Ti-O distances (Å)	Average Ti-O distance (Å)
DFT-relaxed structure model	1.89	1.98 ± 0.02
	1.92	
	1.95	
	1.96	
	2.05	
	2.09	
EXAFS	1.98	2.00 ± 0.01
	2.01	

5.18. Point of zero charge (PZC) and zeta potential

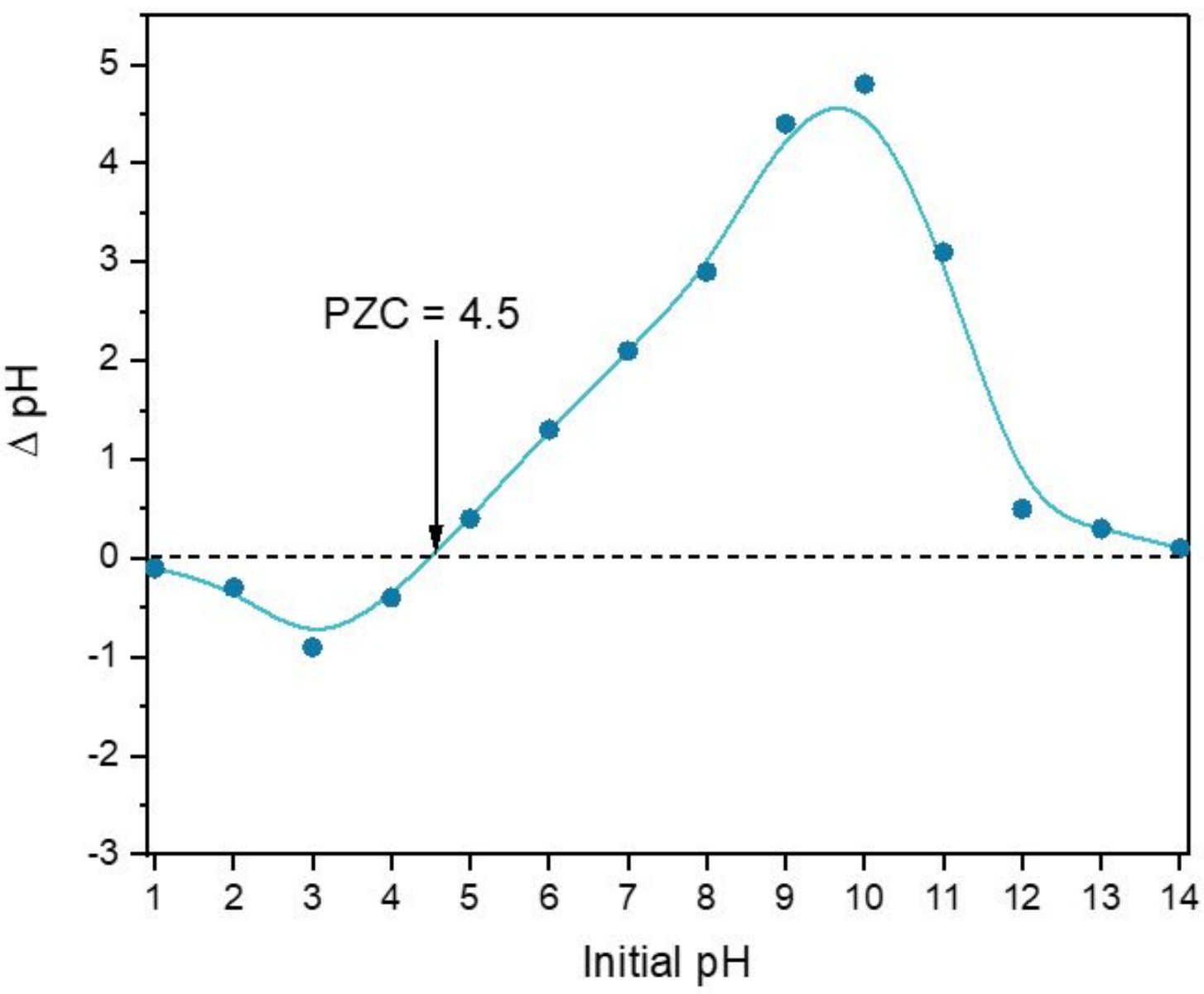

Figure S60. Point of zero charge (PZC) curve by plotting initial pH *vs.* difference of pH before and after dispersing TUW-11 at different pH. Details in **Table S6**.

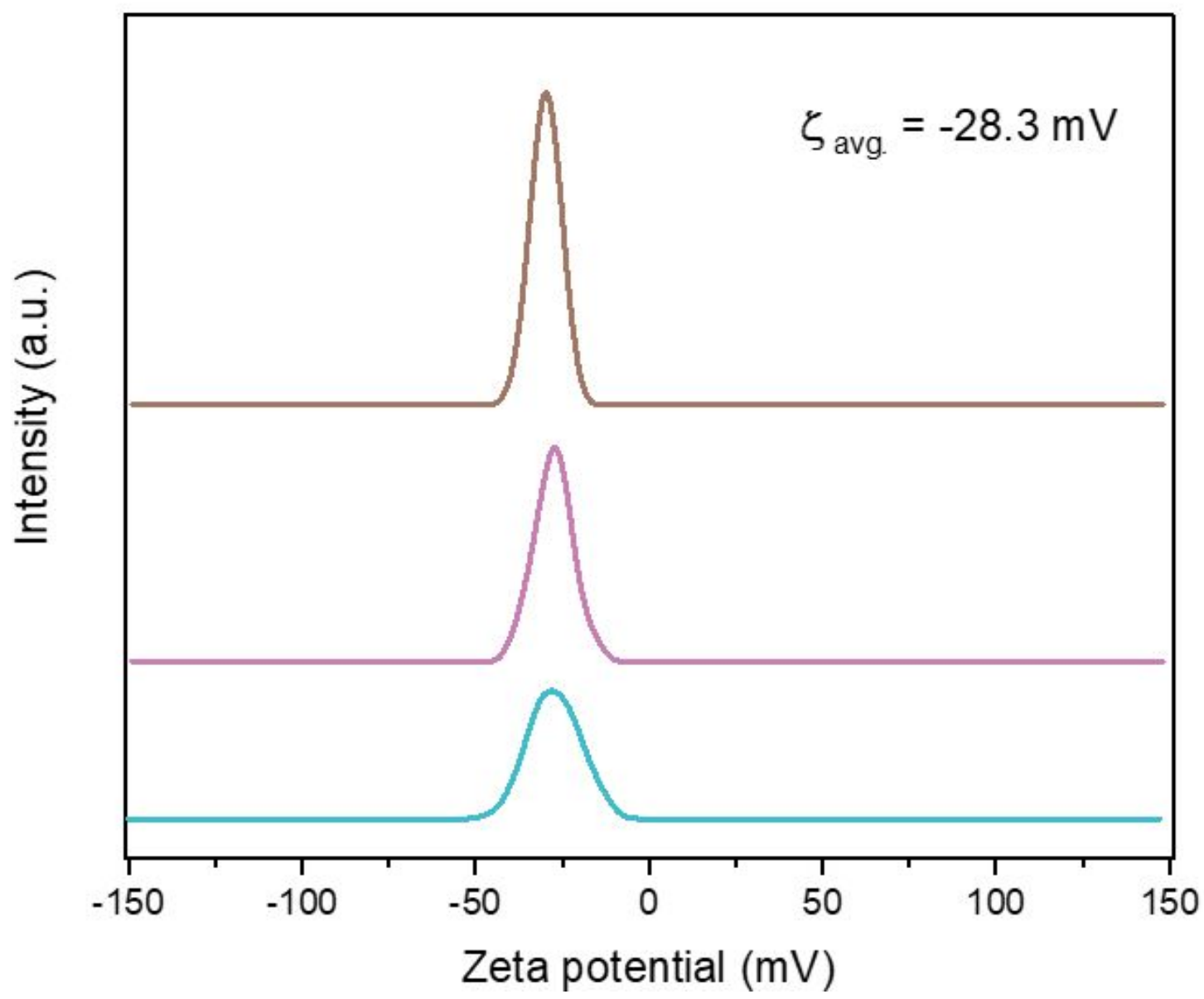

Figure S61. Zeta-Potential Distribution of three different batches, all showing an average zeta potential of -28.3 mV in water at pH 6.5.

S6. Selective adsorption and linker-centered photoconversion

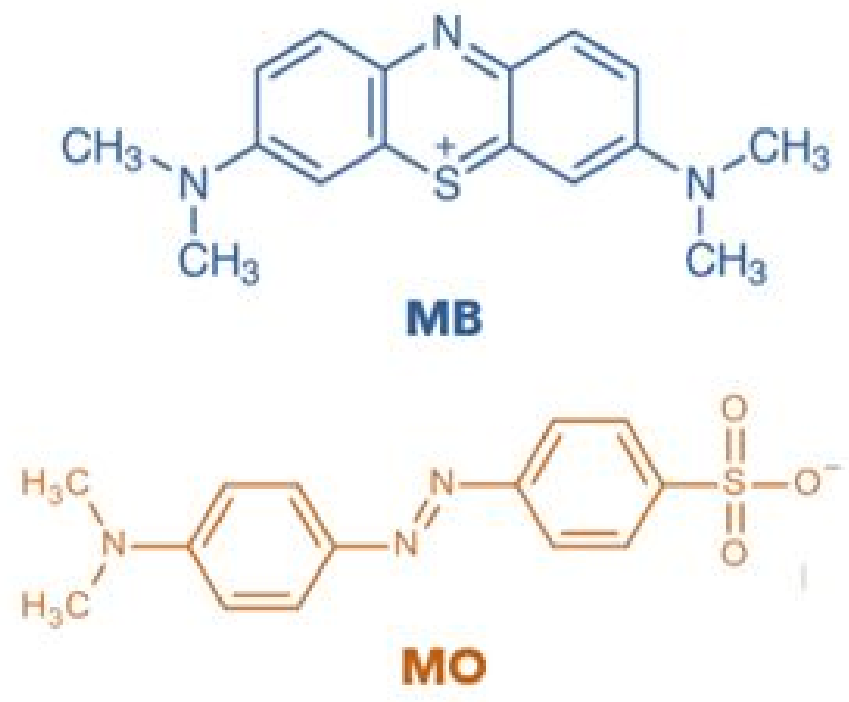

Figure S62. Chemical structures of the differently charged organic dye molecules used in this study: Methylene Blue (MB), a cationic dye and Methyl Orange (MO), an anionic dye.

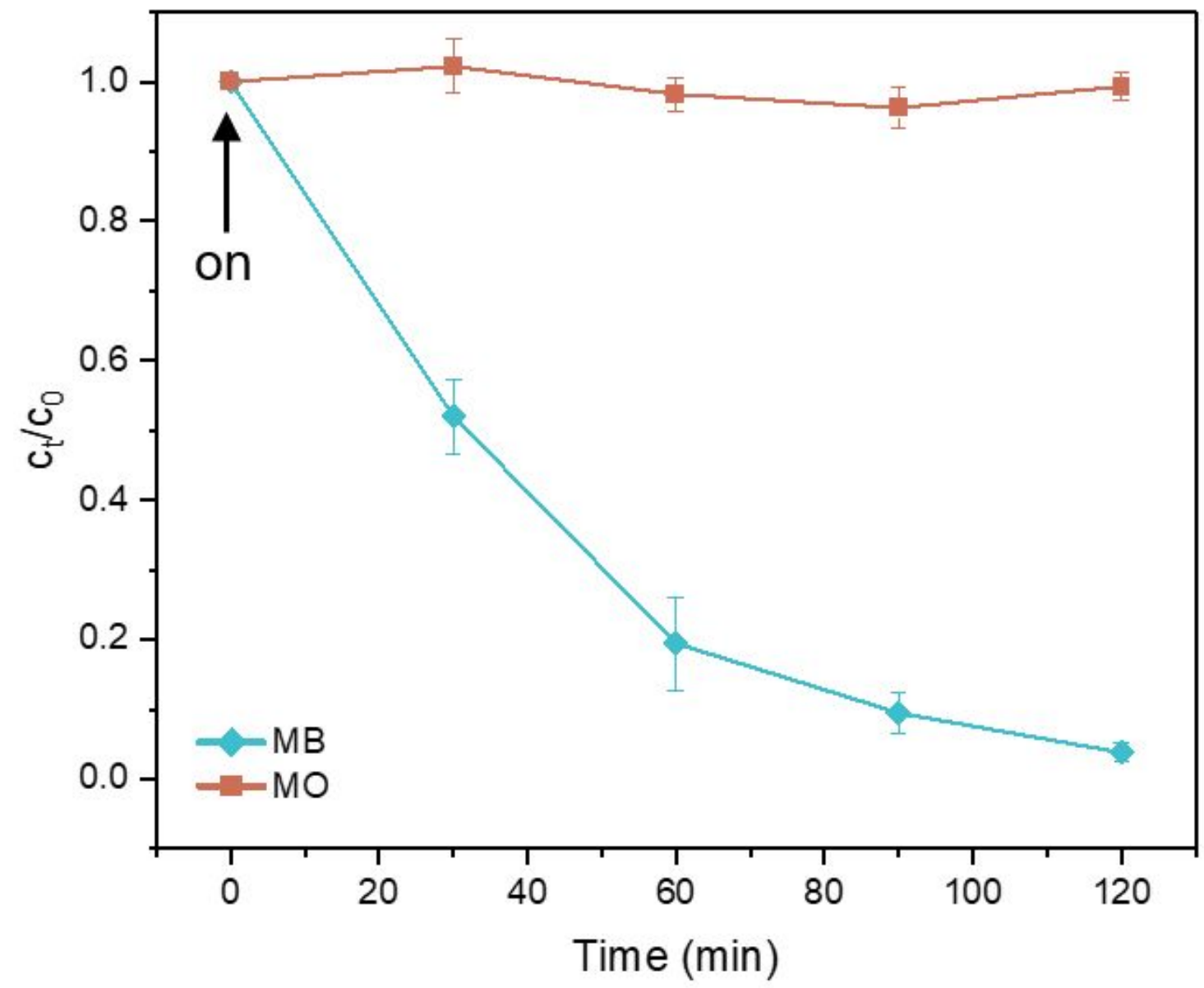

Figure S63. Methylene blue (MB) and Methyl orange (MO) light-assisted removal after 2 h illumination in the presence of TUW-11.

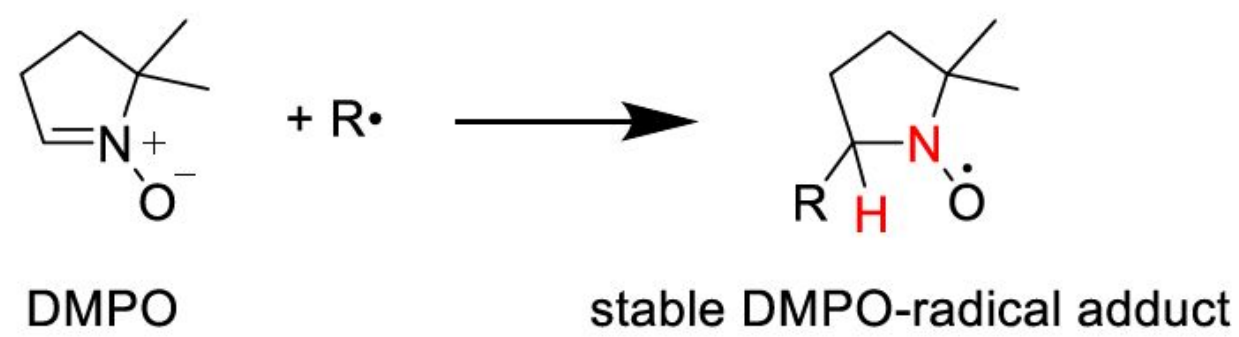

Figure S64. Spin trapping by DMPO, leading to a stable, detectable radical adduct; the atoms for which hyperfine coupling constants can be detected are marked in red

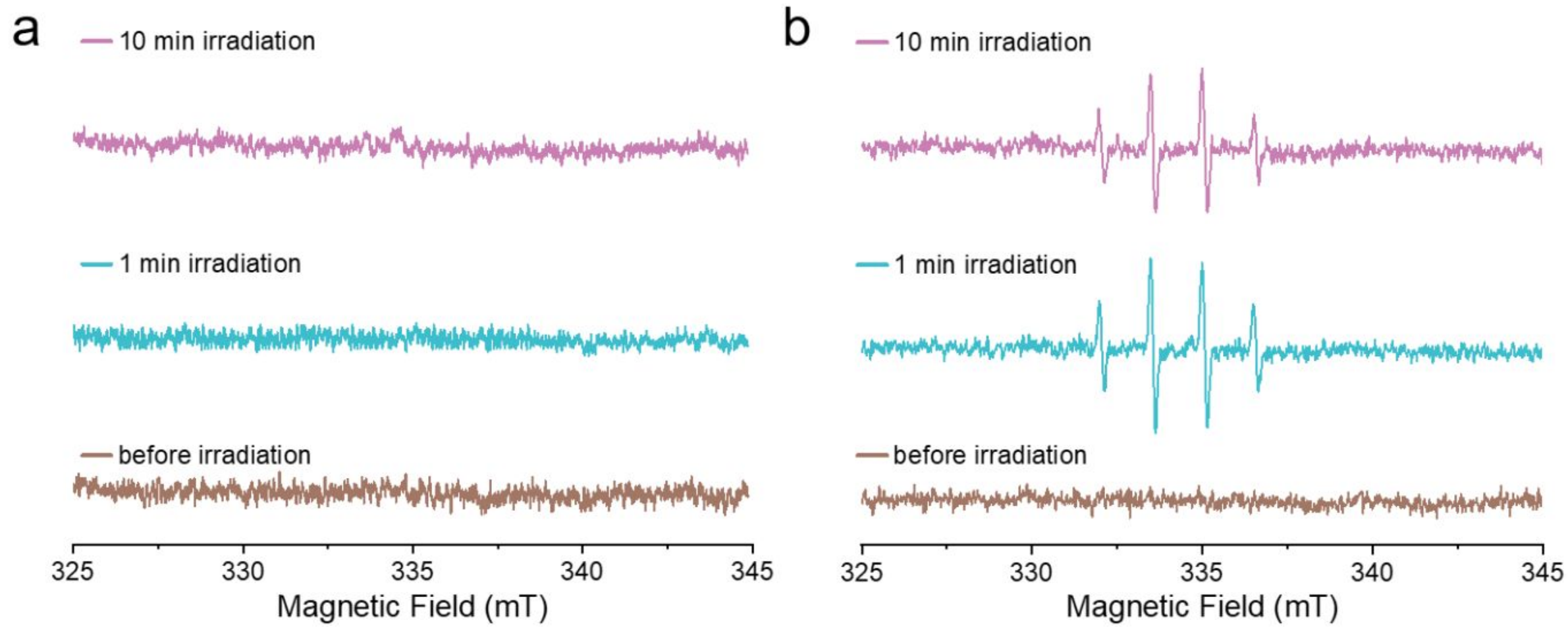

Figure S65. Spin-trapping EPR experiments with DMPO in the presence of TUW-11 (**a**) or MIL-125 (**b**), leading to a stable, detectable radical adduct[26] in the case of MIL-125.

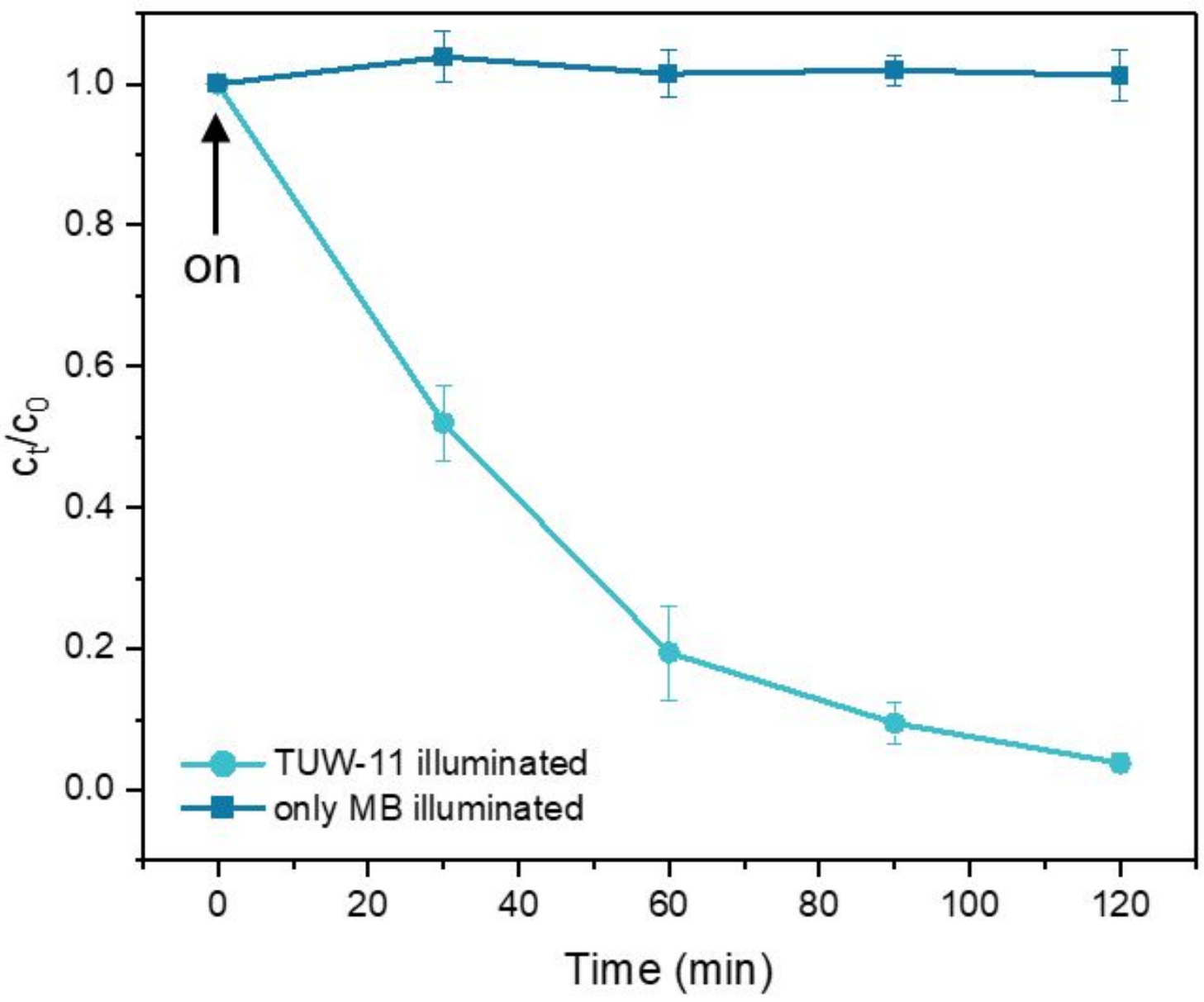

Figure S66. Control experiment: methylene blue (MB) removal after illumination in the absence (dark blue) or presence (light blue) of TUW-11. Error bars resulting from 3 different experiments.

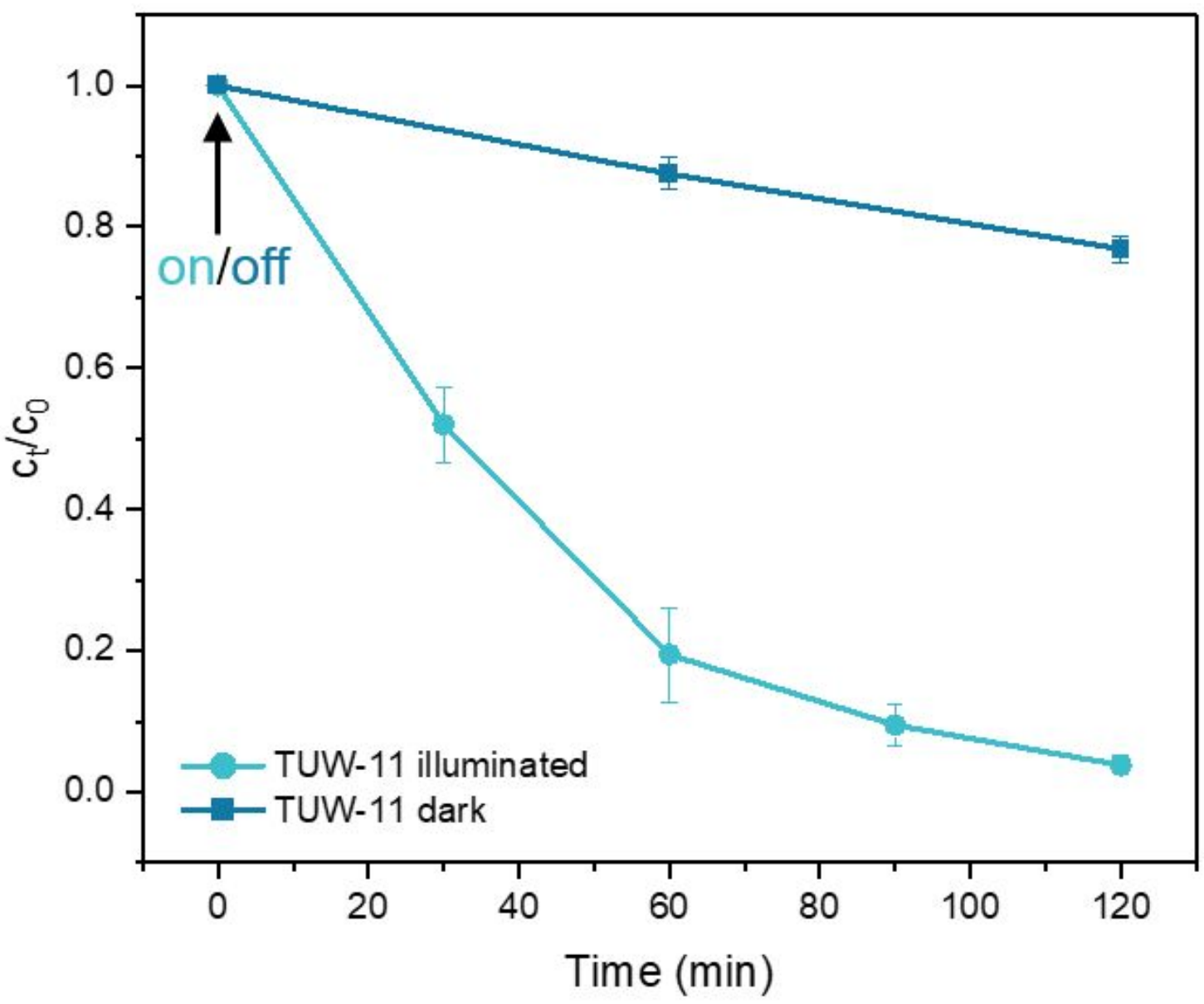

Figure S67. Control experiment: methylene blue (MB) removal with TUW-11 after 2 hours stirring in the dark (dark blue) *vs.* under illumination (light blue). Error bars resulting from 3 different experiments.

Table S10. Quantitative comparison of total MB removal by TUW-11 (4 h equilibration + 2 h under dark or illuminated conditions) in **Figure S67**. The values are calculated by determining the MB remaining in solution relative to the initial identical MB (1.95 mg) amounts. Uncertainties represent ± 1 standard deviation from three independent experiments. The uncertainty of the light-induced contribution is calculated by the propagation of uncertainty standard formula.

Experiment	MB removed (mg)	MB removal (%)
Dark control	1.742 ± 0.020	89.2
Light-assisted removal	1.946 ± 0.004	99.7
Light-induced contribution	0.204 ± 0.020	10.5

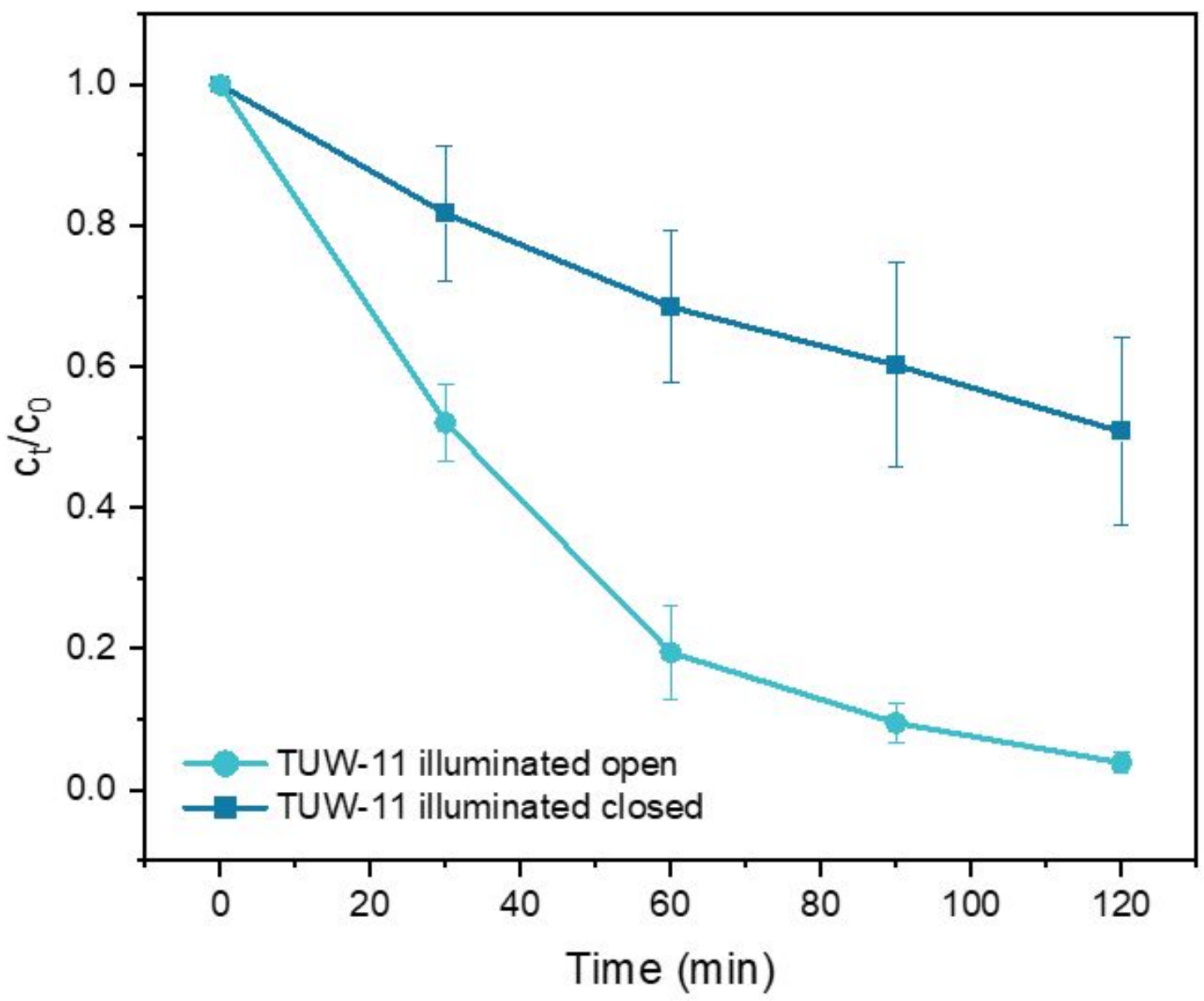

Figure S68. Control experiment: methylene blue (MB) removal with TUW-11 after 2 hours stirring under illumination in an open vessel (light blue) *vs.* in a closed N_2 atmosphere (dark blue), established by bubbling N_2 through the solution for 10 min before starting the experiment. Error bars resulting from 3 different experiments.

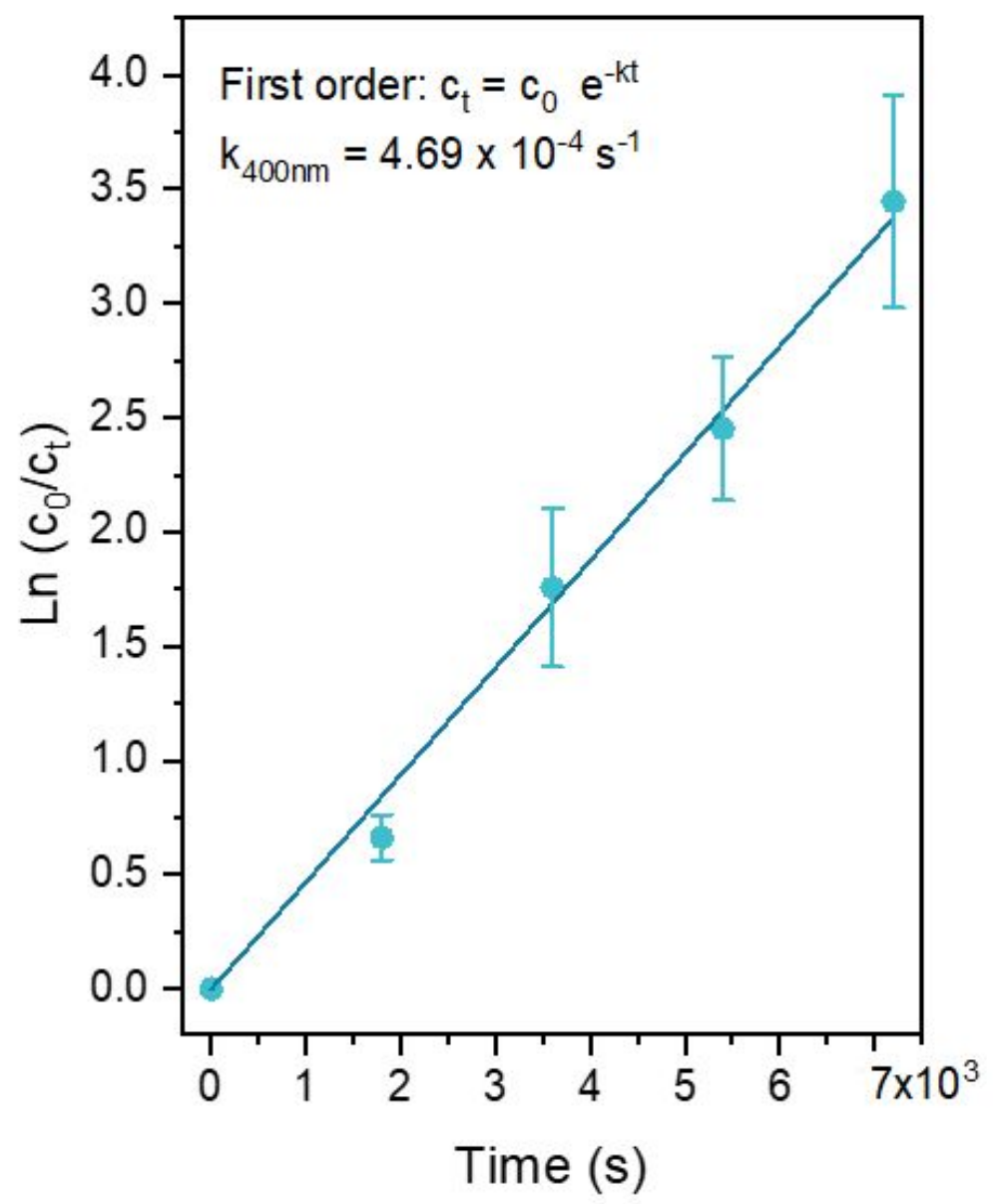

Figure S69. Linear fit of the time profile *vs.* the natural logarithm of the c_0/c_t, showing good agreement ($R^2 > 0.99$) with a first order reaction and extracted reaction rate constant *k* (slope of the curve).

Table S11. Comparative table of reaction rates for the degradation of MB for λ > 420 nm from our previous work using carboxylate-based MOFs and oxides.

Sample	k (x 10^{-4} s^{-1})	Ref.
TUW-11	4.69	This work
COK-47	2.21	[27]
MIL-125	0.18	[27]
P25 TiO_2	0.16	[27]

6.1. Cycling and post-conversion characterization of TUW-11

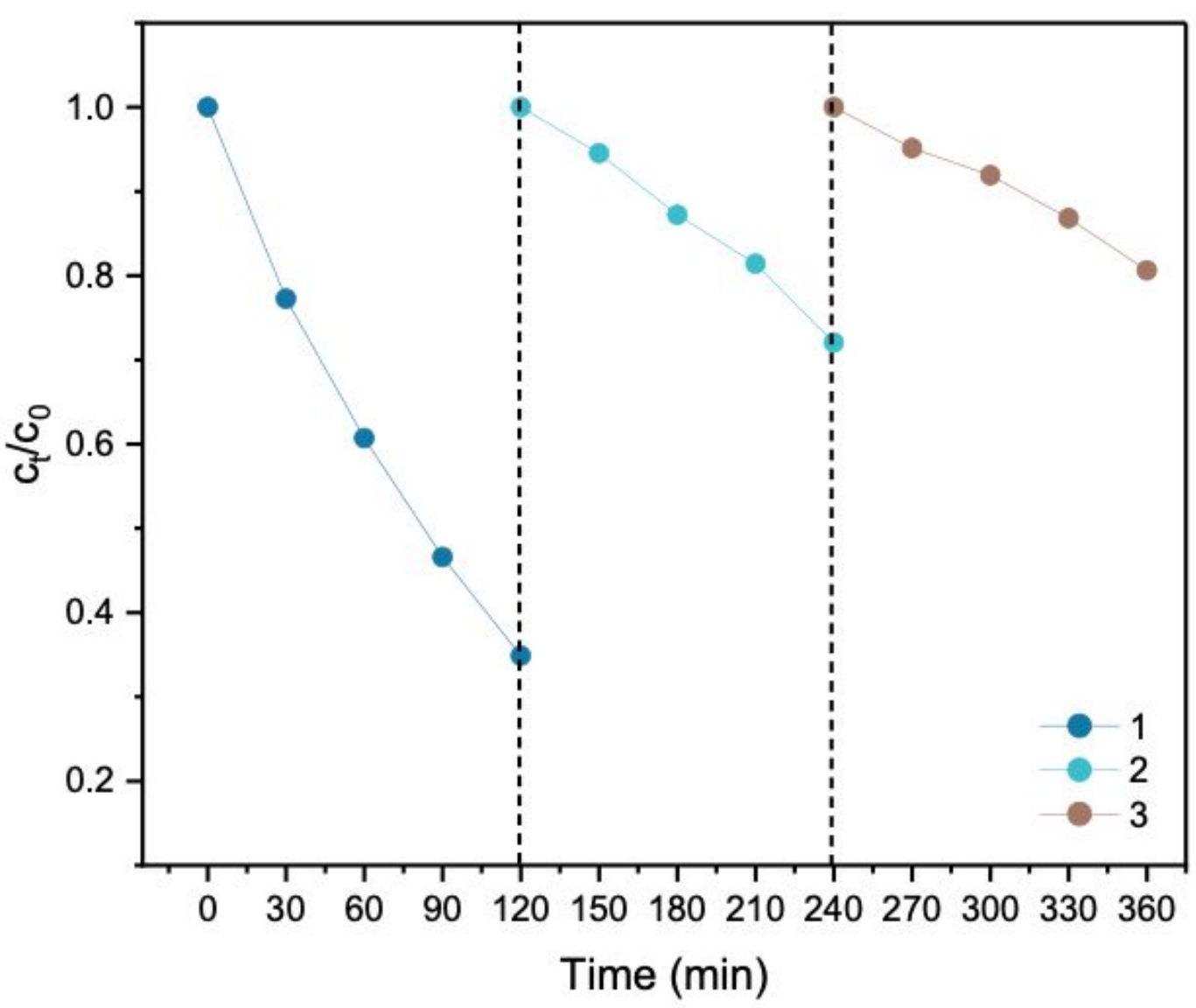

Figure S70. recyclability after recovering TUW-11, washing with DI water, drying and resuspending it in a fresh MB solution. Equilibration time was 2 hours prior to illumination.

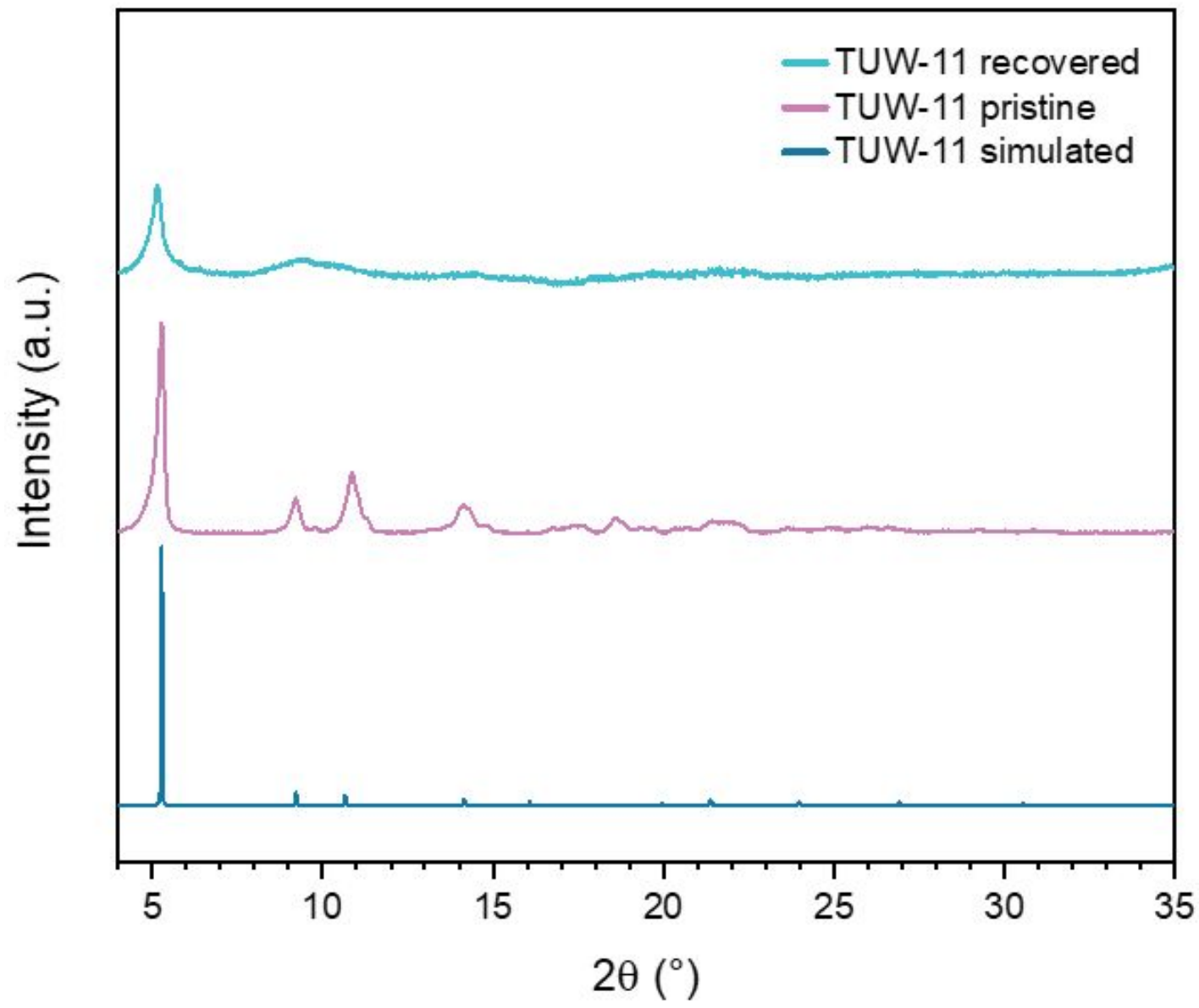

Figure S71. Capillary PXRD patterns measured in a 1.0 mm capillary of the recovered TUW-11 material after 3 dye adsorption and light-assisted removal cycles.

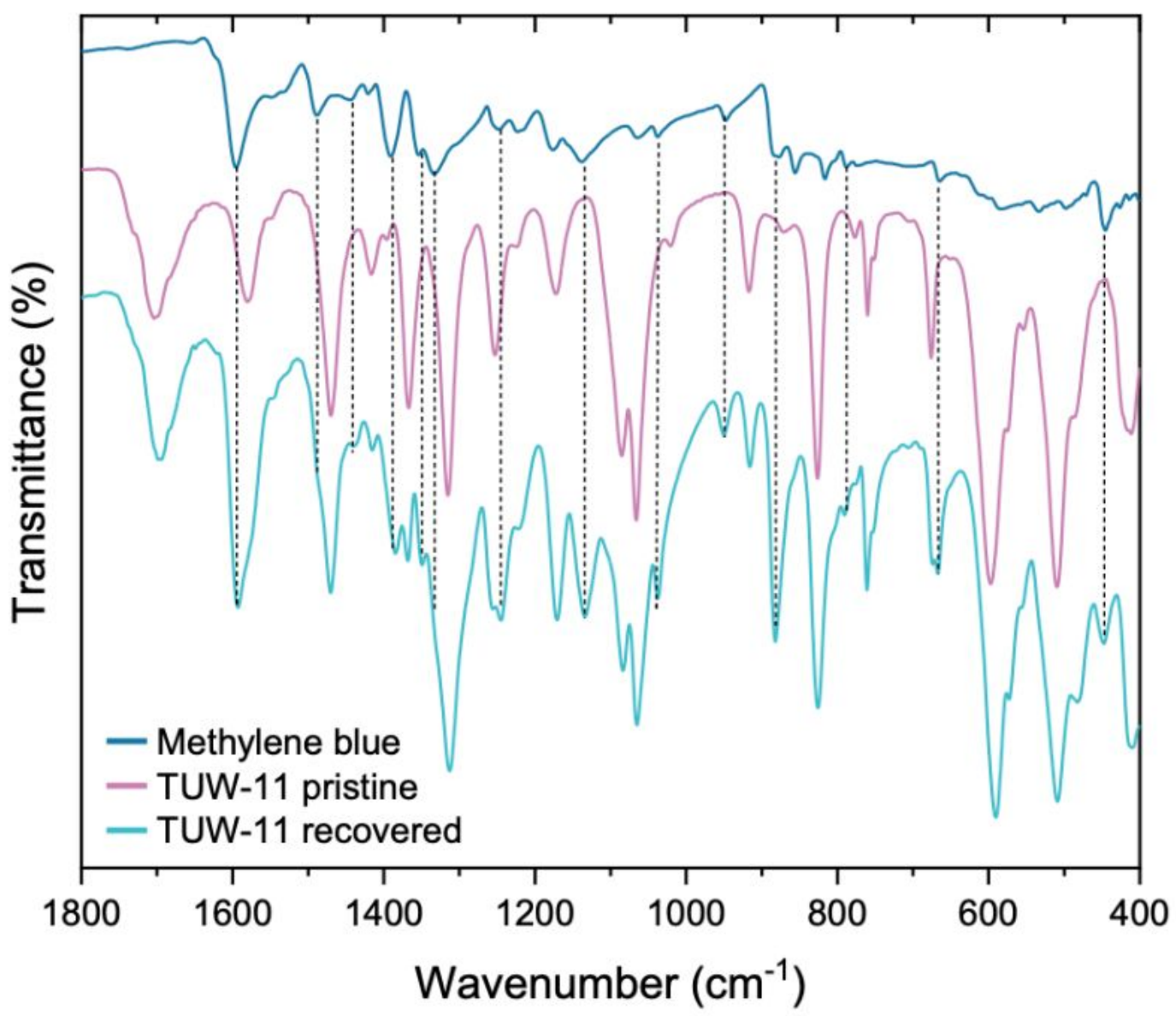

Figure S72. FTIR spectra of the recovered TUW-11 material after 3 dye adsorption and light-assisted removal cycles compared to pristine TUW-11 and pristine MB.

Table S12. Ti concentration found with TXRF after a typical dye degradation experiment and respective Ti leached percentages.

Sampling times	Ti (mg L^{-1})	Ti leached (%)
120 min dark	< 0.038	0.23
30 min light	< 0.034	0.21
90 min light	< 0.053	0.33
120 min light	< 0.037	0.23

S7. References

(1) Cichocka, M. O.; Ångström, J.; Wang, B.; Zou, X.; Smeets, S. High-Throughput Continuous Rotation Electron Diffraction Data Acquisition via Software Automation. *J. Appl. Crystallogr.* **2018**, *51* (6), 1652–1661. https://doi.org/10.1107/S1600576718015145

(2) Wan, W.; Sun, J.; Su, J.; Hovmöller, S.; Zou, X. Three-Dimensional Rotation Electron Diffraction: Software RED for Automated Data Collection and Data Processing. *J. Appl. Crystallogr.* **2013**, *46* (6), 1863–1873. https://doi.org/10.1107/S0021889813027714

(3) Chinchilla-Garzón, C.; Rivera-Chao, E.; Gomez-Mendoza, M.; Vieira, B. J. C.; Almora-Barrios, N.; Gimeno-Fonquernie, P.; Castells-Gil, J.; Vismara, R.; Navrátil, J.; Błoński, P.; O'Shea, V. A. de la P.; Waerenborgh, J. C.; Martí-Gastaldo, C.; Padial, N. M. Cluster-Enabled Control of Single-Electron Photoredox Catalysis in Reticular Frameworks. *Chem* **2026**, *12* (9). https://doi.org/10.1016/j.chempr.2026.103074

(4) Blum, V.; Gehrke, R.; Hanke, F.; Havu, P.; Havu, V.; Ren, X.; Reuter, K.; Scheffler, M. *Ab Initio* Molecular Simulations with Numeric Atom-Centered Orbitals. *Comput. Phys. Commun.* **2009**, *180* (11), 2175–2196. https://doi.org/10.1016/j.cpc.2009.06.022

(5) Adamo, C.; Barone, V. Toward Reliable Density Functional Methods without Adjustable Parameters: The PBE0 Model. *J. Chem. Phys.* **1999**, *110* (13), 6158–6170. https://doi.org/10.1063/1.478522

(6) Coulter, J. B.; Birnie III, D. P. Assessing Tauc Plot Slope Quantification: ZnO Thin Films as a Model System. *Phys. Status Solidi B* **2018**, *255* (3), 1700393. https://doi.org/10.1002/pssb.201700393

(7) Makuła, P.; Pacia, M.; Macyk, W. How To Correctly Determine the Band Gap Energy of Modified Semiconductor Photocatalysts Based on UV–Vis Spectra. *J. Phys. Chem. Lett.* **2018**, *9* (23), 6814–6817. https://doi.org/10.1021/acs.jpclett.8b02892

(8) Fabrizio, K.; Le, K. N.; Andreeva, A. B.; Hendon, C. H.; Brozek, C. K. Determining Optical Band Gaps of MOFs. *ACS Mater. Lett.* **2022**, *4* (3), 457–463. https://doi.org/10.1021/acsmaterialslett.1c00836

(9) Nguyen, N. T. T.; Furukawa, H.; Gándara, F.; Trickett, C. A.; Jeong, H. M.; Cordova, K. E.; Yaghi, O. M. Three-Dimensional Metal-Catecholate Frameworks and Their Ultrahigh Proton Conductivity. *J. Am. Chem. Soc.* **2015**, *137* (49), 15394–15397. https://doi.org/10.1021/jacs.5b10999

(10) Salcedo-Abraira, P.; Babaryk, A. A.; Montero-Lanzuela, E.; Contreras-Almengor, O. R.; Cabrero-Antonino, M.; Grape, E. S.; Willhammar, T.; Navalón, S.; Elkäim, E.; García, H.; Horcajada, P. A Novel Porous Ti-Squarate as Efficient Photocatalyst in the Overall Water Splitting Reaction under Simulated Sunlight Irradiation. *Adv. Mater.* **2021**, *33* (52), 2106627. https://doi.org/10.1002/adma.202106627

(11) Yu, S.; Xiao, Y.; Liu, Z.; Lyu, J.-M.; Wang, Y.-L.; Hu, Z.-Y.; Li, Y.; Sun, M.-H.; Chen, L.-H.; Su, B.-L. Ti-MOF Single-Crystals Featuring an Intracrystal Macro–Microporous Hierarchy for Catalytic Oxidative Desulfurization. *Chem. Commun.* **2023**, *59* (13), 1801–1804. https://doi.org/10.1039/D2CC06473A

(12) Padial, N. M.; Castells-Gil, J.; Almora-Barrios, N.; Romero-Angel, M.; da Silva, I.; Barawi, M.; García-Sánchez, A.; de la Peña O'Shea, V. A.; Martí-Gastaldo, C. Hydroxamate Titanium–Organic Frameworks and the Effect of Siderophore-Type Linkers over Their Photocatalytic Activity. *J. Am. Chem. Soc.* **2019**, *141* (33), 13124–13133. https://doi.org/10.1021/jacs.9b04915

(13) Yan, Q.; Wang, J.; Zhang, L.; Liu, J.; Wahiduzzaman, M.; Yan, N.; Yu, L.; Dupuis, R.; Wang, H.; Maurin, G.; Hirscher, M.; Guo, P.; Wang, S.; Du, J. A Squarate-Pillared Titanium Oxide Quantum Sieve towards Practical Hydrogen Isotope Separation. *Nat. Commun.* **2023**, *14* (1), 4189. https://doi.org/10.1038/s41467-023-39871-x

(14) Naghdi, S.; Cherevan, A.; Giesriegl, A.; Guillet-Nicolas, R.; Biswas, S.; Gupta, T.; Wang, J.; Haunold, T.; Bayer, B. C.; Rupprechter, G.; Toroker, M. C.; Kleitz, F.; Eder, D. Selective Ligand Removal to Improve Accessibility of Active Sites in Hierarchical MOFs for Heterogeneous Photocatalysis. *Nat. Commun.* **2022**, *13* (1), 282. https://doi.org/10.1038/s41467-021-27775-7

(15) International Union of Pure and Applied Chemistry. Commission on Equilibrium Data. *Ionisation Constants of Organic Acids in Aqueous Solution*, E. P. Serjeant and B. Dempsey.; Pergamon Press: Oxford, 1979.

(16) Zheng, J.; Lafontant-Joseph, O. IUPAC/Dissociation-Constants: V2-1. **2024**. https://doi.org/10.5281/zenodo.12708216

(17) Assi, H.; Pardo Pérez, L. C.; Mouchaham, G.; Ragon, F.; Nasalevich, M.; Guillou, N.; Martineau, C.; Chevreau, H.; Kapteijn, F.; Gascon, J.; Fertey, P.; Elkaim, E.; Serre, C.; Devic, T. Investigating the Case of Titanium(IV) Carboxyphenolate Photoactive Coordination Polymers. *Inorg. Chem.* **2016**, *55* (15), 7192–7199. https://doi.org/10.1021/acs.inorgchem.6b01060

(18) Smolders, S.; Willhammar, T.; Krajnc, A.; Sentosun, K.; Wharmby, M. T.; Lomachenko, K. A.; Bals, S.; Mali, G.; Roeffaers, M. B. J.; De Vos, D. E.; Bueken, B. A Titanium(IV)-Based Metal–Organic Framework Featuring Defect-Rich Ti-O Sheets as an Oxidative Desulfurization Catalyst. *Angew. Chem. Int. Ed.* **2019**, *58* (27), 9160–9165. https://doi.org/10.1002/anie.201904347

(19) Biesinger, M. C.; Lau, L. W. M.; Gerson, A. R.; Smart, R. St. C. Resolving Surface Chemical States in XPS Analysis of First Row Transition Metals, Oxides and Hydroxides: Sc, Ti, V, Cu and Zn. *Appl. Surf. Sci.* **2010**, *257* (3), 887–898. https://doi.org/10.1016/j.apsusc.2010.07.086

(20) Holten-Andersen, N.; Jaishankar, A.; Harrington, M. J.; Fullenkamp, D. E.; DiMarco, G.; He, L.; McKinley, G. H.; Messersmith, P. B.; Lee, K. Y. C. Metal-Coordination: Using One of Nature's Tricks to Control Soft Material Mechanics. *J. Mater. Chem. B* **2014**, *2* (17), 2467–2472. https://doi.org/10.1039/C3TB21374A

(21) Capurso, M.; Radivoy, G.; Nador, F.; Dorn, V. A Computational and Spectroscopic Approach to Elucidate the Coordination Structures in Iron–Catechol Polymers. *Chem. Proc.* **2024**, *16* (1), 7. https://doi.org/10.3390/ecsoc-28-20208

(22) Fulmer, G. R.; Miller, A. J. M.; Sherden, N. H.; Gottlieb, H. E.; Nudelman, A.; Stoltz, B. M.; Bercaw, J. E.; Goldberg, K. I. NMR Chemical Shifts of Trace Impurities: Common Laboratory Solvents, Organics, and Gases in Deuterated Solvents Relevant to the Organometallic Chemist. *Organometallics* **2010**, *29* (9), 2176–2179. https://doi.org/10.1021/om100106e

(23) Knapp, J. E.; Ortín-Rubio, B.; Heck, F.; Gjorgjevikj, K.; Sleptsova, A.; Krause, S.; Bette, S.; Lotsch, B. V. Dynamic Breathing Behaviour of the Titanium-Based Metal–Organic Framework NTU-9 upon Adsorption of Water and Organic Solvents. *Chem. Sci.* **2025**, *16* (30), 13646–13654. https://doi.org/10.1039/D5SC02585K

(24) Gao, J.; Miao, J.; Li, P.-Z.; Teng, W. Y.; Yang, L.; Zhao, Y.; Liu, B.; Zhang, Q. A P-Type Ti(IV)-Based Metal–Organic Framework with Visible-Light Photo-Response. *Chem. Commun.* **2014**, *50* (29), 3786–3788. https://doi.org/10.1039/C3CC49440C

(25) Qu, J.-X.; Fu, Y.-M.; Meng, X.; He, Y.-O.; Sun, H.-X.; Yang, R.; Wang, H.-N.; Su, Z.-M. A Porous Ti-Based Metal–Organic Framework for CO_2 Photoreduction and Imidazole-Dependent Anhydrous Proton Conduction. *Chem. Commun.* **2023**, *59* (8), 1070–1073. https://doi.org/10.1039/d2cc06214c

(26) Dvoranová, D.; Barbieriková, Z.; Brezová, V. Radical Intermediates in Photoinduced Reactions on TiO_2 (An EPR Spin Trapping Study). *Molecules* **2014**, *19* (11), 17279–17304. https://doi.org/10.3390/molecules191117279

(27) Myakala, S. N.; Ladisich, M.; Ayala, P.; Rabl, H.; Batool, S.; Elsaesser, M. S.; Cherevan, A.; Eder, D. Harnessing a Ti-Based MOF for Selective Adsorption and Visible-Light-Driven Water Remediation. *J. Mater. Chem. A* **2024**, *12* (31), 19924–19934. https://doi.org/10.1039/D4TA01967A
